# Electrostatics in Materials Revisited: The Case of Free Charges Combined with Linear, Homogeneous, and Isotropic Dielectrics

**DOI:** 10.3390/ma17205046

**Published:** 2024-10-15

**Authors:** Dimosthenis Stamopoulos

**Affiliations:** Department of Physics, School of Science, National and Kapodistrian University of Athens, Zografou Panepistimioupolis, 15784 Athens, Greece; densta@phys.uoa.gr

**Keywords:** electrostatics in materials, dielectrics, electric polarization, electric susceptibility

## Abstract

Here we revisit the electrostatics of material systems comprising of free charges and linear, homogeneous, and isotropic (LHI) dielectrics. We focus on **D**(**r**) suggesting that this is the *primary* vector field of electrostatics. We show that **D**(**r**) is sufficient to conceptually describe all underlying physics and to mathematically accomplish all necessary calculations, beforehand, independently of the *secondary* vector fields **P**(**r**) and **E**(**r**) that, if needed, can be easily calculated from **D**(**r**). To this effect, we introduce a P-D electric susceptibility, χ_ε_, with $-1\leq \chi_{\varepsilon }\leq 0$, that couples linearly **P**(**r**) with **D**(**r**) (instead of the standard P-E electric susceptibility, χ_e_, with $0\leq \chi_{e}<\infty$, that couples linearly **P**(**r**) with **E**(**r**)). This concept restores the somehow misleading causality/feedback between **P**(**r**) and **E**(**r**) of the standard formulation, captures efficiently the underlying physics, enables electrostatics to obtain a form analogous to that of magnetostatics, and facilitates analytical/computational calculations in relevant systems. To document these claims, we provide technical means, among others, the *free* scalar potential, $U_{f}\left(r\right)$, and clarify the conditions that enable the calculation of **D**(**r**) on a standalone basis, directly from the *free* charge density, $\rho_{f}$, and the electric susceptibility, χ_ε_, of the LHI dielectrics. Our concept sets interesting perspectives for the treatment of all dielectrics.

## 1. Introduction

Electromagnetism is a cornerstone theory of classical physics that ever since its conceptualization [1,2,3] still develops under our need to theoretically explain relevant phenomena and to accurately design useful applications [4,5,6,7]. Maxwell’s equations constitute the fundamental set of relations for the mathematical description of electromagnetism [8,9,10,11,12,13,14,15]. Apart from electrical conductivity that relates to the existence of *free* charges and currents, another key property that differentiates electromagnetism in matter, from vacuum, is the electric and magnetic polarization, whether this is exogenously imposed (induced polarization) or endogenously established (permanent polarization) [8,9,10,11,12,13,14,15]. In both cases, the concept of *bound* charges and currents can be employed to model the polarization of matter [16,17,18,19]. Ultimately, matter can be completely replaced by these *free* and *bound* charges and currents that, now, exist in vacuum.

In recent decades, advancements in materials science and engineering have expanded the boundaries of what we used to call “matter”. For instance, “matter” traditionally referred to ordinary single/poly-crystalline and amorphous materials consisting of elemental atoms. This ordinary “matter” is produced at the laboratory in various forms (three-dimensional bulk, low-dimensional films, rods and spheres, etc.) by means of a variety of techniques (solid and wet chemistry, sol-gel, thermal evaporation, dc/rf sputtering, etc.). Nowadays, “matter” also includes extraordinary metamaterials consisting of simple/complex building units, of sizes ranging from nanometers to centimeters, that are carefully (self)assembled to form artificial structures. This extraordinary “matter” is produced by means of relatively standard or even highly sophisticated manipulation/patterning techniques (cantilever-based manipulation and lithography, photochemical etching, electron-beam lithography, etc.). It is probably not unexpected that “matter” of such diversity does not ‘behave homogeneously’ in respect to electric and magnetic polarization. Inevitably, depending on the size and the physical properties of the employed constituents and the characteristic length scales of “matter” that they assemble, the distinction between *free* and *bound* charges and currents can be quite unclear [20]. Apparently, a variety of physical concepts and mathematical tools should be available to model, both reliably and flexibly, the electric and magnetic polarization of such multifaceted “matter”.

Probably, the most profound endogenous ‘asymmetry’ between electricity and magnetism stems from the constitutive relations that define the respective polarization, electric, P(r), and magnetic, M(r). Specifically, the electric polarization, P(r), is defined through the so-called electric field, E(r), that relates to both *free* and *bound* charges, while the so-called auxiliary field, D(r), (else, electric displacement) that relates to (however, not solely depends on) *free* charges is introduced through the fundamental relation $D\left(r\right)=\varepsilon_{0}E\left(r\right)+P(r)$. On the contrary, the magnetic polarization, M(r), is defined through the so-called auxiliary field, H(r), (else, magnetizing field) that relates to (however, not solely depends on) *free* currents, while the so-called magnetic field, $B\left(r\right)$, that relates to both *free* and *bound* currents, appears in the fundamental relation $B\left(r\right)=\mu_{0}(H\left(r\right)+M\left(r\right))$. Obviously, a complete analogy between electricity and magnetism requires that P(r) should be defined through D(r).

Here we discuss these issues for the electrostatics of systems comprising of *free* charges and linear, homogeneous, and isotropic (LHI) dielectrics. The latter constitute a wide class of materials with many applications. Also, though many dielectrics are not LHI strictly speaking, they exhibit a quasi linear/homogeneous behavior so that they can be approximated as LHI quite effectively. We introduce a new formulation between the electric polarization, P(r), and the electric displacement, $D\left(r\right)$, that are *directly* coupled in a linear fashion through a P-D electric susceptibility, χ_ε_ (to be distinguished from the standard P-E one, χ_e_, that couples linearly P(r) and $E\left(r\right)$). The underlying physics of the direct, linear constitutive relation between P(r) and $D\left(r\right)$ through χ_ε_ is discussed in detail. Since $D\left(r\right)$ is the *primary* vector field that should be obtained beforehand, independently of the *secondary* P(r) and $E\left(r\right)$, we provide thorough technical documentation on its mathematical calculation for any generic system that comprises of *free* charges and LHI dielectric materials. The introduction of the new formulation of the electric susceptibility, χ_ε_, and polarization, P(r), in respect to the *primary* vector field, $D\left(r\right)$, restores mathematical and conceptual flaws which exist in the standard formulation based on the *secondary* vector field, $E\left(r\right)$. Also, it establishes the analogy in the definition of electric and magnetic polarizations, $P\left(r\right)$ and $M\left(r\right)$, in respect to the vector fields, $D\left(r\right)$ and $H\left(r\right)$. The foreground mathematics and the underlying physics of the *primary* vector field, $D\left(r\right)$, and of the new formulation, ‘P-D, χ_ε_’, are discussed in detail. Representative problems referring to standard geometries met in applications are discussed for the two formulations on a comparative basis, to document their quantitative consistency and highlight the physical advancements and possible technical advantages of the new formulation, ‘P-D, χ_ε_’, in respect to the standard one, ‘P-E, χ_e_’.

## 2. Background

In LHI dielectrics, based on the fundamental relation $D\left(r\right)=\varepsilon_{0}E\left(r\right)+P(r)$ and the standard formulation, we have the following set of basic relations that couples linearly all vector fields $D\left(r\right)$, $E\left(r\right),$ and P(r)

(1) $$P\left(r\right)=\chi_{e}\varepsilon_{0}E\left(r\right)$$

(2) $$D\left(r\right)=(1+\chi_{e})\varepsilon_{0}E\left(r\right)=\varepsilon_{r}\varepsilon_{0}E\left(r\right)=\varepsilon E\left(r\right)$$

(3) $$P\left(r\right)=(\chi_{e}/(\chi_{e}+1))D\left(r\right)=((\varepsilon_{r}-1)/\varepsilon_{r})D\left(r\right)$$

where $\chi_{e}$, $\varepsilon_{r},$ and ε refer to the properties of the material, that is electric susceptibility, relative permittivity, and permittivity, respectively. For these the following relations hold

(4) $$0\leq \chi_{e}<\infty ,$$

(5) $$\varepsilon_{r}=\chi_{e}+1,\;1\leq \varepsilon_{r}<\infty$$

(6) $$\mathrm{and}\;\varepsilon =\varepsilon_{0}\varepsilon_{r}$$

where $\varepsilon_{0}$ is the permittivity of free space [8,9,10,11,12,13,14,15]. The fact that in LHI dielectrics all three vector fields, $D\left(r\right)$, $P\left(r\right),$ and $E\left(r\right)$ are linearly coupled through constant coefficients has a beneficial consequence; their divergence and curl are always known (given that we have enough information on the respective sources/charge densities). Referring to the divergence, the following relations hold:

(7) $$\nabla \cdot D\left(r\right)=\rho_{f}\left(r\right)$$

(8) $$\nabla \cdot P\left(r\right)=-\rho_{b}\left(r\right)$$

(9) $$\nabla \cdot E\left(r\right)=\rho \left(r\right)/\varepsilon_{0}.$$

The linear coupling of $D\left(r\right)$, $P\left(r\right),$ and $E\left(r\right)$ given by the above relations is directly imprinted onto the respective *free*, $\rho_{f}(r)$, *bound*, $\rho_{b}(r)$, and *total*, $\rho \left(r\right)=\rho_{f}\left(r\right)+\rho_{b}(r)$ charge densities through

(10) $$\rho_{b}\left(r\right)=-\chi_{e}\rho \left(r\right)$$

(11) $$\rho_{b}\left(r\right)=-{\left(\frac{\chi_{e}}{\chi_{e}+1}\right)\rho }_{f}\left(r\right)=-{\left(\frac{\chi_{e}}{\varepsilon_{r}}\right)\rho }_{f}\left(r\right)$$

(12) $$\rho_{f}\left(r\right)=\left(\chi_{e}+1\right)\rho \left(r\right)=\varepsilon_{r}\rho \left(r\right).$$

Referring to the curl, in any dielectric the following relations hold

(13) $$\nabla \times E\left(r\right)=0,$$

(14) $$\nabla \times D\left(r\right)=\nabla \times P\left(r\right).$$

Specifically, in LHI dielectrics, at the *interior,* all three vector fields are, apparently, irrotational:

(15) $$\nabla \times D\left(r\right)=\nabla \times P\left(r\right)=\nabla \times E\left(r\right)=0.$$

However, at *interfaces* of distinct LHI dielectrics, while $E\left(r\right)$ preserves its irrotational character, $D\left(r\right)$ and $P\left(r\right)$ may not (see below) so that:

(16) $$\nabla \times D\left(r\right){|}_{S^{/}}=\nabla \times P\left(r\right){|}_{S^{/}}.$$

Accordingly, once in LHI dielectrics, both the divergence relations (7)–(9), and the curl relations (15)–(16), are known for all $D\left(r\right)$, $P\left(r\right),$ and $E\left(r\right)$, based on the Helmholtz theorem [9,12,13,14,21,22,23,24], any one of these vector fields can be employed, equally well, to tackle the mathematical problem. Then, the other two vector fields can be calculated easily from the, arbitrarily chosen, first one through relations (1)–(3). Nevertheless, on physical grounds, the *free* charge density, $\rho_{f}(r)$, is the cause that induces any *bound* charge density, $\rho_{b}(r)$, that in turn adds to $\rho_{f}(r)$ to give the *total* charge density, $\rho \left(r\right)=\rho_{f}\left(r\right)+\rho_{b}(r)$. Thus, in LHI dielectrics, neither $\rho_{b}(r)$ nor ρ(r) is a standalone charge entity that can serve as a *primary* source of fields; $\rho_{f}\left(r\right)$ is always needed for $\rho_{b}(r)$ to appear and for $\rho \left(r\right)$ to complete. It is natural then to consider $\rho_{f}\left(r\right)$ as the *primary* charge density/source of fields, and $\rho_{b}\left(r\right)$ and $\rho \left(r\right)$ as *secondary* charge densities/sources of fields. The same reasoning should hold for the respective vector fields; $D\left(r\right)$ that relates to $\rho_{f}\left(r\right)$ through relation (7) could be considered as the *primary* vector field of electrostatics, while both P(r) and $E\left(r\right)$ that are induced by $D\left(r\right)$ and relate to $\rho_{b}\left(r\right)$ and $\rho \left(r\right)$ through relations (8) and (9), respectively, can be considered as *secondary* vector fields.

Interestingly, the linear relation (2) implies that instead of the standard one (1), where $\chi_{e}$ is the P-E electric susceptibility ($0\leq \chi_{e}<\infty )$, an alternative, effective P-D electric susceptibility, could be defined, as well, that couples *directly* P(r) and $D\left(r\right)$. Indeed, from a clearly algebraic point of view, we could define the effective P-D electric susceptibility, $\chi_{ef}=\left(\chi_{e}/(\chi_{e}+1\right))$, through $P\left(r\right)=\chi_{ef}D\left(r\right),$ (where $0\leq \chi_{ef}\leq 1$, since $0\leq \chi_{e}<\infty$) and abandon the use of the P-E electric susceptibility, $\chi_{e}$, once and for all. Then, based on the fundamental relation $D\left(r\right)=\varepsilon_{0}E\left(r\right)+P(r)$, the vector field $E\left(r\right)$ should be coupled with P(r) and $D\left(r\right)$ through the linear relations $E\left(r\right)=((1-\chi_{ef})/\chi_{ef})(1/\varepsilon_{0})P\left(r\right)$ and $E\left(r\right)=(1-\chi_{ef})(1/\varepsilon_{0})D\left(r\right)$, respectively. Obviously, before we adopt any strategy of this kind, we should ensure that the effective P-D electric susceptibility, $\chi_{ef}$, captures consistently the underlying physics of the system in a way equivalent (if not, even, advantageous) to that of the standard P-E electric susceptibility, $\chi_{e}$.

The above discussion proves two crucial characteristics which, though inherent in LHI materials, *are always overlooked in the standard formulation employed today*. First, since the divergence and curl of the *primary* vector field D(r) are known from the very beginning (relations (7) and (15)–(16)), based on the Helmholtz theorem, D(r) can be calculated beforehand, that is independently of the *secondary* vector fields P(r) and $E\left(r\right)$. Technically, D(r) seems to depend exclusively on the *free* charge density through relation (7). However, the properties of the LHI materials (i.e., $\chi_{e}$) can be imprinted onto D(r), as well, through the relevant boundary conditions that should be satisfied at the interfaces of discontinuous dielectric media. This point is clearly elucidated below and in Appendix A.1 of the Appendix A. Second, once D(r) is known, the electric polarization, P(r), is ultimately coupled to it through relation (3). As discussed above, this evidences that there is still some room to express the linear relation between P(r) and $D\left(r\right)$ in a *direct* way, $P\left(r\right)=\chi_{ef}D\left(r\right)$, without using $E\left(r\right)$ as an intermediate field. Ultimately, this could help us to bypass any conceptually misleading argumentation and mathematical complications on the causality/feedback between P(r) and $E\left(r\right)$ (see [8] pages 68 and 76; [13] page 186) and to restore the “asymmetry” between electrostatics and magnetostatics in respect to the polarization properties.

## 3. Definition of a P-D Electric Susceptibility, χ_ε_, and Constitutive Relations Between the Vector Fields D(r), P(r), and E(r)

Following the above considerations, here we introduce a new formulation between the electric polarization, P(r), and the electric displacement, $D\left(r\right)$, that are *directly* coupled in a linear fashion through a P-D electric susceptibility, χ_ε_. We recall that the standard P-E electric susceptibility, χ_e_, couples the electric polarization, P(r), and the electric field, $E\left(r\right)$, relation (1). To clearly distinguish the two cases, we use the notation ‘P-D, χ_ε_’ for the one introduced here and ‘P-E, χ_e_’ for the standard one, currently in use (notice the different subscripts ‘ε’ and ‘e’).

Starting from the fundamental relation $D\left(r\right)=\varepsilon_{0}E\left(r\right)+P\left(r\right),$ we introduce the constitutive relation $P\left(r\right)=-\chi_{\varepsilon }D(r)$, else $-P\left(r\right)=\chi_{\varepsilon }D\left(r\right),$ and easily get $D\left(r\right)=(\varepsilon_{0}/(1+\chi_{\varepsilon }))E\left(r\right)$, where we employ the newly defined P-D electric susceptibility, $\chi_{\varepsilon }$. Likewise, by introducing the *reverse* electric polarization, $\tilde{P}\left(r\right)=-P(r)$, relation $P\left(r\right)=-\chi_{\varepsilon }D(r)$ becomes $\tilde{P}\left(r\right)=\chi_{\varepsilon }D(r)$. The use of the *reverse* electric polarization field, $\tilde{P}\left(r\right)=-P\left(r\right)$, instead of $P\left(r\right)$ may probably seem as a weird algebraic maneuver. However, the introduction of $\tilde{P}\left(r\right)=-P(r)$ is based on the physical fact that $\tilde{P}\left(r\right)$ directly relates to the so-called *internal* electric field, $E_{int}(r)$, produced by the *bound* charges (else, depolarizing field (see [8] pages 93–96), or self-field (see [14] pages 127, 158, and § 6.6.2; [15] page 24)), through $\tilde{P}\left(r\right)=-P\left(r\right)=\varepsilon_{0}E_{int}(r)$ [25]. Thus, $\tilde{P}\left(r\right)$ carries much physical information on a standalone basis (more details are given below). By using the above relations, we easily obtain the electric field through $E\left(r\right)=(1/\varepsilon_{\varepsilon })D\left(r\right)$, where we defined $\varepsilon_{r\varepsilon }=1/(1+\chi_{\varepsilon })$ the P-D relative permittivity and $\varepsilon_{\varepsilon }=\varepsilon_{0}\varepsilon_{r\varepsilon }=\varepsilon_{0}/(1+\chi_{\varepsilon })$ the P-D permittivity.

No matter how we define the scalar coefficients that in LHI dielectrics couple linearly $D\left(r\right)$, $P\left(r\right),$ and $E\left(r\right)$, these vector fields should be unaltered between different formulations. Consequently, through a comparison of $D\left(r\right)=(\varepsilon_{0}/(1+\chi_{\varepsilon }))E\left(r\right)$ with the standard relation (2) that is based on the P-E electric susceptibility, $\chi_{e}$, we see that the newly defined P-D electric susceptibility, $\chi_{\varepsilon }$, relates to $\chi_{e}$ through $1+\chi_{\varepsilon }=1/(1+\chi_{e})$, else $\chi_{\varepsilon }=-\chi_{e}/(1+\chi_{e})$. Obviously, the newly defined P-D electric susceptibility, $\chi_{\varepsilon }$, ranges within $-1\leq \chi_{\varepsilon }\leq 0$, thus spanning the entire range $0\leq \chi_{e}<\infty$ of the standard P-E electric susceptibility, $\chi_{e}$, as it should. Notably, the respective P-D relative permittivity, $\varepsilon_{r\varepsilon }$, ranges within $1\leq \varepsilon_{r\varepsilon }<\infty$, that is the exact same range, $1\leq \varepsilon_{r}<\infty$, of the standard P-E relative permittivity, $\varepsilon_{r}$.

Summarizing, in this new formulation ‘P-D, χ_ε_’, the linear relations that couple the *primary* field $D\left(r\right)$ with the *secondary* fields $\tilde{P}\left(r\right)=-P\left(r\right)$ and $E\left(r\right)$ are the following:

(17) $$\tilde{P}\left(r\right)=-P\left(r\right)=\chi_{\varepsilon }D(r)$$

(18) $$E\left(r\right)=((1+\chi_{\varepsilon })/\varepsilon_{0})D\left(r\right)=(1/\varepsilon_{0}\varepsilon_{r\varepsilon })D\left(r\right)=(1/\varepsilon_{\varepsilon })D\left(r\right)$$

(19) $$\tilde{P}\left(r\right)=-P\left(r\right)=(\chi_{\varepsilon }/(1+\chi_{\varepsilon }))\varepsilon_{0}E\left(r\right)=(1-\varepsilon_{r\varepsilon })\varepsilon_{0}E\left(r\right)$$

where $\chi_{\varepsilon }$, $\varepsilon_{r\varepsilon },$ and $\varepsilon_{\varepsilon }$ refer to the P-D constants, that is electric susceptibility, relative permittivity, and permittivity, respectively, with:

(20) $$-1\leq \chi_{\varepsilon }\leq 0,$$

(21) $$\varepsilon_{r\varepsilon }={\left(1+\chi_{\varepsilon }\right)}^{-1},\;1\leq \varepsilon_{r\varepsilon }<\infty$$

(22) $$\mathrm{and}\;\varepsilon_{\varepsilon }=\varepsilon_{0}\varepsilon_{r\varepsilon }.$$

Referring to the divergence of the three vector fields, we have

(23) $$\nabla \cdot D\left(r\right)=\rho_{f}\left(r\right)$$

(24) $$\nabla \cdot \tilde{P}\left(r\right)=\nabla \cdot (-P\left(r\right))=\rho_{b}\left(r\right)$$

(25) $$\nabla \cdot E\left(r\right)=\rho \left(r\right)/\varepsilon_{0}.$$

We see that relations (7) and (9) still hold for $D\left(r\right)$ and $E\left(r\right)$, respectively, while relation (8) is adjusted to the newly defined *reverse* electric polarization $\tilde{P}\left(r\right)=-P\left(r\right)$.

Most importantly, by using relations (23)–(25), the linear coupling of $D\left(r\right)$, $\tilde{P}\left(r\right)=-P\left(r\right),$ and $E\left(r\right)$ of relations (17)–(19) is directly imprinted onto the respective *free*, $\rho_{f}(r)$, *bound*, $\rho_{b}(r)$, and *total*, $\rho \left(r\right)=\rho_{f}\left(r\right)+\rho_{b}(r)$ charge densities as:

(26) $$\rho_{b}\left(r\right)=\chi_{\varepsilon }\rho_{f}\left(r\right)=-(({\varepsilon_{r\varepsilon }-1)/\varepsilon_{r\varepsilon })\rho }_{f}\left(r\right)$$

(27) $$\rho_{f}\left(r\right)=\rho \left(r\right)/(1+\chi_{\varepsilon })=\varepsilon_{r\varepsilon }\rho \left(r\right)$$

(28) $$\rho_{b}\left(r\right)=(\chi_{\varepsilon }/(1+\chi_{\varepsilon }))\rho \left(r\right)=(1-\varepsilon_{r\varepsilon })\rho \left(r\right).$$

Regarding the curl, in any dielectric, the following relations should hold for the four vector fields:

(29) $$\nabla \times E\left(r\right)=0,$$

(30) $$\nabla \times D\left(r\right)=\nabla \times P\left(r\right)=\nabla \times (-\tilde{P}\left(r\right)).$$

Specifically, in LHI dielectrics, at the *interior,* all four vector fields are, apparently, irrotational:

(31) $$\nabla \times D\left(r\right)=\nabla \times P\left(r\right)=\nabla \times (-\tilde{P}\left(r\right))=\nabla \times E\left(r\right)=0.$$

However, at *interfaces* of distinct LHI dielectrics, while $E\left(r\right)$ preserves its irrotational character, $D\left(r\right)$ and $\tilde{P}\left(r\right)=-P\left(r\right)$ may not (see below), so that:

(32) $$\nabla \times D\left(r\right){|}_{S^{/}}=\nabla \times P\left(r\right){|}_{S^{/}}=\nabla \times (-\tilde{P}\left(r\right)){|}_{S^{/}}.$$

Finally, due to the quantitative equivalence of the two formulations, the respective electric susceptibilities $\chi_{e}$ and $\chi_{\varepsilon }$ should relate through:

(33) $$1+\chi_{\varepsilon }=1/(1+\chi_{e}),$$

(34) $$\mathrm{else}\;\chi_{\varepsilon }=-\chi_{e}/(1+\chi_{e}),$$

(35) $$\mathrm{else}\;\chi_{e}=-\chi_{\varepsilon }/(1+\chi_{\varepsilon }).$$

Details of the quantitative equivalence of the two formulations are given below in Section 7 and in Appendix A.2 and Appendix A.3 of the Appendix A.

## 4. Considerations on the Physics of the P-D Electric Susceptibility, χ_ε_, Formulation

### 4.1. Conceptually Misleading Causality/Feedback Between $P\left(r\right)$ and $E\left(r\right)$

In the standard formulation of the P-E electric susceptibility, χ_e_, the electric polarization, $P\left(r\right)$, couples to the electric field, $E\left(r\right)$, through relation (1). Accordingly, the electric field induces an electric polarization that in turn contributes to the electric field and so on. Thus, in the P-E, χ_e_, formulation, the distinction between the cause and causal is somehow obscured by this strange feedback. This is addressed in many textbooks. For instance, in page 68 of [8], this is described as an *“…awkward situation: the polarization of the dielectric depends on the total electric field in the medium, but a part of the electric field is produced by the dielectric itself…”* (see page 76 of [8], as well). Also, in page 186 of [13], it is mentioned that *“…the external field will polarize the material, and this polarization will produce its own field, which then contributes to the total field, and this in turn modifies the polarization, which…Breaking out this infinite regress is not always easy”*. In Section 7 below and in Appendix A.2 of the Appendix A we show that, indeed, this *“infinite regress”* between $P\left(r\right)$ and $E\left(r\right)$ is mathematically described by an infinite geometric series that, quaintly, seems to converge for the entire range $0\leq \chi_{e}<\infty$ (though, it should not!) to the standard result obtained by other methods. Our P-D, $\chi_{\varepsilon }$, description is immune to these conceptual and mathematical complications.

Interestingly, to bypass the *“awkward situation”* described above, in page 186 of [13], it is proposed that *“The simplest way is to begin with the displacement, at least in those cases where **D** can be deduced directly from the free charge distribution”*. In the present work, we follow this exact suggestion: by using the P-D, $\chi_{\varepsilon }$, formulation, we focus on the primary vector field of electrostatics, $D\left(r\right)$, and provide mathematical means that enable us to deduce it in all cases beforehand, independently of the secondary vector fields, $\tilde{P}\left(r\right)=-P\left(r\right)$ and $E\left(r\right)$, by taking into account both the free charges and the dielectric properties of the LHI materials. Then, $\tilde{P}\left(r\right)=-P\left(r\right)$ and $E\left(r\right)$, can be obtained from $D\left(r\right)$ through relations (17) and (18) (see Section 7 below and Appendix A.1 and Appendix A.2 of the Appendix A).

### 4.2. Direct and Concise Description of the Physics of the LHI Dielectrics

The P-D, $\chi_{\varepsilon }$, formulation describes the underlying physics of the LHI dielectrics in a clear way. First, in this scheme, the induced *bound* charge density, $\rho_{b}\left(r\right)$, is linearly coupled to the *free* one, $\rho_{f}\left(r\right)$, in a *direct* way through relation (26). Since $-1\leq \chi_{\varepsilon }\leq 0$ (relation (20)), we see that the induced $\rho_{b}\left(r\right)$ is always of opposite sign to $\rho_{f}\left(r\right)$, thus *bound* charges screen *free* ones, as expected. In addition, the lowest value that $\chi_{\varepsilon }$ can obtain, $\chi_{\varepsilon }=-1$, limits physically the maximum absolute value that the *bound* charge density can obtain; $\rho_{b}\left(r\right)$ should never exceed the *free* charge density, $\rho_{f}\left(r\right)$, since the electrically neutral state (where $\rho \left(r\right)=\rho_{f}\left(r\right)+\rho_{b}\left(r\right)=\rho_{f}\left(r\right)-\rho_{f}\left(r\right)=0$), has the lowest electrostatic energy. Second, the new scheme reveals straightforwardly the well-known fact that dielectrics tend to lower the electric field, $E\left(r\right)$, in their interior. Indeed, the fact that $-1\leq \chi_{\varepsilon }\leq 0\;$ and $1\leq \varepsilon_{r\varepsilon }={\left(1+\chi_{\varepsilon }\right)}^{-1}<\infty$ (relations (20)–(22)) ensures that the electric field, $E\left(r\right)$, given by relation (18) is always lower inside a dielectric, ultimately getting $E\left(r\right)=0$ when $\chi_{\varepsilon }=-1$ ($\varepsilon_{r\varepsilon },\varepsilon \to \infty$). This fact is also evidenced by the standard P-E, $\chi_{e}$, formulation employed today. Indeed, since $0\leq \chi_{e}<\infty$ and $1\leq \varepsilon_{r}=1+\chi_{e}<\infty$ (relations (4)–(6)), it is obvious that $E\left(r\right)$ given by relation (2) is always lower inside the dielectric, eventually becoming $E\left(r\right)=0$ when $\chi_{e}\to \infty$ ($\varepsilon_{r},\varepsilon \to \infty$).

As already stated above, the introduction of the *reverse* electric polarization field, $\tilde{P}\left(r\right)=-P\left(r\right)$, is based on the physical fact that it relates to the so-called *internal* electric field, $E_{int}(r)$, through $\tilde{P}\left(r\right)=-P\left(r\right)=\varepsilon_{0}E_{int}(r)$ [25]. More explicitly, $E_{int}(r)$ is produced by the *bound* electric charges that, for the case of the LHI dielectrics discussed here, reside exclusively at the interfaces between distinct media of different dielectric properties. Notably, $E_{int}(r)$ of the *bound* electric charges acts against the *external* electric field, $E_{ext}(r)$, of the *free* electric charges, thus it lowers the electric field, E(r), inside dielectrics, and tends to destabilize polarization order as well (see Section 7 below and Appendix A.2 of the Appendix A). Interestingly, the divergence of the *reverse* electric polarization $\tilde{P}\left(r\right)=-P\left(r\right)$ is compatible to that of $E\left(r\right)$ and $D\left(r\right)$; these vector fields point outwards from a positive charge density/source, while they point inwards to a negative charge density/sink ($\nabla \cdot \tilde{P}\left(r\right)=\rho_{b}\left(r\right)$, $\nabla \cdot D\left(r\right)=\rho_{f}\left(r\right),$ and $\nabla \cdot E\left(r\right)=\rho \left(r\right)/\varepsilon_{0}$).

### 4.3. Symmetry in the Electrostatics and Magnetostatics of LHI Materials

The P-D, $\chi_{\varepsilon }$, formulation of the LHI dielectrics is based on the consideration that $D\left(r\right)$ is a *primary* vector field that can be calculated beforehand, independently of the *secondary* vector fields $P\left(r\right)$ and $E\left(r\right)$ (for instance, by using one of the strategies discussed below). Then, $P\left(r\right)$ and $E\left(r\right)$ can be easily found since they couple linearly to $D\left(r\right)$ through $\tilde{P}\left(r\right)=-P\left(r\right)=\chi_{\varepsilon }D(r)$ and $E\left(r\right)=((1+\chi_{\varepsilon })/\varepsilon_{0})D\left(r\right)=(1/\varepsilon_{0}\varepsilon_{r\varepsilon })D\left(r\right)=(1/\varepsilon_{\varepsilon })D\left(r\right),$ (where $-1\leq \chi_{\varepsilon }\leq 0$ and $1\leq \varepsilon_{r\varepsilon }={\left(1+\chi_{\varepsilon }\right)}^{-1}<\infty$). In analogy, in LHI diamagnetic materials, $H\left(r\right)$ can be considered as a *primary* vector field that can be calculated beforehand, independently of the *secondary* vector fields $M\left(r\right)$ and $B\left(r\right)$ (for instance, by means similar to the ones discussed below for $D\left(r\right)$). Notably, in the standard M-H, $\chi_{m}$, formulation of magnetostatics, $M\left(r\right)$ and $B\left(r\right)$ couple linearly to $H\left(r\right)$ through $M\left(r\right)=\chi_{m}H(r)$ and $B\left(r\right)=\mu_{0}(1+\chi_{m})H\left(r\right)=\mu_{0}\mu_{r}H\left(r\right)=\mu H\left(r\right)$ (where $-1\leq \chi_{m}\leq 0$ and $0\leq \mu_{r}=1+\chi_{m}\leq 1$). In this sense, in LHI materials, electricity ($E\left(r\right)=\left(1/\varepsilon_{0}\right)\left(D\left(r\right)+(-P\left(r\right))\right)=\left(1/\varepsilon_{0}\right)(D\left(r\right)+\tilde{P}\left(r\right))$) obtains a somehow symmetric formulation to that of magnetism ($B\left(r\right)=\mu_{0}(H\left(r\right)+M\left(r\right))$).

The above analogy between the formulations, P-D, $\chi_{\varepsilon }$, and M-H, $\chi_{m}$, of the dielectric and diamagnetic LHI materials is neither coincidental nor artificial. It clearly evidences a basic property that dielectric and diamagnetic materials have in common: they screen their interior from the external stimulus, the electric and magnetic field, respectively. Indeed, the P-D, $\chi_{\varepsilon }$, scheme introduced here for the dielectric materials, $\tilde{P}\left(r\right)=-P\left(r\right)=\chi_{\varepsilon }D(r)$ and $E\left(r\right)=(1/\varepsilon_{0})\left(D\left(r\right)+\tilde{P}\left(r\right)\right)=(1/\varepsilon_{0})\left(D\left(r\right)+\chi_{\varepsilon }D\left(r\right)\right)=(1/\varepsilon_{0})\left(1+\chi_{\varepsilon }\right)D\left(r\right)$ with $-1\leq \chi_{\varepsilon }\leq 0$, eventually leads to $E\left(r\right)=0$ when $\chi_{\varepsilon }=-1$. This is directly analogous to the M-H, $\chi_{m}$, scheme of diamagnetic materials where $M\left(r\right)=\chi_{m}H(r)$ and $B\left(r\right)=\mu_{0}\left(H\left(r\right)+M\left(r\right)\right)=\mu_{0}\left(H\left(r\right)+\chi_{m}H\left(r\right)\right)=\mu_{0}\left(1+\chi_{m}\right)H\left(r\right)$ with $-1\leq \chi_{m}\leq 0$, that enables $B\left(r\right)=0$ when $\chi_{m}=-1$ [26].

Due to these facts, analogously to the LHI diamagnetic materials, on the basis of the P-D, $\chi_{\varepsilon }$, formulation, the LHI dielectric materials could be termed ‘diaelectric’ (di(a)electric).

## 5. Technical Considerations on the Calculation of D(r)

From a technical point of view, the introduction of the P-D electric susceptibility, $\chi_{\varepsilon }$, with $-1\leq \chi_{\varepsilon }\leq 0$ is meaningful only if D(r) can always be calculated beforehand (that is independently of E(r)) so that, in turn, $\tilde{P}\left(r\right)=-P(r)$ can be derived through relation (17), while E(r) can be obtained through relation (18). Once this is proved, D(r) can rightfully be called *primary* vector field, while P(r) and E(r) can be termed *secondary* vector fields. To this effect, below, we provide means to calculate D(r) on a standalone basis in systems wherein a known *free* charge density, $\rho_{f}(r)$, coexists with LHI dielectrics of known P-D electric susceptibility, χ_ε_. Apparently, except for the methods discussed below, additional ones can also be employed, such as Green’s functions, method of images/inversion, etc., [8,9,10,11,12,13,14].

### 5.1. Direct Calculation of D(r) by Means of Standard Integrals and Differential Equations

Standard methods of integral and differential equations can be used to obtain D(r), at least in relatively simple cases.

#### 5.1.1. Standard Integral

The law of Coulomb:

(36) $$D\left(r\right)=\frac{1}{4\pi }\int_{V^{/}}\frac{\rho_{f}\left(r^{/}\right)\left(r-r^{/}\right)}{{\left|r-r^{/}\right|}^{3}}dV^{/}\;$$

can be employed, together with the respective boundary conditions (see below). In relation (36), $V^{/}$ is the volume wherein the *free* charges exist, while $r^{/}$ is the respective position vector. Obviously, this strategy is advantageous for analytical calculations in relatively symmetric systems where D(r) has less than three components and depends on less than three variables.

#### 5.1.2. Standard Differential Equations

The Helmholtz theorem can be employed based on the divergence and curl of D(r) so that:

(37) $$\nabla \cdot D\left(r\right)=\rho_{f}\left(r\right)\;$$

(38) $$\mathrm{and}\;\nabla \times D\left(r\right)=0.$$

Solving consistently these differential equations, together with the respective boundary conditions (see below), will provide a unique $D\left(r\right)$. This strategy is practically equivalent to that of the above Section 5.1.1. Similarly, it is advantageous in relatively symmetric systems where D(r) has less than three components and depends on less than three variables.

### 5.2. Calculation of D(r) from a Free Scalar Potential, $U_{f}\left(r\right)$, and a Bound Vector Potential, $A_{b}\left(r\right)$

Based on the Helmholtz theorem [21,22,23,24], in the general case, we are able to express (or more accurately, we are obliged to express) the electric displacement, $D\left(r\right)$, by means of two functions, a scalar, $U_{f}\left(r\right)$, and a vector, $A_{b}\left(r\right)$ through the following relation:

(39) $$D\left(r\right)=-\varepsilon_{0}\nabla U_{f}\left(r\right)+\nabla \times A_{b}\left(r\right)\;$$

where

(40) $$U_{f}\left(r\right)=\frac{1}{4\pi \varepsilon_{0}}\int_{V^{/}}\frac{\nabla^{/}\cdot D\left(r^{/}\right)}{\left|r-r^{/}\right|}dV^{/}+\frac{1}{4\pi \varepsilon_{0}}\oint_{S^{/}}\frac{{\hat{n}}^{/}\cdot \left(D_{out}\left(r^{/}\right)-D_{in}\left(r^{/}\right)\right)}{\left|r-r^{/}\right|}dS^{/}$$

and

(41) $$A_{b}\left(r\right)=\frac{1}{4\pi }\int_{V^{/}}\frac{\nabla^{/}\times D\left(r^{/}\right)}{\left|r-r^{/}\right|}dV^{/}+\frac{1}{4\pi }\oint_{S^{/}}\frac{{\hat{n}}^{/}\times \left(D_{out}\left(r^{/}\right)-D_{in}\left(r^{/}\right)\right)}{\left|r-r^{/}\right|}dS^{/}.$$

We call these functions the *free* scalar potential, $U_{f}\left(r\right)$, and the *bound* vector potential, $A_{b}\left(r\right)$, for reasons that will become clear below. In these relations, $V^{/}$ is the entire space that for the most representative case discussed here is divided in two subspaces, ‘in’ (first LHI dielectric medium) and ‘out’ (second LHI dielectric medium), by an *interface* $S^{/}$. Also, ${\hat{n}}^{/}$ is the unit vector, normal to $S^{/}$, pointing from subspace ‘in’ to subspace ‘out’. Obviously, the divergence, $\nabla^{/}\cdot D\left(r^{/}\right)$, and the curl, $\nabla^{/}\times D\left(r^{/}\right)$, should be known since they are the volume sources of $U_{f}\left(r\right)$ and $A_{b}\left(r\right)$, respectively, that reside at the *interior* of LHI dielectrics. The second term in the numerator of each integral can be considered as the surface sources of $U_{f}\left(r\right)$ and $A_{b}\left(r\right)$ or, more conveniently, as the boundary conditions that should be satisfied by $D\left(r\right)$ at the *interface* $S^{/}$. For details, see Appendix A.1 of the Appendix A.

For the case of electrostatics discussed here in LHI dielectrics [8,9,10,11,12,13,14], at an *interface* $S^{/}$, two fundamental boundary conditions should hold for $E\left(r\right)$ that, in turn, impose restrictions on $\tilde{P}\left(r\right)=-P\left(r\right)$ and $D\left(r\right)$. Below, we express these boundary conditions in respect to $D\left(r\right)$ since this is the *primary* vector field of interest in the P-D, $\chi_{\varepsilon }$, formulation. The first refers to the normal to $S^{/}$ component of $E\left(r\right)$:

(42) $${\hat{n}}^{/}\cdot \left(E_{out}\left(r^{/}\right)-E_{in}\left(r^{/}\right)\right)\;{|}_{S^{/}}=\sigma \left(r^{/}\right){|}_{S^{/}}/\varepsilon_{0}$$

that translates into the two relations:

(43) $${\hat{n}}^{/}\cdot \left(D_{out}\left(r^{/}\right)-D_{in}\left(r^{/}\right)\right){|}_{S^{/}}=\sigma_{f}\left(r^{/}\right){|}_{S^{/}}\;$$

and

(44) $${\hat{n}}^{/}\cdot \left(\chi_{\varepsilon ,out}D_{out}\left(r^{/}\right)-\chi_{\varepsilon ,in}D_{in}\left(r^{/}\right)\right){|}_{S^{/}}=\sigma_{b}\left(r^{/}\right){|}_{S^{/}}\;.$$

The second relates to the tangential to $S^{/}$ components of $E\left(r\right)$:

(45) $${\hat{n}}^{/}\times \left(E_{out}\left(r^{/}\right)-E_{in}\left(r^{/}\right)\right){|}_{S^{/}}=0$$

and simply transforms to:

(46) $${\hat{n}}^{/}\times \left(D_{out}\left(r^{/}\right)-D_{in}\left(r^{/}\right)\right){|}_{S^{/}}=\left({\tilde{P}}_{out}\left(r^{/}\right)-{\tilde{P}}_{in}\left(r^{/}\right)\right)\times {\hat{n}}^{/}{|}_{S^{/}}\;$$

else

(47) $$(1+\chi_{\varepsilon ,out}){\hat{n}}^{/}\times D_{out}\left(r\right){|}_{S^{/}}=(1+\chi_{\varepsilon ,in}){\hat{n}}^{/}\times D_{in}\left(r\right){|}_{S^{/}}\;.$$

In the above, recall that the *bound* surface charge density is given by $\sigma_{b}\left(r^{/}\right){|}_{S^{/}}={\hat{n}}^{/}\cdot P(r^{/}){|}_{S^{/}}$, where the normal to $S^{/}$, unit vector ${\hat{n}}^{/}$ points outwards from the medium wherein $P(r^{/})$ resides [8,9,10,11,12,13,14], while $\tilde{P}\left(r^{/}\right)=-P(r^{/})$. For details on the foreground mathematics and the underlying physics of the boundary conditions, see Appendix A.1 of the Appendix A.

Now, we are able to specify the physical identity of $U_{f}\left(r\right)$ and $A_{b}\left(r\right)$ for the general case of any dielectric (not necessarily LHI). By means of relations (23) and (43), we see that the scalar function, $U_{f}\left(r\right)$, given by relation (40), is actually an electric potential that relates solely on *free* charges. Thus, we term it *free* scalar potential, $U_{f}\left(r\right)$. In analogy, through relations (30) and (46), we see that the vector function, $A_{b}\left(r\right)$, given by relation (41), reminisces of a magnetic potential that can be expressed exclusively by the properties of the dielectric. Thus, we call it *bound* vector potential, $A_{b}\left(r\right)$.

Most importantly, in the general case of any dielectric (not necessarily LHI) under the premise that the boundary conditions of relations (43) and (46) are fulfilled, relations (40) and (41) get the comparatively simple form:

(48) $$U_{f}\left(r\right)=\frac{1}{4\pi \varepsilon_{0}}\int_{V^{/}}\frac{\nabla^{/}\cdot D\left(r^{/}\right)}{\left|r-r^{/}\right|}dV^{/}\;$$

and

(49) $$A_{b}\left(r\right)=\frac{1}{4\pi }\int_{V^{/}}\frac{\nabla^{/}\times D\left(r^{/}\right)}{\left|r-r^{/}\right|}dV^{/}\;$$

where the volume sources $\nabla^{/}\cdot D\left(r^{/}\right)$ and $\nabla^{/}\times D\left(r^{/}\right)$ are ascribed to $\rho_{f}\left(r^{/}\right)$ (relation (23)) and $\nabla \times (-\tilde{P}\left(r\right))$ (relation (30)), respectively.

Returning back to the LHI dielectrics discussed here [8,9,10,11,12,13,14], at the *interior* of each medium, relations (23) and (31) now hold, thus $\nabla^{/}\cdot D\left(r^{/}\right)=\rho_{f}\left(r^{/}\right)$ if a non-zero $\rho_{f}\left(r^{/}\right)$ exists, while $\nabla^{/}\times D\left(r^{/}\right)=0$ in all circumstances. Accordingly, in LHI dielectrics, the *free* scalar potential, $U_{f}\left(r\right)$, of relation (48) evolves to the familiar form:

(50) $$U_{f}\left(r\right)=\frac{1}{4\pi \varepsilon_{0}}\int_{V^{/}}\frac{\rho_{f}\left(r^{/}\right)}{\left|r-r^{/}\right|}dV^{/}\;,$$

while the *bound* vector potential, $A_{b}\left(r\right)$, of relation (49) gets:

(51) $$A_{b}\left(r\right)=0\;.$$

By using the above relations (50)–(51), we conclude that for the LHI dielectrics discussed here, the electric displacement, $D\left(r\right)$, is given by the following simple version of relation (39) [27]:

(52) $$D\left(r\right)=-\varepsilon_{0}\nabla U_{f}\left(r\right)\;$$

under the premise that it satisfies the boundary conditions of relations (43)–(47).

However, we still have to define the boundary conditions that the *free* scalar potential, $U_{f}\left(r\right)$, should obey at interfaces of distinct LHI dielectrics. These should accompany the solution obtained either through relation (50) or by any of the strategies outlined below. To this end, given that $D\left(r\right)$ should obey the boundary conditions of relations (43) and (47), by using relation (52) we are now able to express them in respect to $U_{f}\left(r\right)$, instead of $D\left(r\right)$. We easily get:

(53) $$\left({\left(\nabla U_{f,out}\left(r\right)\right)}^{⊥}-{\left(\nabla U_{f,in}\left(r\right)\right)}^{⊥}\right){|}_{S^{/}}=-\sigma_{f}(r){|}_{S^{/}}/\varepsilon_{0}\;$$

and

(54) $$(1+\chi_{\varepsilon ,out}){\left(\nabla U_{f,out}\left(r\right)\right)}^{//}{|}_{S^{/}}=(1+\chi_{\varepsilon ,in}){\left(\nabla U_{f,in}\left(r\right)\right)}^{//}{|}_{S^{/}}\;,$$

where ${\left(\nabla U_{f}\left(r\right)\right)}^{⊥}=\hat{n}\cdot (\nabla U_{f}\left(r\right))=-D^{⊥}\left(r\right)/\varepsilon_{0}=-\hat{n}\cdot D\left(r\right)/\varepsilon_{0}$ refers to the *normal* and ${\left(\nabla U_{f}\left(r\right)\right)}^{//}=\hat{n}\times (\nabla U_{f}\left(r\right))=-D^{//}\left(r\right)/\varepsilon_{0}=-\hat{n}\times D\left(r\right)/\varepsilon_{0}$ refers to the *tangential* components. Finally, we note that while the E-related scalar potential, $U\left(r\right)$, is continuous everywhere in space, the continuous behavior of the D-related scalar potential, $U_{f}\left(r\right)$, is guaranteed only at the *interior* of LHI dielectrics. At the *interfaces,* $U_{f}\left(r\right)$ may be *non* continuous. In close connection, while the electric field, $E\left(r\right)=-\nabla U\left(r\right)$, is irrotational everywhere in space, the irrotational character of the electric displacement, $D\left(r\right)=-\varepsilon_{0}\nabla U_{f}\left(r\right)$, is ensured only at the *interior* of LHI dielectrics. At the *interfaces,* $D\left(r\right)$ may be *non* irrotational. This is why in the boundary conditions discussed above, we did not refer to the continuity of $U_{f}\left(r\right)$ at $S^{/}$. These important issues are discussed in the next Section 5.3 and in Appendix A.1 of the Appendix A, as well.

Up to now we have clarified the constitutive relation between $D\left(r\right)$ and $U_{f}\left(r\right)$ and surveyed the boundary conditions that they should obey. Still, we have to recruit methods to calculate the *free* scalar potential, $U_{f}\left(r\right)$, through which we can obtain the electric displacement, $D\left(r\right)$, by using relation (52).

#### 5.2.1. Standard Integral

The law of Coulomb, relation (50), reproduced here for convenience:

(55) $$U_{f}\left(r\right)=\frac{1}{4\pi \varepsilon_{0}}\int_{V^{/}}\frac{\rho_{f}\left(r^{/}\right)}{\left|r-r^{/}\right|}dV^{/}$$

can be employed together with the respective boundary conditions summarized above in relations (53)–(54). Obviously, this strategy is advantageous for analytical calculations in relatively symmetric systems.

#### 5.2.2. Poisson and Laplace Equations

The introduction of a *free* scalar potential, $U_{f}\left(r\right)$, that relates to $D\left(r\right)$ through relation (52), accompanied by relation (23), results in a Poisson and a Laplace equation, depending on the existence of $\rho_{f}\left(r\right)$ or not:

(56) $$\nabla^{2}U_{f}\left(r\right)=-\rho_{f}\left(r\right)/\varepsilon_{0}$$

(57) $$\mathrm{and}\;\nabla^{2}U_{f}\left(r\right)=0.$$

These differential equations can be recruited to compute $U_{f}\left(r\right)$, by means of standard techniques, such as the separation of variables for the Laplace equation [8,9,11,12,13,14]. For instance, for the latter case, in spherical coordinates, we have the following general solution:

(58) $$U_{f}\left(r\right)=\sum_{l=0}^{\infty }\sum_{m=-l}^{l}\left(A_{lm}r^{l}+B_{lm}r^{-(l+1)})Y_{lm}(\theta ,\varphi \right)\;$$

where $Y_{lm}(\theta ,\varphi )$ are the spherical harmonics. We stress that the obtained $U_{f}\left(r\right)$ should confront with the respective boundary conditions summarized above in relations (53)–(54). Once these boundary conditions have been taken into account, $D\left(r\right)$ can be obtained from $U_{f}\left(r\right)$ through relation (52).

#### 5.2.3. Multipole Expansion

The introduction of a *free* scalar potential, $U_{f}\left(r\right)$, that relates to $D\left(r\right)$ through relation (52) enables us to employ a multipole expansion to commit direct calculations [8,11,13,14]. For instance, in spherical coordinates, we have the following general expansions for $r^{/}<r$ (outside space: out) and for $r<r^{/}$ (inside space: in):

(59) $$U_{f}^{out}\left(r\right)=\frac{1}{4\pi \varepsilon_{0}}\sum_{l=0}^{\infty }\frac{4\pi }{2l+1}\frac{1}{r^{l+1}}\sum_{m=-l}^{l}q_{lm}^{out}Y_{lm}(\theta ,\varphi )\;$$

with

(60) $$q_{lm}^{out}=\int_{V^{/}}\rho_{f}\left(r^{/}\right){r^{/}}^{l}Y_{lm}^{*}(\theta^{/},\varphi^{/})dV^{/}\;$$

and

(61) $$U_{f}^{in}\left(r\right)=\frac{1}{4\pi \varepsilon_{0}}\sum_{l=0}^{\infty }\frac{4\pi }{2l+1}r^{l}\sum_{m=-l}^{l}q_{lm}^{in}Y_{lm}(\theta ,\varphi )\;$$

with

(62) $$q_{lm}^{in}=\int_{V^{/}}\rho_{f}\left(r^{/}\right)\frac{1}{{r^{/}}^{l+1}}Y_{lm}^{*}(\theta^{/},\varphi^{/})dV^{/}\;$$

where $q_{lm}^{out}$ and $q_{lm}^{in}$ are the so-called multipole moments for the outside and inside space, respectively. We stress that the obtained $U_{f}\left(r\right)$ should confront with the respective boundary conditions summarized above in relations (53)–(54). Once these boundary conditions have been taken into account, $D\left(r\right)$ can be obtained from $U_{f}\left(r\right)$ through relation (52).

### 5.3. $D\left(r\right)$ and $U_{f}\left(r\right):\;$ Dependence on the Dielectric Properties, Irrotationality and Continuity

Irrespectively of which strategy of the above we will employ to calculate $D\left(r\right)$ and $U_{f}\left(r\right)$, we stress that in the general case, they cannot depend exclusively on the *free* charges. Under specific circumstances, the properties of the LHI dielectrics can be imprinted onto $D\left(r\right)$ and $U_{f}\left(r\right)$ through the relevant boundary conditions discussed above, that should be satisfied at the *interfaces* of discontinuous dielectric media. Closely related to this issue are the irrotationality and the continuity of $D\left(r\right)$ and $U_{f}\left(r\right)$, respectively, at the *interior* and at the *interfaces* of LHI dielectrics. These issues are discussed in detail in Appendix A.1 of the Appendix A.

Here, we clarify briefly these issues that relate both conceptually to the physics and technically to the mathematics of the systems under investigation: First, the conditions under which $D\left(r\right)$ depends not only on the *free* charges but also on the properties of the LHI dielectrics. Second, the origin of a probably *non* irrotational character of $D\left(r\right)$. Third, the cause of a possibly *non* continuous behavior of $U_{f}\left(r\right)$.

First, from relation (41) we see that when $D\left(r\right)$ and $\tilde{P}\left(r\right)=-P(r)$ are exclusively *normal* to the interface $S^{/}$ of the LHI dielectrics, the *bound* vector potential, $A_{b}\left(r\right)$, does not exert any influence on both $D\left(r\right)$ and $U_{f}\left(r\right)$; neither through the volume term since at the *interior* of the LHI dielectrics $\nabla \times D\left(r\right)=0$ (recall relation (31)), nor through the surface term since at the *interfaces* of distinct LHI dielectrics, the respective boundary condition is trivially satisfied (recall relation (46)). Accordingly, when $D\left(r\right)$ and $\tilde{P}\left(r\right)=-P(r)$ are exclusively *normal* to the *interface* $S^{/}$ of the LHI dielectrics, the algebraic expressions of $D\left(r\right)$ and $U_{f}\left(r\right)$ should depend solely on *free* charges, relations (43), (50), (52) and (53), and should not contain any information that relate to the dielectric properties of the LHI materials. On the contrary, when $D\left(r\right)$ and $\tilde{P}\left(r\right)=-P(r)$ have at least one component *tangential* to the interface $S^{/}$ of the LHI dielectrics, $D\left(r\right)$ and $U_{f}\left(r\right)$ will depend on both the *free* charges and the dielectric properties of the LHI media (e.g., the P-D electric susceptibility, $\chi_{\varepsilon }$) since the respective boundary conditions, relations (47) and (54), inevitably introduce $\chi_{\varepsilon }$ in the algebra. Details can be found below and in Appendix A.1 of the Appendix A.

Second, while the electric displacement, $D\left(r\right)$, is obviously irrotational at the *interior* of all LHI dielectrics, it is *not* necessarily irrotational at the *interface* $S^{/}$ of distinct dielectric media having different properties (e.g., different P-D electric susceptibility, $\chi_{\varepsilon }$). Specifically, at an *interface* $S^{/}$, $D\left(r\right)$ should obey the boundary conditions of relation (43) (normal component, $D^{⊥}\left(r\right)$) and relations (46)–(47) (tangential components, $D^{//}\left(r\right)$). The latter inevitably imprints on $D\left(r\right)$ the behavior of $\tilde{P}\left(r\right)=-P(r)$. Thus, when $D\left(r\right)$ and $\tilde{P}\left(r\right)=-P(r)$ have components *tangential* to *interface* $S^{/}$, $D\left(r\right)$ will attain a *non* irrotational behavior at $S^{/}$ due to the inherent discontinuity of the electric polarization, $\tilde{P}\left(r\right)=-P(r)$, that we apparently experience at the interface $S^{/}$ as we move between two different dielectric media. On the contrary, when $D\left(r\right)$ and $\tilde{P}\left(r\right)=-P(r)$ are exclusively *normal* to the interface $S^{/}$, the boundary conditions of relations (46)–(47) are trivially satisfied so that $D\left(r\right)$ obtains an irrotational behavior at $S^{/}$. Details can be found in Appendix A.1 of the Appendix A.

Third, the discussion on the continuity of the *free* scalar potential, $U_{f}\left(r\right)$, at an *interface* $S^{/}$, follows closely the one made above on the irrotational behavior of $D\left(r\right)$, since these two entities couple through relation (52). While the *free* scalar potential, $U_{f}\left(r\right)$, is obviously continuous at the *interior* of all LHI dielectrics, it is *not* necessarily continuous at the *interface* $S^{/}$ of distinct dielectric media having different properties (e.g., different P-D electric susceptibility, $\chi_{\varepsilon }$). Specifically, it comes out that when $D\left(r\right)$ and $\tilde{P}\left(r\right)=-P(r)$ have components *tangential* to interface $S^{/}$, the *non* irrotational behavior of $D\left(r\right)$ will dictate a *non* continuous character onto $U_{f}\left(r\right)$ at $S^{/}$. On the contrary, when $D\left(r\right)$ and $\tilde{P}\left(r\right)=-P(r)$ are exclusively *normal* to the interface $S^{/}$, the irrotational behavior of $D\left(r\right)$ will enable $U_{f}\left(r\right)$ to preserve a continuous character at $S^{/}$. Details can be found in Appendix A.1 of the Appendix A.

To conclude, when $D\left(r\right)$ and $\tilde{P}\left(r\right)=-P(r)$ are exclusively *normal* to the *interfaces* of distinct LHI dielectric media, $D\left(r\right)$ is irrotational and $U_{f}\left(r\right)$ is continuous, not only at the *interior* of these LHI dielectric media but also at their *interfaces*. Then, the expressions of $D\left(r\right)$ and $U_{f}\left(r\right)$ should depend solely on *free* charges. On the contrary, when $D\left(r\right)$ and $\tilde{P}\left(r\right)=-P(r)$ have at least one component *tangential* to any *interface*, while still $D\left(r\right)$ is irrotational and $U_{f}\left(r\right)$ is continuous at the *interior* of the dielectrics, they are *non* irrotational and *non* continuous, respectively, at the relevant interfaces. Now, $D\left(r\right)$ and $U_{f}\left(r\right)$ depend on both the *free* charges and the properties of the LHI dielectrics (e.g., the P-D electric susceptibility, $\chi_{\varepsilon }$).

Here, we summarize the above information in a ready-to-use technical scheme for both $D\left(r\right)$ and $U_{f}\left(r\right)$: (i) Referring to the electric displacement, $D\left(r\right)$, it can be obtained directly through the standard integral and differential equations discussed in Section 5.1, together with the boundary conditions of relations (43) and (47). (ii) Regarding the *free* scalar potential, $U_{f}\left(r\right)$, it can be obtained directly through the standard integral, differential equation, and multipole expansion techniques discussed in Section 5.2, together with the boundary conditions of relations (53) and (54). In this case, $D\left(r\right)$ is finally obtained through relation (52).

Putting aside the above discussion based on mathematics, below we present a schematic argumentation, as a rule of thumb, for the most efficient calculation of the *primary* vector field, $D\left(r\right)$. Practically, whether $D\left(r\right)$ will depend solely on the *free* charges or it ultimately will be affected by the properties of the LHI dielectrics is determined, first, by the geometric morphology of both the *free* charges and LHI dielectrics and, second, by the relative orientation of their surfaces.

We recall that the system under investigation comprises of *free* charges and LHI dielectrics. Initially, consider only the *free* charges existing in the otherwise free space (the LHI dielectrics are removed temporarily) and calculate the relevant *external* electric displacement, $D_{ext}\left(r\right)$, based on the most convenient strategy of those reported above (we use the term *external* to stress the fact that this component originates solely from the *free* charges in the absence of the LHI dielectrics). Then, introduce the LHI dielectrics at their former positions and check the relative coordination between their interfaces and the ‘frozen’ $D_{ext}\left(r\right)$. If $D_{ext}\left(r\right)$ is exclusively *normal* to the interfaces of all LHI dielectrics, the final expression of the *total* electric displacement, $D\left(r\right)$, will not be affected by the properties of the LHI dielectrics. Thus, the *total* $D\left(r\right)$ should be identical to the *external* $D_{ext}\left(r\right)$, that is $D\left(r\right)=D_{ext}\left(r\right)$. On the contrary, if $D_{ext}\left(r\right)$ has at least one component *tangential* to any of the interfaces of the LHI dielectrics, apart from the original information on the *free* charges, the final expression of the *total* electric displacement, $D\left(r\right)$, will contain information on the properties of the LHI dielectrics. This will be realized mathematically by means of the boundary conditions that the *total* $D\left(r\right)$ should satisfy.

**Figure 1:**
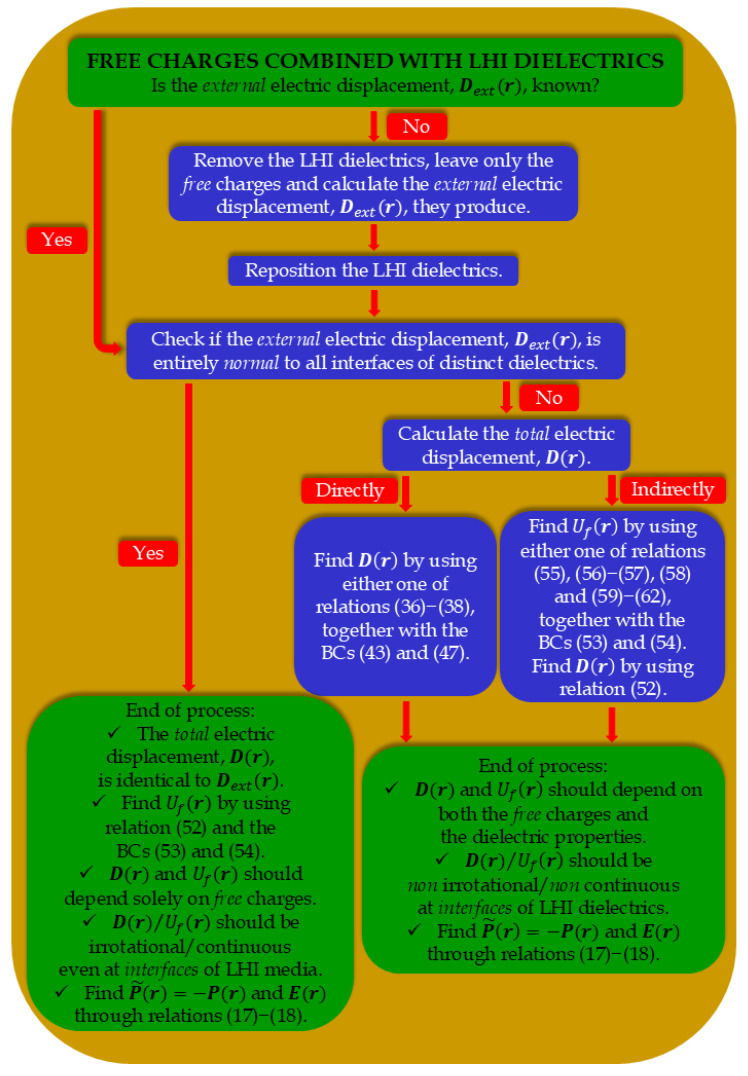
Ready-to-use work diagram for treating the electrostatic problem referring to a system of *free* charges combined with LHI dielectrics. $D_{ext}\left(r\right)$ refers to the *external* electric displacement that originates exclusively from the *free* charges, thus calculated in the absence of the LHI dielectrics. $D\left(r\right)$ refers to the *total* electric displacement, thus calculated in the presence of the LHI dielectrics. In general, $D\left(r\right)$ can depend on both the *free* charges and the dielectric properties. $U_{f}\left(r\right)$ is the *free* scalar potential that in general can depend on both the *free* charges and the dielectric properties, as well. BCs stands for boundary conditions.

Figure 1 summarizes the above considerations on $D\left(r\right)$ and $U_{f}\left(r\right)$ in a work diagram that is ready-to-use for the treatment of the general problem wherein *free* charges are combined with LHI dielectrics.

No matter whether the *total* $D\left(r\right)$ will depend exclusively on the *free* charges or on the properties of the dielectric media as well, here we have set the basis for the development of technical means to calculate it beforehand, that is independently of $\tilde{P}\left(r\right)=-P(r)$ and $E\left(r\right)$. This is important for the documentation of the so-called P-D, $\chi_{\varepsilon }$, formulation introduced in our work. The above discussion is tested against some representative problems of electrostatics. A basic example is presented below in Section 7. More are discussed in detail in Appendix A.2 of the Appendix A.

## 6. Importance of the Technical Means Reported on $D\left(r\right)$ and $U_{f}\left(r\right)$

The technical means discussed above on the calculation of $D\left(r\right)$ by the newly introduced *free* scalar potential, $U_{f}\left(r\right)$, can expand to include the *reverse* electric polarization $\tilde{P}\left(r\right)=-P\left(r\right)$, as well. Indeed, once at the *interior* of LHI dielectrics $\tilde{P}\left(r\right)$ is also irrotational, $\nabla \times \tilde{P}\left(r\right)=\nabla \times (-P\left(r\right))=0$, in analogy to $U_{f}\left(r\right)$ we can also define a *bound* scalar potential, $U_{b}\left(r\right)$, that originates from *bound* charges, through:

(63) $$\tilde{P}\left(r\right)=-P\left(r\right)=-\varepsilon_{0}\nabla U_{b}\left(r\right),$$

(64) $$\mathrm{else}\;P\left(r\right)=\varepsilon_{0}\nabla U_{b}\left(r\right).$$

In addition, since $\nabla \cdot \tilde{P}\left(r\right)=\nabla \cdot (-P\left(r\right))=\rho_{b}\left(r\right)$, we get the equation of Poisson and Laplace of $U_{b}\left(r\right)$ depending on the existence of $\rho_{b}\left(r\right)$ or not:

(65) $$\nabla^{2}U_{b}\left(r\right)=-\rho_{b}\left(r\right)/\varepsilon_{0}$$

(66) $$\mathrm{and}\;\nabla^{2}U_{b}\left(r\right)=0.$$

By recalling the respective relations (52), (56), and (57) of $D\left(r\right)$, it is easily seen that the superposition principle between the *free*, *bound,* and *total* scalar potentials ($U_{f}\left(r\right)$, $U_{b}\left(r\right)$, and $U\left(r\right)$, respectively), $U\left(r\right)=U_{f}\left(r\right)+U_{b}\left(r\right)$, complies nicely with the fundamental relations $D\left(r\right)=\varepsilon_{0}E\left(r\right)+P(r)$ and $\rho \left(r\right)=\rho_{f}\left(r\right)+\rho_{b}\left(r\right)$. Consequently, the methodology discussed above on the calculation of the *free* scalar potential, $U_{f}\left(r\right)$, can be employed to calculate the *bound* scalar potential, $U_{b}\left(r\right)$, as well.

The present technical propositions were originally triggered by the need to conceptually support the P-D electric susceptibility, $\chi_{\varepsilon }$. Nevertheless, we argue that the introduced *free* and *bound* scalar potentials, $U_{f}\left(r\right)$ and $U_{b}\left(r\right)$, respectively, are generic; they hold irrespectively of the specific constitutive relations that couple $E\left(r\right),\;D\left(r\right),$ and $P\left(r\right)$, given that these are linear and the proportionality coefficients do not depend on the position. These conditions are fulfilled for the LHI dielectrics discussed here. Thus, these relations can be used not only with the newly introduced P-D electric susceptibility, $\chi_{\varepsilon }$, expressed by relations (17)–(22). They can be employed with the standard formulation of the P-E electric susceptibility, $\chi_{e}$, expressed by relations (1)–(6), equally well. This expands the usability of the newly introduced *free*, $U_{f}\left(r\right)$, and *bound*, $U_{b}\left(r\right)$, scalar potentials, upgrading them to possibly important tools from both physical and mathematical aspects.

## 7. Consistency and Advancements of the New P-D, $\chi_{\varepsilon }$, Formulation

The new formulation, P-D, $\chi_{\varepsilon }$ (relations (17)–(22)) provides a different and clear viewpoint to the underlying physics of systems comprising of free charges and LHI dielectrics, in comparison to the standard one, P-E, $\chi_{e}$ (relations (1)–(6)). On quantitative terms, these two formulations should be absolutely consistent with each other. All physical entities such as scalar potentials, vector fields, $D\left(r\right)$, $P\left(r\right),$ and $E\left(r\right)$, dipole moments, p, *bound* charge densities, $\rho_{b}\left(r\right)$, etc., should attain the same form whether we employ the standard P-E, $\chi_{e}$, or the new P-D, $\chi_{\varepsilon }$, formulation. Nevertheless, the new formulation advances the understanding of the underlying physics since, among other, it successfully restores the mathematical and conceptual flaws which are inherent in the standard formulation. Here we document these issues through detailed results on some representative problems met in applications (see right below a basic example; more cases are considered in Appendix A.2 of the Appendix A).

The case of a dielectric sphere placed in an externally applied, uniform electric field is considered right below since this is a popular building element employed in many applications, such as scattering, invisibility cloaks, etc., [28,29,30,31,32]. In [28] (pages 136–141) and [29] (pages 74–79), the case of a LHI dielectric sphere with permittivity ε_1_ embedded in a LHI dielectric host medium of permittivity ε_m_ was considered. The system was subjected to an external, uniform electric field. This system is analytically considered quite usually since it is extendedly met in relevant scattering problems of electromagnetic radiation by subwavelength spherical particles; the effective dipole moment and polarizability which describe the complete system (sphere and host medium) are introduced in various formulas of subwavelength scattering such as scattering matrix elements and cross sections [28].

Thus, right below we consider a very relevant problem. A dielectric LHI sphere of P-E/P-D electric susceptibility $\chi_{e}/\chi_{\varepsilon }$ and radius a is placed with its center at the origin of the spherical coordinate system and is subjected to an *external*, uniform electric field directed along the *z*-axis, $E\left(r\right)=E_{0}\hat{z}$. The quantitative consistency between the two formulations P-E, $\chi_{e},$ and P-D, $\chi_{\varepsilon }$, is evidenced in Section 7.1 and Section 7.2. More importantly, a fundamental mathematical and conceptual flaw of the standard formulation, P-E, $\chi_{e},$ and its restoration by the new one, P-D, $\chi_{\varepsilon }$, is documented in Section 7.3.

### 7.1. Solution Based on the P-E Electric Susceptibility, $\chi_{e}$

The standard formulation based on the P-E electric susceptibility, $\chi_{e}$, focuses on the electric field, $E\left(r\right)$, and the respective scalar potential, $U\left(r\right)$, that relate through $E\left(r\right)=-\nabla U\left(r\right)$. Our calculations will be exclusively focused on $E\left(r\right)$ and $U\left(r\right)$, as well. Accordingly, we employ the solution of Laplace equation for the (obviously, φ-independent) scalar potential, $U\left(r\right)$, obtained through the method of separation of variables. For the outside space, a≤r, the solution has the form $U^{out}\left(r\right)=\sum_{l=0}^{\infty }\left(A_{l}^{out}r^{l}+B_{l}^{out}r^{-\left(l+1\right)}\right)P_{l}\left(cos\theta \right)$, while for the inside space, r≤a, reads $U^{in}\left(r\right)=\sum_{l=0}^{\infty }\left(A_{l}^{in}r^{l}+B_{l}^{in}r^{-\left(l+1\right)}\right)P_{l}\left(cos\theta \right)$. The following boundary conditions should hold on $U\left(r\right)$ and $E\left(r\right)$, for the inside (r≤a) and outside (a≤r) spaces, as well as at the interface (r=a) of the two dielectrics (sphere and vacuum): (i) for r = 0, $U^{in}\left(r\right)$ should be finite; (ii) for r →∞, $U^{out}\left(r\right)$ should be identical to the *external* scalar potential that produces $E\left(r\right)=E_{0}\hat{z}$, that is $U^{out}\left(r\right){|}_{r\to \infty }=-E_{0}rcos\theta$; (iii) $U\left(r\right)$ should be continuous at the interface of the two dielectric media, thus $U^{in}\left(r\right){|}_{r=a}=U^{out}\left(r\right){|}_{r=a}$; and (iv) the normal component of $E\left(r\right)$ should satisfy the relation $\left(E_{out}^{⊥}\left(r\right)-E_{in}^{⊥}\left(r\right)\right){|}_{r=a}=\sigma \left(r\right){|}_{r=a}/\varepsilon_{0}$ where since $\sigma_{f}\left(r\right){|}_{r=a}=0$ it translates to $\left(E_{out}^{⊥}\left(r\right)-E_{in}^{⊥}\left(r\right)\right){|}_{r=a}=\sigma_{b}\left(r\right){|}_{r=a}/\varepsilon_{0}$. Finally, notice that the extra boundary condition on the continuity of the tangential components of $E\left(r\right)$ (that is $E_{out}^{//}\left(r\right){|}_{r=a}=E_{in}^{//}\left(r\right){|}_{r=a}$), reproduces the continuity of $U\left(r\right)$ (condition (iii) above), thus, it does not add new information. Below, we briefly proceed with the solution.

Boundary condition (i) gives $B_{l}^{in}=0$ for 0 ≤ *l* < ∞, thus for the inside space the solution gets $U^{in}\left(r\right)=\sum_{l=0}^{\infty }A_{l}^{in}r^{l}P_{l}\left(cos\theta \right)$.
Boundary condition (ii) gives $A_{l}^{out}=0$ for 0 ≤ *l* < ∞, l≠1, thus for the outside space the solution becomes $U^{out}\left(r\right)=-E_{0}rcos\theta +\sum_{l=0}^{\infty }B_{l}^{out}r^{-\left(l+1\right)}P_{l}\left(cos\theta \right)$.
Boundary condition (iii) gives $A_{l}^{in}a^{l}=B_{l}^{out}a^{-\left(l+1\right)}$ for 0 ≤ *l* < ∞, l≠1 and $A_{1}^{in}=-E_{0}+B_{1}^{out}a^{-3}$ for *l* = 1.
Boundary condition (iv) gives $\left(E_{out}^{⊥}\left(r\right)-E_{in}^{⊥}\left(r\right)\right){|}_{r=a}=\sigma_{b}\left(r\right){|}_{r=a}/\varepsilon_{0}$ that since $\sigma_{b}\left(r\right){|}_{r=a}=\hat{r}\cdot P_{in}\left(r\right){|}_{r=a}=\hat{r}\cdot \left(\chi_{e}\varepsilon_{0}E_{in}\left(r\right)\right){|}_{r=a}=\chi_{e}\varepsilon_{0}E_{in}^{⊥}\left(r\right){|}_{r=a}$ it transforms to $E_{out}^{⊥}\left(r\right){|}_{r=a}=\left(1+\chi_{e}\right)E_{in}^{⊥}\left(r\right){|}_{r=a}$, else $E_{out}^{⊥}\left(r\right){|}_{r=a}=\varepsilon_{r}E_{in}^{⊥}\left(r\right){|}_{r=a}$. The latter ultimately provides the desired relation $–(\partial U^{out}\left(r\right)/\partial r){|}_{r=a}=\varepsilon_{r}(-(\partial U^{in}\left(r\right)/\partial r)){|}_{r=a}$ that gives $-B_{l}^{out}\left(l+1\right)a^{-\left(l+2\right)}=\varepsilon_{r}A_{l}^{in}la^{l-1}$ for l≠1 and $-E_{0}-B_{1}^{out}2a^{-3}=\varepsilon_{r}A_{1}^{in}$ for l=1.

The above set of relations results in $U^{out}\left(r\right)=-[1-((\varepsilon_{r}-1)/(\varepsilon_{r}+2))({a/r)}^{3}]E_{0}rcos\theta$ (outside space) and $U^{in}\left(r\right)=-(3/(\varepsilon_{r}+2))E_{0}rcos\theta =-(3/(\varepsilon_{r}+2))E_{0}z$ (inside space), for the total scalar potential.

Through $E\left(r\right)=-\nabla U\left(r\right)$ we get the respective relations for the electric field $E^{out}\left(r\right)=E_{0}\hat{z}+\left(1/4\pi \varepsilon_{0}\right)\left(1/r^{3}\right)\left(3\left(p\cdot \hat{r}\right)\hat{r}-p\right)$, where $p=3\varepsilon_{0}((\varepsilon_{r}-1)/(\varepsilon_{r}+2))V_{sp}E_{0}\hat{z}$ and $V_{sp}=(4/3)\pi a^{3}$, and $E^{in}\left(r\right)=(3/(\varepsilon_{r}+2))E_{0}\hat{z}.$

Once we have found $E\left(r\right)$, we easily calculate the respective electric polarization, $P\left(r\right)$, and electric displacement, $D\left(r\right)$, since they relate to $E\left(r\right)$ through expressions (1) and (2) of the article. Thus, we get $P^{out}\left(r\right)=0$ and $P^{in}\left(r\right)=3\varepsilon_{0}(\chi_{e}/(\varepsilon_{r}+2))E_{0}\hat{z}$ for the electric polarization, while $D^{out}\left(r\right)=\varepsilon_{0}E^{out}\left(r\right)=D_{0}\hat{z}+\left(1/4\pi \right)\left(1/r^{3}\right)\left(3\left(p\cdot \hat{r}\right)\hat{r}-p\right)$ and $D^{in}\left(r\right)=\varepsilon E^{in}\left(r\right)=3(\varepsilon_{r}/(\varepsilon_{r}+2))D_{0}\hat{z}$, where $\varepsilon_{0}E_{0}\hat{z}=D_{0}\hat{z}$ the *external* electric displacement.

*Depolarizing field/self field:* The surface density of the bound charge that resides at the interface r = a is $\sigma_{b}\left(r\right){|}_{r=a}=\hat{r}\cdot P^{in}\left(r\right){|}_{r=a}=\hat{r}\cdot \left(\chi_{e}\varepsilon_{0}{Ε}^{in}\left(r\right)\right){|}_{r=a}=3\varepsilon_{0}((\varepsilon_{r}-1)/(\varepsilon_{r}+2))E_{0}cos\theta$. The relevant *internal* electric field (see [25]) produced by $\sigma_{b}\left(r\right){|}_{r=a}$ at the inside space is simply $E_{int}^{in}\left(r\right)=E^{in}\left(r\right)-E_{ext}^{in}\left(r\right)=(3/(\varepsilon_{r}+2))E_{0}\hat{z}-E_{0}\hat{z}=-((\varepsilon_{r}-1)/(\varepsilon_{r}+2))E_{0}\hat{z}$. Also, we can easily obtain $E_{int}^{in}\left(r\right)=-(1/3\varepsilon_{0})P^{in}\left(r\right)$. We recall that $E_{int}^{in}\left(r\right)$ is the so-called depolarizing field, or self field, that relates to $P^{in}\left(r\right)$, else to the reverse ${\tilde{P}}^{in}\left(r\right)=-P^{in}\left(r\right)$ through $E_{int}^{in}\left(r\right)=(1/3\varepsilon_{0}){\tilde{P}}^{in}\left(r\right)$ (see Section 3 above and Section 7.2 below).

Dependence of ***D***(***r***) on free charges and dielectric properties: Here, the electric displacement that relates to the external sources, $D_{ext}\left(r\right)=D_{0}\hat{z}=\varepsilon_{0}E_{0}\hat{z}=\varepsilon_{0}E_{0}cos\theta \hat{r}-\varepsilon_{0}E_{0}sin\theta \hat{\theta }$, has component that is tangential to the surface of the dielectric sphere. Thus, we expect that the total $D\left(r\right)$, except for the free charges, should depend on the properties of the LHI dielectric sphere (see Section 5.2 and Section 5.3 above and Appendix A.1 of the Appendix A). Indeed, this is what we observe here.

### 7.2. Solution Based on the P-D Electric Susceptibility, $\chi_{\varepsilon }$

The alternative formulation based on the P-D electric susceptibility, $\chi_{\varepsilon }$, should focus on the electric displacement, $D\left(r\right)$, and the respective *free* scalar potential, $U_{f}\left(r\right)$, that relate through $D\left(r\right)=-\varepsilon_{0}\nabla U_{f}\left(r\right)$. Accordingly, we employ the solution of Laplace equation for the (obviously, φ-independent) *free* scalar potential, $U_{f}\left(r\right)$, obtained through the method of separation of variables. For the outside space, a≤r, the solution has the form $U_{f}^{out}\left(r\right)=\sum_{l=0}^{\infty }\left(A_{l}^{out}r^{l}+B_{l}^{out}r^{-\left(l+1\right)}\right)P_{l}\left(cos\theta \right)$, while for the inside space, r≤a, reads $U_{f}^{in}\left(r\right)=\sum_{l=0}^{\infty }\left(A_{l}^{in}r^{l}+B_{l}^{in}r^{-\left(l+1\right)}\right)P_{l}\left(cos\theta \right)$. The following boundary conditions should hold on $U_{f}\left(r\right)$ and $D\left(r\right)$, for the inside (r≤a) and outside (a≤r) spaces, as well as at the interface (r=a) of the two dielectrics (sphere and vacuum): (i) for r = 0, $U_{f}^{in}\left(r\right)$ should be finite; (ii) for r →∞, $U_{f}^{out}\left(r\right)$ should be identical to the *external* scalar potential that produces $D\left(r\right)=D_{0}\hat{z}$ ($D\left(r\right)=\varepsilon_{0}E_{0}\hat{z}$), that is $U_{f}^{out}\left(r\right){|}_{r\to \infty }=-E_{0}rcos\theta$; (iii) the tangential components of $D\left(r\right)$ should satisfy the relation $\left(D_{out}^{//}\left(r\right)-D_{in}^{//}\left(r\right)\right){|}_{r=a}=-\left({\tilde{P}}_{out}^{//}\left(r\right)-{\tilde{P}}_{in}^{//}\left(r\right)\right){|}_{r=a}$ that since $\tilde{P}\left(r\right)=-P\left(r\right)=\chi_{\varepsilon }D\left(r\right)$ and $\chi_{\varepsilon }^{out}=0,$ it gets $D_{out}^{//}\left(r\right){|}_{r=a}=(1+\chi_{\varepsilon }^{in})D_{in}^{//}\left(r\right){|}_{r=a}$; and (iv) the normal component of $D\left(r\right)$ should satisfy the condition $\left(D_{out}^{⊥}\left(r\right)-D_{in}^{⊥}\left(r\right)\right){|}_{r=a}=\sigma_{f}\left(r\right){|}_{r=a}$ where since $\sigma_{f}\left(r\right){|}_{r=a}=0$ it translates to $D_{out}^{⊥}\left(r\right){|}_{r=a}=D_{in}^{⊥}\left(r\right){|}_{r=a}$. Finally, notice that in contrast to the inherent continuity of $U\left(r\right)$, the *free* scalar potential, $U_{f}\left(r\right)$, is *not* necessarily continuous at the interface of two dielectric media (see Section 5.2 and Section 5.3 above and Appendix A.1 of the Appendix A). Thus, it is meaningless to ask for a boundary condition on the continuity of $U_{f}\left(r\right)$ at the interface r=a. As we will see below, indeed, the $U_{f}\left(r\right)$ is *non* continuous at the interface r=a. Next, we briefly proceed with the solution.

Boundary condition (i) gives $B_{l}^{in}=0$ for 0 ≤ *l* < ∞, thus for the inside space the solution gets $U^{in}\left(r\right)=\sum_{l=0}^{\infty }A_{l}^{in}r^{l}P_{l}\left(cos\theta \right)$.
Boundary condition (ii) gives $A_{l}^{out}=0$ for 0 ≤ *l* < ∞, l≠1, thus for the outside space the solution becomes $U^{out}\left(r\right)=-E_{0}rcos\theta +\sum_{l=0}^{\infty }B_{l}^{out}r^{-\left(l+1\right)}P_{l}\left(cos\theta \right)$.

To proceed with boundary conditions (iii) and (iv) we have to calculate the currently available version of $D\left(r\right)$ through $D\left(r\right)=-\varepsilon_{0}\nabla U_{f}\left(r\right).$ We get $D^{in}\left(r\right)=-\varepsilon_{0}\sum_{l=0}^{\infty }A_{l}^{in}lr^{l-1}P_{l}\left(cos\theta \right)\hat{r}-\varepsilon_{0}\sum_{l=0}^{\infty }A_{l}^{in}r^{l-1}(\partial P_{l}\left(cos\theta \right)/\partial \theta )\hat{\theta }$ and $D^{out}\left(r\right)=-\varepsilon_{0}\left(-E_{0}cos\theta +\sum_{l=0}^{\infty }B_{l}^{out}\left(-\left(l+1\right)\right)r^{-\left(l+2\right)}P_{l}\left(cos\theta \right)\right)\hat{r}-\varepsilon_{0}(-E_{0}(\partial P_{1}\left(cos\theta \right)/\partial \theta )+\sum_{l=0}^{\infty }B_{l}^{out}r^{-(l+2)}(\partial P_{l}\left(cos\theta \right)/\partial \theta ))\hat{\theta }$.

Boundary condition (iii) gives $B_{l}^{out}a^{-\left(l+2\right)}=(1+\chi_{\varepsilon })A_{l}^{in}a^{l-1}$ for 0 ≤ *l* < ∞, l≠1 and $-E_{0}+B_{1}^{out}a^{-3}=(1+\chi_{\varepsilon })A_{1}^{in}$ for *l* = 1.
Boundary condition (iv) gives $-B_{l}^{out}\left(l+1\right)a^{-\left(l+2\right)}=A_{l}^{in}la^{l-1}$ for l≠1 and $-E_{0}-2B_{1}^{out}a^{-3}=A_{1}^{in}$ for l=1.

The above set of relations results in $U_{f}^{out}\left(r\right)=-[1+(\chi_{\varepsilon }/(2\chi_{\varepsilon }+3))({a/r)}_{3}]E_{0}rcos\theta$ (outside space) and $U_{f}^{in}\left(r\right)=-(3/(2\chi_{\varepsilon }+3))E_{0}rcos\theta =-(3/(2\chi_{\varepsilon }+3))E_{0}z$ (inside space), for the *free* scalar potential.

Through $D\left(r\right)=-\varepsilon_{0}\nabla U_{f}\left(r\right)$ we get the desired expressions for the electric displacement, $D^{out}\left(r\right)=D_{0}\hat{z}+\left(1/4\pi \right)\left(1/r^{3}\right)\left(3\left(p_{\varepsilon }\cdot \hat{r}\right)\hat{r}-p_{\varepsilon }\right),$ where $p_{\varepsilon }=3(-\chi_{\varepsilon }/(2\chi_{\varepsilon }+3))V_{sp}D_{0}\hat{z}$ with $V_{sp}=(4/3)\pi a^{3}$ and $D_{0}=\varepsilon_{0}E_{0}$, and $D^{in}\left(r\right)=(3/(2\chi_{\varepsilon }+3))D_{0}\hat{z}.$ In the above relations, $p_{\varepsilon }$ is the electric dipole moment based on the P-D, $\chi_{\varepsilon }$, formulation, that since $-1\leq \chi_{\varepsilon }\leq 0$, it points to the positive z direction, as expected.

Once we have found $D\left(r\right)$, we easily calculate the respective *reverse* electric polarization, $\tilde{P}\left(r\right)$, (electric polarization, $P\left(r\right)$) and electric field, $E\left(r\right)$, since they relate to $D\left(r\right)$ through expressions (17) and (18). Thus, we get ${\tilde{P}}^{out}\left(r\right)=-P^{out}\left(r\right)=0$ and ${\tilde{P}}^{in}\left(r\right)=-P^{in}\left(r\right)=3(\chi_{\varepsilon }/(2\chi_{\varepsilon }+3))D_{0}\hat{z}$ for the *reverse* electric polarization, while $E^{out}\left(r\right)=D^{out}\left(r\right)/\varepsilon_{0}=E_{0}\hat{z}+\left(1/4\pi \varepsilon_{0}\right)\left(1/r^{3}\right)\left(3\left(p_{\varepsilon }\cdot \hat{r}\right)\hat{r}-p_{\varepsilon }\right)$ and $E^{in}\left(r\right)=D^{in}\left(r\right)/\varepsilon_{\varepsilon }=D^{in}\left(r\right)/(\varepsilon_{0}\varepsilon_{r\varepsilon })=D^{in}\left(r\right)/(\varepsilon_{0}{\left(1+\chi_{\varepsilon }\right)}^{-1})=(1+\chi_{\varepsilon })D^{in}\left(r\right)/\varepsilon_{0}=3((\chi_{\varepsilon }+1)/(2\chi_{\varepsilon }+3))E_{0}\hat{z}$ for the electric field.

*Depolarizing field/self field:* The surface density of bound charge that resides at the interface r=a is $\sigma_{b}\left(r\right){|}_{r=a}=\hat{r}\cdot P^{in}\left(r\right){|}_{r=a}=\hat{r}\cdot \left(-{\tilde{P}}^{in}\left(r\right)\right){|}_{r=a}=\hat{r}\cdot \left(-\chi_{\varepsilon }D^{in}\left(r\right)\right){|}_{r=a}=\hat{r}\cdot (-(3\chi_{\varepsilon }/(2\chi_{\varepsilon }+3))D_{0}\hat{z}){|}_{r=a}=-(3\chi_{\varepsilon }/(2\chi_{\varepsilon }+3))D_{0}cos\theta$, where $D_{0}=\varepsilon_{0}E_{0}$. The relevant *internal* electric field (see [25] of the article) produced by $\sigma_{b}\left(r\right){|}_{r=a}$ at the inside space is simply $E_{int}^{in}\left(r\right)=E^{in}\left(r\right)-E_{ext}^{in}\left(r\right)=3((\chi_{\varepsilon }+1)/(2\chi_{\varepsilon }+3))E_{0}\hat{z}-E_{0}\hat{z}=(\chi_{\varepsilon }/(2\chi_{\varepsilon }+3))E_{0}\hat{z}$. Also, we can easily obtain $E_{int}^{in}\left(r\right)=(1/3\varepsilon_{0}){\tilde{P}}^{in}\left(r\right)=-(1/3\varepsilon_{0})P^{in}\left(r\right)$. We recall that $E_{int}^{in}\left(r\right)$ is the so-called depolarizing field or self field (see Section 3 above).

Dependence of ***D***(***r***) on free charges and dielectric properties: As already discussed above for the P-E, $\chi_{e}$, formulation, the electric displacement that relates to the external sources, $D_{ext}\left(r\right)=D_{0}\hat{z}=\varepsilon_{0}E_{0}\hat{z}=\varepsilon_{0}E_{0}cos\theta \hat{r}-\varepsilon_{0}E_{0}sin\theta \hat{\theta },$ has component that is tangential to the surface of the dielectric sphere. Thus, we expect that the total $D\left(r\right)$, except for the free charges, should depend on the properties of the LHI dielectric sphere. This is expected even for the P-D, $\chi_{\varepsilon }$, formulation discussed here. Indeed, this is observed (see Section 5.2 and Section 5.3 above and Appendix A.1 of the Appendix A).

*Non-continuity of the free scalar potential, U_f_*(***r***)*, at the interface r = a:* The existence of a *tangential* component of $D\left(r\right)$ and $\tilde{P}\left(r\right)=-P\left(r\right)$ at the interface of different dielectric media will result in a *non* irrotational behavior of $D\left(r\right)$, and a *non* continuous character of $U_{f}\left(r\right)$, locally at the interface (see Section 5.2 and Section 5.3 above and Appendix A.1 of the Appendix A). Indeed, here $D\left(r\right)$ and $\tilde{P}\left(r\right)=-P\left(r\right)$ have a tangential component at the interface r = a. By using the expressions found above for $U_{f}^{out}\left(r\right)$ and $U_{f}^{in}\left(r\right)$, we see that $U_{f}^{out}\left(r\right){|}_{r=a}=-3((\chi_{\varepsilon }+1)/(2\chi_{\varepsilon }+3))E_{0}acos\theta$, while $U_{f}^{in}\left(r\right){|}_{r=a}=-3(1/(2\chi_{\varepsilon }+3))E_{0}acos\theta$. The respective discontinuity is $U_{f}^{out}\left(r\right){|}_{r=a}-U_{f}^{in}\left(r\right){|}_{r=a}=-3(\chi_{\varepsilon }/(2\chi_{\varepsilon }+3))E_{0}acos\theta$.

*Comparison between the P-E, $\chi_{e}$, and P-D, $\chi_{\varepsilon }$, formulations:* The two descriptions, the standard P-E, $\chi_{e}$, employed today and the alternative P-D, $\chi_{\varepsilon }$, introduced here, should be quantitatively consistent. Thus, all physical entities of electrostatics (scalar potentials, vector fields, dipole moments, bound charge densities, etc.) should be the same irrespectively of which description we use. To this effect, it is expected that when we substitute $\chi_{\varepsilon }=-\chi_{e}/(1+\chi_{e})$ (relation (34)) in the expressions obtained here in Section 7.2., we should get the exact same relations obtained above in Section 7.1. Indeed, this can be easily confirmed for all electric entities, displacement, $D\left(r\right)$, polarization, $P\left(r\right)$, field, $E\left(r\right)$, *free* scalar potential of the outside space (a≤r), $U_{f}^{out}\left(r\right)$, dipole moment, p, and *bound* surface charge density, $\sigma_{b}\left(r\right){|}_{r=a}$. In addition, we can easily verify that the relation $U\left(r\right)=U_{f}\left(r\right)+U_{b}\left(r\right)$ holds everywhere in space, where $U_{b}\left(r\right)$ is the *bound* scalar potential that relates to the *reverse* electric polarization, $\tilde{P}\left(r\right)=-P\left(r\right)$, through $\tilde{P}\left(r\right)=-\varepsilon_{0}\nabla U_{b}\left(r\right)$. For instance, at the outside space, a≤r, the relation $U^{out}\left(r\right)=U_{f}^{out}\left(r\right)$ holds, since $U_{b}^{out}\left(r\right)=0$. For the inside space, r≤a, we can easily find $U_{b}^{in}\left(r\right)$ and verify that, indeed, $U^{in}\left(r\right)=U_{f}^{in}\left(r\right)+U_{b}^{in}\left(r\right)$ (see Section 6 above).

### 7.3. Solution Based on the P-E Electric Susceptibility, $\chi_{e}$, and the P-D Electric Susceptibility, $\chi_{\varepsilon }$, by Means of Series

Here we analytically present the solution, for both formulations, by means of series. This approach is used to simulate mathematically the *“…awkward situation…”* described at page 68 in [8] *“…the polarization of the dielectric depends on the total electric field in the medium, but a part of the electric field is produced by the dielectric itself…”* and at page 186 in [13] *“…the external field will polarize the material, and this polarization will produce its own field, which then contributes to the total field, and this in turn modifies the polarization, which…Breaking out this infinite regress is not always easy”*. Accordingly, this approach considers the externally applied vector field ($E\left(r\right)$ for the standard formulation P-E, $\chi_{e}$ and $D\left(r\right)$ for the new one P-D, $\chi_{\varepsilon }$) as the first term of a series; this term induces an initial polarization to the specimen. In turn, the initial polarization produces a reaction field that adds to the external one. The new total field (superposition of the external and the reaction fields) induces an extra polarization to the specimen which produces an extra reaction field and so on. Each successive term of this iterative procedure is added to the first term (external field) so that ultimately the total field is just the sum of an infinite series. In this procedure a specific point should be carefully considered: in all cases the relevant convergence criterion has to be fulfilled. As we show below, while this is not formally assured for the standard formulation P-E, $\chi_{e}$, it is inherently guaranteed for the new one P-D, $\chi_{\varepsilon }$.

#### 7.3.1. P-E, $\chi_{e}$, Formulation

Here we employ a series-based approach with the standard formulation of the P-E electric susceptibility, $\chi_{e}$, and focus directly on the electric polarization, $P\left(r\right)$, and field, $E\left(r\right)$, to clarify their causality/feedback for the inside space, r≤a, of the LHI dielectric sphere.

Let us consider an experiment wherein we apply an *external* electric field $E_{0}\hat{z}$ to a specimen, trying to get information on its dielectric properties (susceptibility, $\chi_{e}$, relative permittivity, $\varepsilon_{r}$, etc.). We focus on the initial stage where the response of the dielectric to the *external* stimulus, $E_{0}\hat{z}$, still develops (transient state), that is before it eventually attains its permanent form (steady state). One could probably expect that the *external* electric field, $E_{0}\hat{z}$, applied by the user, would penetrate the specimen and polarize it both permanently and exclusively, in the sense that the following relation should hold, $P^{in\;}\left(r\right)=\chi_{e}\varepsilon_{0}E^{in}\left(r\right)=\chi_{e}\varepsilon_{0}E_{ext}^{in}\left(r\right)=\chi_{e}\varepsilon_{0}E_{0}\hat{z}$, where $E^{in}\left(r\right)$ is the total electric field inside the dielectric specimen. Indeed, this is what happens, however, *neither* permanently, *nor* exclusively. The above relation will hold only at the first moment of the ‘infinite regress of the P-E polarization process’. An additional electric field of *internal* origin, $E_{int}^{in}\left(r\right)$, will immediately appear, gradually evolve and eventually be established inside the dielectric specimen. $E_{int}^{in}\left(r\right)$ is produced by the polarization/*bound* charges of the specimen (see below) and adds to the *external* electric field $E_{ext}^{in}\left(r\right)=E_{0}\hat{z}$. Following this scheme, the *external* electric field can be considered as the zeroth-order term, $E_{0}^{in}\left(r\right)=E_{0}\hat{z}$, of the total electric field, $E^{in}\left(r\right)=E_{ext}^{in}\left(r\right)+E_{int}^{in}\left(r\right)$, that gradually develops (transient state) and eventually will be established (steady state) inside the dielectric specimen. Accordingly, the zeroth-order term, $E_{0}^{in}\left(r\right)=E_{0}\hat{z}$, will polarize, partially, the specimen. The respective zeroth-order term of the electric polarization, $P_{0}^{in}\left(r\right)$, induced by $E_{0}^{in}\left(r\right)$, is $P_{0}^{in}\left(r\right)=\chi_{e}\varepsilon_{0}E_{0}^{in}\left(r\right)=\chi_{e}\varepsilon_{0}E_{0}\hat{z}$. As we showed in both Section 7.1 and Section 7.2 above, a uniformly polarized sphere of polarization $P\left(r\right)$, relates to a *bound* surface charge density $\sigma_{b}\left(r\right){|}_{r=a}=\hat{r}\cdot P\left(r\right){|}_{r=a}$, that produces an *internal* electric field (depolarizing field/self field) $E_{int}^{in}\left(r\right)=-(1/3\varepsilon_{0})P^{in}\left(r\right)=(1/3\varepsilon_{0}){\tilde{P}}^{in}\left(r\right)$ at the inside space. Thus, the zeroth-order term of the polarization $P_{0}^{in}\left(r\right)$ will produce a first-order term for the *internal* electric field given by $E_{int,1}^{in}\left(r\right)=-(1/3\varepsilon_{0})P_{0}^{in}\left(r\right)$ (notice that the term $E_{int,0}^{in}\left(r\right)$ does not exist; the only zeroth-order electric field term is of *external* origin, $E_{0}^{in}\left(r\right)=E_{0}\hat{z}$). In turn, the first-order term, $E_{int,1}^{in}\left(r\right)$, will induce a first-order term for the polarization $P_{1}^{in}\left(r\right)=\chi_{e}\varepsilon_{0}E_{int,1}^{in}\left(r\right)$ that subsequently will produce a second-order term for the *internal* electric field $E_{int,2}^{in}\left(r\right)=-(1/3\varepsilon_{0})P_{1}^{in}\left(r\right)$, and so on. Thus, in general, the (i-1)-order term of the induced polarization is $P_{i-1}^{in}\left(r\right)=\chi_{e}\varepsilon_{0}E_{int,i-1}^{in}\left(r\right)$, while the (i)-order term of the *internal* electric field is $E_{int,i}^{in}\left(r\right)=-(1/3\varepsilon_{0})P_{i-1}^{in}\left(r\right)$. Combining these last two relations on $P_{i-1}^{in}\left(r\right)$ and $E_{int,i}^{in}\left(r\right),$ we get $E_{int,i}^{in}\left(r\right)=\left(-\left(\chi_{e}/3\right)\right)E_{int,i-1}^{in}\left(r\right)=\left(-\left(\chi_{e}/3\right)\right)\left(-\left(\chi_{e}/3\right)\right)E_{int,i-2}^{in}\left(r\right)=(-(\chi_{e}/3))(-(\chi_{e}/3))(-(\chi_{e}/3))E_{int,i-3}^{in}\left(r\right)=\cdots ={\left(-(\chi_{e}/3)\right)}^{i}E_{0}^{in}\left(r\right)$. Accordingly, the total electric field will simply be $E^{in}\left(r\right)=E_{0}^{in}\left(r\right)+E_{int,1}^{in}\left(r\right)+E_{int,2}^{in}\left(r\right)+\cdots +E_{int,i}^{in}\left(r\right)+\cdots =E_{0}^{in}\left(r\right)+(-(\chi_{e}/3))E_{0}^{in}\left(r\right)+(-(\chi_{e}/3))(-(\chi_{e}/3))E_{0}^{in}\left(r\right)+\cdots +{\left(-(\chi_{e}/3)\right)}^{i}E_{0}^{in}\left(r\right)+\cdots$, else $E^{in}\left(r\right)=\left(\sum_{i=0}^{\infty }{\left(-\left(\chi_{e}/3\right)\right)}^{i}\right)E_{0}^{in}\left(r\right).$ The geometric series results in $\sum_{i=0}^{\infty }{\left(-(\chi_{e}/3)\right)}^{i}=1/(1-(-(\chi_{e}/3)))=1/(1+\chi_{e}/3)$ so that ultimately $E^{in}\left(r\right)=E_{0}^{in}\left(r\right)/(1+\chi_{e}/3)$, else $E^{in}\left(r\right)=(3/(3+\chi_{e}))E_{0}\hat{z}$. This result is identical to the one obtained in Section 7.1 above, as expected.

This ‘infinite regress of the P-E polarization process’ applies, also, to the *bound* surface charge density, $\sigma_{b}\left(r\right){|}_{r=a}$, that gradually develops (transient state) and eventually will be established (steady state) at the interface, r=a, of the two dielectrics (sphere and vacuum). It should be noted that the *bound* charge density, $\sigma_{b}\left(r\right){|}_{r=a}$, which is induced by the primary source, the *free* charge density, acts as secondary source and ultimately produces inside the dielectric the *internal* electric field, $E_{int}^{in}\left(r\right)$. Specifically, the zeroth-order term of the polarization, $P_{0}^{in}\left(r\right)=\chi_{e}\varepsilon_{0}E_{0}^{in}\left(r\right)=\chi_{e}\varepsilon_{0}E_{ext}^{in}\left(r\right)=\chi_{e}\varepsilon_{0}E_{0}\hat{z}$, will induce a zeroth-order term in the *bound* surface charge density, $\sigma_{b,0}\left(r\right){|}_{r=a}=\hat{r}\cdot P_{0\;}^{in}\left(r\right){|}_{r=a}=\hat{r}\cdot (\chi_{e}\varepsilon_{0}E_{0}^{in}\left(r\right)){|}_{r=a}=\hat{r}\cdot \left(\chi_{e}\varepsilon_{0}E_{ext}^{in}\left(r\right)\right){|}_{r=a}=\chi_{e}\varepsilon_{0}E_{0}cos\theta$. Then, $\sigma_{b,0}\left(r\right){|}_{r=a}$ will produce a first-order term of the *internal* electric field, $E_{int,1}^{in}\left(r\right)$, inside the dielectric sphere, that opposes the *external* electric field, $E_{0}\hat{z}$, thus reducing it. Then, $E_{int,1}^{in}\left(r\right)$ will induce a first-order term in the electric polarization, $P_{1\;}^{in}\left(r\right)$, that in turn induces a first-order term in the *bound* surface charge density, $\sigma_{b,1}\left(r\right){|}_{r=a}=\hat{r}\cdot P_{1\;}^{in}\left(r\right){|}_{r=a}$, responsible for the second-order term of the *internal* electric field, $E_{int,2}^{in}\left(r\right)$, and so on. It can be easily shown that the (i)-order term of the *bound* surface charge density is $\sigma_{b,i}\left(r\right){|}_{r=a}=\hat{r}\cdot P_{i}^{in}\left(r\right){|}_{r=a}$. The (i)-order term of the polarization is easily obtained through $P_{i}^{in}\left(r\right)={\left(-(\chi_{e}/3)\right)}^{i}\chi_{e}\varepsilon_{0}E_{0}\hat{z}$. Thus, $\sigma_{b,i}\left(r\right){|}_{r=a}={\left(-(\chi_{e}/3)\right)}^{i}\chi_{e}\varepsilon_{0}E_{0}cos\theta$ and eventually the *bound* surface charge density established at the sphere-vacuum interface is $\sigma_{b}\left(r\right){|}_{r=a}=\sum_{i=0}^{\infty }\sigma_{b,i}\left(r\right){|}_{r=a}=(\sum_{i=0}^{\infty }{\left(-(\chi_{e}/3)\right)}^{i})\chi_{e}\varepsilon_{0}E_{0}cos\theta =(1/(1+\chi_{e}/3))\chi_{e}\varepsilon_{0}E_{0}cos\theta$. We see that the ‘infinite regress’ on the *bound* surface charge density results in $\sigma_{b}\left(r\right){|}_{r=a}=(3/(\chi_{e}+3))\chi_{e}\varepsilon_{0}E_{0}cos\theta =3\varepsilon_{0}((\varepsilon_{r}-1)/(\varepsilon_{r}+2))E_{0}cos\theta$. This is the exact same result obtained in Section 7.1 above by other means. The *internal* electric field ultimately produced by $\sigma_{b}\left(r\right){|}_{r=a}$ is $E_{int}^{in}\left(r\right)=-((\varepsilon_{r}-1)/(\varepsilon_{r}+2))E_{0}\hat{z}$ (notice that it opposes to $E_{ext}^{in}\left(r\right)=E_{0}\hat{z}$) and relates to the polarization through $E_{int}^{in}\left(r\right)=-(1/3\varepsilon_{0})P^{in}\left(r\right)$.

Summarizing the processes described above, the application of the *external* electric field $E_{ext}^{in}\left(r\right)=E_{0}\hat{z}$ ultimately induces inside the dielectric sphere a polarization $P^{in}\left(r\right)=3\varepsilon_{0}(\chi_{e}/(\varepsilon_{r}+2))E_{0}\hat{z}$ which establishes a *bound* surface charge density $\sigma_{b}\left(r\right){|}_{r=a}=3\varepsilon_{0}((\varepsilon_{r}-1)/(\varepsilon_{r}+2))E_{0}cos\theta ,$ which in turn produces an *internal* field $E_{int}^{in}\left(r\right)=-((\varepsilon_{r}-1)/(\varepsilon^{r}+2))E_{0}\hat{z}$, which opposes the *external* one, $E_{ext}^{in}\left(r\right)=E_{0}\hat{z}$. Thus, the *externally* applied electric field, $E_{ext}^{in}\left(r\right)=E_{0}\hat{z}$, inside the dielectric sphere evolves to $E^{in}\left(r\right)=E_{ext}^{in}\left(r\right)+E_{int}^{in}\left(r\right)=E_{0}\hat{z}-((\varepsilon_{r}-1)/(\varepsilon_{r}+2))E_{0}\hat{z}=(3/(\chi_{e}+3))E_{0}\hat{z}$. Obviously, the *internal* electric field, $E_{int}^{in}\left(r\right)$, opposes the *external* one so that $E^{in}\left(r\right)<E_{ext}^{in}\left(r\right)=E_{0}\hat{z}$. In addition, it is evident that $E_{int}^{in}\left(r\right)$ actually acts toward the depolarization of the dielectric sphere, thus it is commonly termed as depolarizing field or self field (see [8] pages 93–96; [14] pages 127, 158 and § 6.6.2; [15] page 24)). Finally, it should be stressed that these processes take place due to the finite size of the specimen; in an infinite specimen, the ‘lack of external surfaces’ will result in $\sigma_{b}\left(r\right){|}_{r=a}=0$ and $E_{int}^{in}\left(r\right)=0$, so that the electric field inside the specimen will simply be equal to the *externally* applied one, $E^{in}\left(r\right)=E_{ext}^{in}\left(r\right)=E_{0}\hat{z}$.

We note that the distinction between ‘transient’ and ‘steady’ states discussed above under the concept of an ‘infinite regress of the P-E polarization process’ is only schematic. These processes are instantaneous. Nevertheless, the series-based approach of this scheme restores qualitatively the conceptually misleading causality/feedback between $P\left(r\right)$ and $E\left(r\right)$ which is inherent in the standard P-E, $\chi_{e}$, formulation (see [8] pages 68 and 76; [15] page 186, and Section 4 above). *However, still, there is a serious quantitative obstacle: in strict mathematical terms, the above geometric series should converge only when* $|-\chi_{e}/3|<1$ *[33], and since by definition* $0\leq \chi_{e}<\infty$*, the allowed interval should be* $0\leq \chi_{e}<3$*. Nevertheless, we do not raise any doubts or constraints on the obtained solution of* $E^{in}\left(r\right)$ *and use it in the entire range,* $0\leq \chi_{e}<\infty$*. This is one of the inherent ill-defined points of the standard P-E,* $\chi_{e}$*, formulation.*

#### 7.3.2. P-D, $\chi_{\varepsilon }$, Formulation

The alternative P-D, $\chi_{\varepsilon }$, formulation ($-1\leq \chi_{\varepsilon }\leq 0$) introduced here should be immune to the conceptual and mathematical complications of the standard P-E, $\chi_{e}$, formulation discussed above. Here we check this issue by using the series approach for the electric displacement inside the dielectric sphere, $D^{in}\left(r\right)$, even if we had to. To this end we have to express $D^{in}\left(r\right)$ in the form $D^{in}\left(r\right)=D_{0}^{in}\left(r\right)+D_{int,1}^{in}\left(r\right)+D_{int,2}^{in}\left(r\right)+\cdots +D_{int,i}^{in}\left(r\right)+\cdots =\sum_{i=0}^{\infty }D_{int,i}^{in}\left(r\right)$. First, we recall that in Section 7.2 above, we have found for the inside space that the total electric displacement is $D^{in}\left(r\right)=(3/(2\chi_{\varepsilon }+3))D_{0}\hat{z}$. Due to the superposition principle, the *internal* electric displacement, $D_{int}^{in}\left(r\right)$, produced by the *bound* charges of the LHI dielectric sphere at the inside space is $D_{int}^{in}\left(r\right)=D^{in}\left(r\right)-D_{ext}^{in}\left(r\right)=(3/(2\chi_{\varepsilon }+3))D_{0}\hat{z}-D_{0}\hat{z}=(-2\chi_{\varepsilon }/(2\chi_{\varepsilon }+3))D_{0}\hat{z}$ (where $D_{ext}^{in}\left(r\right)=D_{0}\hat{z}$ is the *external* electric displacement applied by the *free* charges at the inside space). By expressing $D_{int}^{in}\left(r\right)$ through the *reverse* electric polarization, ${\tilde{P}}^{in}\left(r\right)=(3\chi_{\varepsilon }/(2\chi_{\varepsilon }+3))D_{0}\hat{z}$, we have $D_{int}^{in}\left(r\right)=(-2/3){\tilde{P}}^{in}\left(r\right)$. Now we turn our interest in finding the general term, $D_{int,i}^{in}\left(r\right)$, of the series $D^{in}\left(r\right)=\sum_{i=0}^{\infty }D_{int,i}^{in}\left(r\right)$. Obviously, the zero-order term $D_{0}^{in}\left(r\right)$ is the *external* electric displacement that originates from *free* charges, that is $D_{0}^{in}\left(r\right)=D_{0}\hat{z}$. This term will induce the zero-order term of the *reverse* electric polarization, ${\tilde{P}}_{0}^{in}\left(r\right)=\chi_{\varepsilon }D_{0}^{in}\left(r\right)=\chi_{\varepsilon }D_{0}\hat{z}$. Subsequently, ${\tilde{P}}_{0}^{in}\left(r\right)$ will produce the first-order term of the electric displacement, $D_{int,1}^{in}\left(r\right)$. By using the general relation, $D_{int}^{in}\left(r\right)=(-2/3){\tilde{P}}^{in}\left(r\right)$, found above we have for the particular case, $D_{int,1}^{in}\left(r\right)=(-2/3){\tilde{P}}_{0}^{in}\left(r\right)$. In turn, $D_{int,1}^{in}\left(r\right)$ will induce the first-order term ${\tilde{P}}_{1}^{in}\left(r\right)=\chi_{\varepsilon }D_{int,1}^{in}\left(r\right)$ that subsequently will produce the second-order term $D_{int,2}^{in}\left(r\right)=\left(-2/3\right){\tilde{P}}_{1}^{in}\left(r\right),$ and so on. Accordingly, in the general case, the (i-1)-order term of the electric displacement, $D_{int,i-1}^{in}\left(r\right)$, will induce the (i-1)-order term of the *reverse* electric polarization ${\tilde{P}}_{i-1}^{in}\left(r\right)=\chi_{\varepsilon }D_{int,i-1}^{in}\left(r\right)$ that in turn will produce an (i)-order term of the electric displacement, $D_{int,i}^{in}\left(r\right)=(-2/3){\tilde{P}}_{i-1}^{in}\left(r\right)$. Thus, we easily get the following recurrence relation $D_{int,i}^{in}\left(r\right)=(-2\chi_{\varepsilon }/3)D_{int,i-1}^{in}\left(r\right)$. By progressively expanding $D_{int,i}^{in}\left(r\right)$ down to the zero-order term we have $D_{int,i}^{in}\left(r\right)=\left(-2\chi_{\varepsilon }/3\right)D_{int,i-1}^{in}\left(r\right)=\left(-2\chi_{\varepsilon }/3\right)\left(-2\chi_{\varepsilon }/3\right)D_{int,i-2}^{in}\left(r\right)=\left(-2\chi_{\varepsilon }/3\right)\left(-2\chi_{\varepsilon }/3\right)\left(-2\chi_{\varepsilon }/3\right)D_{int,i-3}^{in}\left(r\right)=\cdots ={\left(-2\chi_{\varepsilon }/3\right)}^{i}D_{0}^{in}\left(r\right).$ Thus, the electric displacement inside the dielectric sphere is $D^{in}\left(r\right)=\sum_{i=0}^{\infty }D_{int,i}^{in}\left(r\right)=\sum_{i=0}^{\infty }{\left(-2\chi_{\varepsilon }/3\right)}^{i}D_{0}^{in}\left(r\right)=\sum_{i=0}^{\infty }{\left(-2\chi_{\varepsilon }/3\right)}^{i}D_{0}\hat{z},$ else $D^{in}\left(r\right)=\left(\sum_{i=0}^{\infty }{\left(-2\chi_{\varepsilon }/3\right)}^{i}\right)D_{0}\hat{z}.$ *Based on mathematical criteria [33], the series should converge only when* $|-2\chi_{\varepsilon }/3|<1$*. This condition is fulfilled in the entire range of* $\chi_{\varepsilon }$*, since* $-1\leq \chi_{\varepsilon }\leq 0$ *(relation (20))*.

We continue with the investigation of the ‘infinite regress’ for the *bound* surface charge density, $\sigma_{b}\left(r\right){|}_{r=a}$. The *bound* charge density, $\sigma_{b}\left(r\right){|}_{r=a}$, that is induced by the primary source, the *free* charge density, acts as secondary source and ultimately produces inside the dielectric the *internal* electric displacement, $D_{int}^{in}\left(r\right)$. Specifically, the zeroth-order term of the polarization, $P_{0}^{in}\left(r\right)=-\chi_{\varepsilon }D_{0}^{in}\left(r\right)=-\chi_{\varepsilon }D_{ext}^{in}\left(r\right)=-\chi_{\varepsilon }D_{0}\hat{z}=-\chi_{\varepsilon }\varepsilon_{0}E_{0}\hat{z}$, will induce a zeroth-order term in the *bound* surface charge density, $\sigma_{b,0}\left(r\right){|}_{r=a}=\hat{r}\cdot P_{0\;}^{in}\left(r\right){|}_{r=a}=\hat{r}\cdot \left(-\chi_{\varepsilon }D_{0}^{in}\left(r\right)\right){|}_{r=a}=\hat{r}\cdot \left(-\chi_{\varepsilon }D_{ext}^{in}\left(r\right)\right){|}_{r=a}=-\chi_{\varepsilon }D_{0}cos\theta$. Then, $\sigma_{b,0}\left(r\right){|}_{r=a}$ will produce a first-order term of the *internal* electric displacement, $D_{int,1}^{in}\left(r\right)$, inside the dielectric sphere, that opposes the *external* electric displacement, $D_{0}\hat{z}$, thus reducing it. Then, $D_{int,1}^{in}\left(r\right)$ will induce a first-order term in the electric polarization, $P_{1\;}^{in}\left(r\right)$, that in turn induces a first-order term in the *bound* surface charge density, $\sigma_{b,1}\left(r\right){|}_{r=a}=\hat{r}\cdot P_{1\;}^{in}\left(r\right){|}_{r=a}=\hat{r}\cdot (-\chi_{\varepsilon }D_{int,1}^{in}\left(r\right)){|}_{r=a}$, responsible for the second-order term of the *internal* electric displacement, $D_{int,2}^{in}\left(r\right)$, and so on. It can be easily shown that the (i)-order term of the *bound* surface charge density is $\sigma_{b,i}\left(r\right){|}_{r=a}=\hat{r}\cdot P_{i}^{in}\left(r\right){|}_{r=a}$. The (i)-order term of the polarization is easily obtained through $P_{i}^{in}\left(r\right)={\left(-(2\chi_{\varepsilon }/3)\right)}^{i}(-\chi_{\varepsilon }D_{0})\hat{z}.$ Thus, $\sigma_{b,i}\left(r\right){|}_{r=a}={\left(-(2\chi_{\varepsilon }/3)\right)}^{i}(-\chi_{\varepsilon }D_{0})cos\theta$ and eventually the *bound* surface charge density established at the sphere-vacuum interface is $\sigma_{b}\left(r\right){|}_{r=a}=\sum_{i=0}^{\infty }\sigma_{b,i}\left(r\right){|}_{r=a}=(\sum_{i=0}^{\infty }{\left(-(2\chi_{\varepsilon }/3)\right)}^{i})(-\chi_{\varepsilon }D_{0})cos\theta =(1/(1+2\chi_{\varepsilon }/3))(-\chi_{\varepsilon }D_{0})cos\theta$. We see that the ‘infinite regress’ on the *bound* surface charge density results in $\sigma_{b}\left(r\right){|}_{r=a}=-(3\chi_{\varepsilon }/(2\chi_{\varepsilon }+3))D_{0}cos\theta$, where $D_{0}=\varepsilon_{0}E_{0}$. This is the exact same result obtained in Section 7.2 above by other means. The *internal* electric displacement ultimately produced by $\sigma_{b}\left(r\right){|}_{r=a}$ is $D_{int}^{in}\left(r\right)=-(2\chi_{\varepsilon }/(2\chi_{\varepsilon }+3))D_{0}\hat{z}$. Notice that since ${-1\leq \chi }_{\varepsilon }\leq 0$ $D_{int}^{in}\left(r\right)$ is homoparallel to $D_{ext}^{in}\left(r\right)=D_{0}\hat{z}$ so that the *total* electric displacement, $D^{in}\left(r\right)$, inside the LHI dielectric sphere is higher that the externally applied, $D_{ext}^{in}\left(r\right)=D_{0}\hat{z}$, as expected. *Most importantly, we see that even for the bound surface charge density investigated here, based on mathematical criteria [33], the above series should converge only when* $|-2\chi_{\varepsilon }/3|<1$*. This condition is fulfilled in the entire range of* $\chi_{\varepsilon }$ *since* $-1\leq \chi_{\varepsilon }\leq 0$ *(relation (20)).*

The above discussion clearly documents that the P-D, $\chi_{\varepsilon }$ formulation is inherently free of any misleading issue and mathematical flaw from which the standard P-E, $\chi_{e}$ formulation suffers. More cases are presented analytically on the same comparative basis in Appendix A.2 of the Appendix A.

Returning to the issue of quantitative consistency of the two formulations, obviously all standard physical entities should attain the exact same form, irrespectively of the employed one. As an example, in Appendix A.3 of the Appendix A we show that the atomic/molecular polarizability (Clausius-Mossotti relation) attains the same form whether we employ the standard formulation P-E, $\chi_{e}$, or the new one P-D, $\chi_{\varepsilon }$.

Finally, long time ago, it was fairly documented that the standard P-E electric susceptibility, $\chi_{e}$, (relations (1)–(6)) cannot obtain negative values, relation (4), in systems under thermodynamic equilibrium (see [10] §14 pages 58–59 and [34]). Clearly, the new P-D electric susceptibility, $\chi_{\varepsilon }$, introduced here (relations (17)–(22)) does not violate this principle since as can be seen by the relation $\chi_{\varepsilon }=-\chi_{e}/(1+\chi_{e})$, when the P-D electric susceptibility, $\chi_{\varepsilon }$, ranges within $-1\leq \chi_{\varepsilon }\leq 0$, the standard P-E electric susceptibility, $\chi_{e}$, spans within $0\leq \chi_{e}<\infty$ as it should. However, the discussion on “matter” (ordinary, out of thermodynamic equilibrium, and extraordinary, artificial metamaterials) which exhibits negative values of the standard P-E electric susceptibility, $-1\leq \chi_{e}\leq 0$, has gained much interest in the recent decades [35,36,37]. Such property would be highly beneficial for applications. The present work of ours on the P-D electric susceptibility, $-1\leq \chi_{\varepsilon }\leq 0$, could provide alternative means to access this issue.

## 8. Technical Advantages of the P-D, $\chi_{\varepsilon }$, Formulation and Perspectives

Here we discuss the technical advantages of the P-D, $\chi_{\varepsilon }$ formulation in comparison to the P-E, $\chi_{e}$ one. The comparison is not restricted to the LHI dielectrics discussed in this work. Probably, the technical advantages of the P-D, $\chi_{\varepsilon }$ formulation become apparent more effectively when non-linear, inhomogeneous, and anisotropic dielectrics are considered. Accordingly, here we present introductory results for a representative subcategory of linear and isotropic, however, inhomogeneous dielectrics.

Starting from the irrotationality of the electric field (relation (13)) and using the constitutive relation of the P-D, $\chi_{\varepsilon }$ formulation (relation (18)), we get the following relation:

(67) $$\nabla \times \left(D\left(r\right)/\varepsilon_{\varepsilon }\left(r\right)\right)=0$$

With relatively simple algebra we can finally obtain the relation:

(68) $$\nabla \times D\left(r\right)-(\nabla \varepsilon_{\varepsilon }\left(r\right)/\varepsilon_{\varepsilon }\left(r\right))\times D\left(r\right)=0\;$$

From this relation, we easily see that the *primary* vector field, $D\left(r\right)$, is irrotational $\left(\nabla \times D\left(r\right)=0\right)$ only when the terms $\nabla \varepsilon_{\varepsilon }\left(r\right)$ and $D\left(r\right)$ are parallel so that $\left(\nabla \varepsilon_{\varepsilon }\left(r\right)\right)\times D\left(r\right)=0$. This condition has the following physical meaning: even in inhomogeneous dielectrics, the *primary* vector field, $D\left(r\right)$, can still depend *solely* on free charges (being independent of the material’s properties), when the inhomogeneity $\left(\nabla \varepsilon_{\varepsilon }\left(r\right)\right)$ evolves along its direction.

Here we extend the discussion of the LHI dielectric sphere made in Section 7 to the linear and isotropic, however, inhomogeneous case. This is another popular problem discussed in the literature in respect to subwavelength scattering and cloak applications. In [30], a radially inhomogeneous core-shell dielectric sphere was considered for utilization in subwavelength scattering. The core was LHI, while the shell was linear and isotropic, however, inhomogeneous with permittivity function ε(r). This core-shell dielectric sphere was embedded in a LHI dielectric host medium and was subjected to an external, uniform electric field. For the radial dependence of the permittivity function ε(r), various cases were considered, such as power-law, linear exponential, and inverse linear exponential [30]. Also, in [32], a core-shell spherical structure was investigated with the permittivity of the core and shell ε_1_ and ε_2_, respectively. This building element was embedded inside a host medium of permittivity ε_m_. An external, uniform electric field was applied. The core of the structure was the area to be invisible under the protective action of the shell. All core, shell, and host medium were LHI dielectrics (with the shell having both positive and negative permittivity), while the overall core-shell sphere was obviously inhomogeneous.

Thus, right below, we consider a relevant example. A linear and isotropic dielectric sphere of radius a is radially inhomogeneous with P-D electric susceptibility $\chi_{\varepsilon }(r)$ depending solely on the distance, r, from its center, which coincides with the origin of the spherical coordinate system. A *free* point charge Q resides at the origin, producing the *external* component, $D_{ext}\left(r\right)$, of the *primary* vector field, $D\left(r\right)$, which polarizes the dielectric sphere. Here we briefly tackle the problem in the framework of the P-D, $\chi_{\varepsilon }$ formulation following the argumentation of Section 5.3 and especially the rule of thumb illustrated in Figure 1 (the simple case of a LHI dielectric sphere with a point charge Q at its center is presented in Appendix A.2 of the Appendix A). First, we realize that the electric susceptibility/permittivity $\chi_{\varepsilon }(r)/\varepsilon_{\varepsilon }(r)$ depend solely on the radial distance, r. Apparently, the *external* component of the *primary* vector field has only radial component and depends solely on the radial distance, r, as well, that is $D_{ext}\left(r\right)=D_{ext}\left(r\right)\hat{r}=(Q/4\pi r^{2})\hat{r}$. Also, due to the linear and isotropic character of the dielectric sphere, all vector fields $D\left(r\right)$, $P\left(r\right),$ and $E\left(r\right)$ will exhibit only radial component and will depend solely on the radial distance, r. Under these circumstances, in relation (68), the terms $\nabla \varepsilon_{\varepsilon }\left(r\right)$ and $D\left(r\right)$ are parallel so that $D\left(r\right)$ is irrotational. Accordingly, we are able to calculate the total *primary* vector field D(r) beforehand (that is, independently of E(r) and P(r)) by using the technical means discussed in Section 5.1 and Section 5.2. More importantly, the calculations can be facilitated by using the detailed outcome of Section 5.3, as summarized in the work-flow diagram presented in Figure 1. In this case, the *external* component of the primary vector field, $D_{ext}(r)$, is exclusively *normal* to the dielectric sphere-vacuum *interface* so that the total *primary* vector field $D\left(r\right)$ depends solely on the *free* charges and coincides with $D_{ext}(r)$. Thus, the total *primary* vector field reads simply $D\left(r\right)=(Q/4\pi r^{2})\hat{r}$. The total *secondary* vector fields, $P\left(r\right)$ and $E\left(r\right)$, can be obtained directly by using relations (17) and (18), respectively. It should be noted that the above discussion is generic; the procedure followed and the obtained results are the same irrespectively of the functional form of the electric susceptibility/permittivity $\chi_{\varepsilon }(r)/\varepsilon_{\varepsilon }(r)$. Finally, by using relation (34), we can translate our findings from the P-D, $\chi_{\varepsilon }$ notation to the P-E, $\chi_{e}$ one.

Dielectrics with inhomogeneous properties not necessarily directed along the *external* component of the *primary* vector field, $D\left(r\right)$, and the more general case of non-linear, inhomogeneous, and anisotropic dielectrics will be discussed in future work.

## 9. Conclusions

Here we revisited the case of electrostatics in material systems comprising of *free* charges and LHI dielectrics. We focused on $D\left(r\right)$ and proved that it is sufficient to describe the underlying physics and to accomplish all algebraic calculations in such systems. We introduced the P-D electric susceptibility, *χ_ε_*, through the constitutive relation $\tilde{P}\left(r\right)=-P\left(r\right)=\chi_{\varepsilon }D\left(r\right)$, with $-1\leq \chi_{\varepsilon }\leq 0$. We clearly showed how the *primary* vector field, $D\left(r\right)$, can be calculated directly from the free charge density, $\rho_{f}$, and the electric susceptibility, *χ_ε_*, of the LHI dielectrics, beforehand, without any knowledge of the *secondary* vector fields, $P\left(r\right)$ and $E\left(r\right)$. The latter, if needed, can be easily calculated from $D\left(r\right)$. The new P-D, $\chi_{\varepsilon }$, formulation ($\tilde{P}\left(r\right)=-P\left(r\right)=\chi_{\varepsilon }D\left(r\right)$, with $-1\leq \chi_{\varepsilon }\leq 0$) advances the understanding of underlying physics in LHI materials and has some technical advantages in respect to the standard P-E, $\chi_{e}$, one ($P\left(r\right)=\chi_{e}\varepsilon_{0}E\left(r\right)$, with $0\leq \chi_{e}<\infty$). First, the P-D, $\chi_{\varepsilon }$, formulation restores efficiently all conceptual and mathematical flaws which are inherent to the standard P-E, $\chi_{e}$, one (misleading causality/feedback between $P\left(r\right)$ and $E\left(r\right)$ and non-compliance with the necessary convergence criteria). Second, the P-D, $\chi_{\varepsilon }$, formulation unveils the underlying physics of all physical entities of electrostatics in a more direct and consistent way. Third, the P-D, $\chi_{\varepsilon }$, formulation symmetrizes the electrostatics analogously to the M-H, $\chi_{m}$, formulation of magnetostatics. Fourth, the P-D, $\chi_{\varepsilon }$, formulation proposes means, such as the *free* scalar potential, $U_{f}\left(r\right)$, that can technically facilitate analytical/computational calculations. Except for the LHI dielectrics discussed here, our concept can possibly be useful for the treatment of non-linear, inhomogeneous, and anisotropic dielectrics.

## Data Availability

The data that supports the findings of this study are available within the article.

## Appendix A

This Appendix is structured as follows: Appendix A.1 discusses important issues on both the foreground mathematics and the underlying physics of the electric displacement, $D\left(r\right)$, which is the *primary* vector field in the P-D, $\chi_{\varepsilon }$, description. Appendix A.2 addresses representative problems of *free* charges combined with LHI dielectrics by using both formulations, the standard P-E, $\chi_{e}$, and the alternative P-D, $\chi_{\varepsilon }$, to clarify a number of important issues on the documentation of the P-D, $\chi_{\varepsilon }$, description. Appendix A.3 assesses the efficacy of the P-D, $\chi_{\varepsilon }$, formulation in the description of some standard physical parameters of LHI dielectric materials; the Clausius-Mossotti equation is considered with both formulations, the standard P-E, $\chi_{e}$, and the alternative P-D, $\chi_{\varepsilon }$.

### Appendix A.1. Helmholtz Decomposition of $D\left(r\right)$

Here we investigate the Helmholtz decomposition of the electric displacement, $D\left(r\right)$, to clarify some important issues on both the foreground mathematics and the underlying physics of this vector field. We stress that the discussion below is exclusively focused on $D\left(r\right)$ since in the P-D, $\chi_{\varepsilon }$, formulation, $D\left(r\right)$ is the *primary* vector field that should be calculated beforehand, independently of the *secondary* vector fields, the *reverse* electric polarization, $\tilde{P}\left(r\right)=-P\left(r\right)$, and the electric field, $E\left(r\right)$. Then $\tilde{P}\left(r\right)=-P\left(r\right)$ and $E\left(r\right)$ can be easily calculated from $D\left(r\right)$.

The most important of the investigated issues is to determine the conditions under which $D\left(r\right)$ depends *solely* on free charges or it depends on the dielectric properties, as well. Below, we document formally that, in the general case, $D\left(r\right)$ does not depend solely on *free* charges. Under specific circumstances, the properties of the LHI dielectrics are imprinted onto $D\left(r\right)$ through the relevant boundary conditions that should be satisfied at the *interfaces* of distinct media having different dielectric properties (thus, discontinuities of the electric polarization/susceptibility exist at such interfaces).

The most basic situation refers to a piecewise continuous $D\left(r\right)$ existing in the entire dielectric space (including vacuum as dielectric) which is divided in two distinct dielectric subspaces termed ‘in’ and ‘out’. By applying the Helmholtz theorem [21,22,23,24] on $D\left(r\right),$ we have the following expression:

(A1) $$D\left(r\right)=-\nabla \left[\frac{1}{4\pi }\int_{V^{/}}\frac{\nabla^{/}\cdot D\left(r^{/}\right)}{\left|r-r^{/}\right|}dV^{/}+\frac{1}{4\pi }\oint_{S^{/}}\frac{{\hat{n}}^{/}\cdot \left(D_{out}\left(r^{/}\right)-D_{in}\left(r^{/}\right)\right)}{\left|r-r^{/}\right|}dS^{/}\right]\;+\nabla \times \left[\frac{1}{4\pi }\int_{V^{/}}\frac{\nabla^{/}\times D\left(r^{/}\right)}{\left|r-r^{/}\right|}dV^{/}+\frac{1}{4\pi }\oint_{S^{/}}\frac{{\hat{n}}^{/}\times \left(D_{out}\left(r^{/}\right)-D_{in}\left(r^{/}\right)\right)}{\left|r-r^{/}\right|}dS^{/}\right]\;$$

Here, $V^{/}$ is the entire space, comprising of the *interiors* of subspaces ‘in’ and ‘out’, wherein $\nabla^{/}\cdot D\left(r^{/}\right)$ and $\nabla^{/}\times D\left(r^{/}\right)$ should be known, while the closed surface $S^{/}$ is the *interface* of subspaces ‘in’ and ‘out’ where ${\hat{n}}^{/}\cdot \left(D_{out}\left(r^{/}\right)-D_{in}\left(r^{/}\right)\right)$ and ${\hat{n}}^{/}\times \left(D_{out}\left(r^{/}\right)-D_{in}\left(r^{/}\right)\right)$ should be known, as well. Finally, ${\hat{n}}^{/}$ refers to the unit vector that is normal to $S^{/}$, directed from subspace ‘in’ to subspace ‘out’.

From these relations, we can define two functions, a scalar, $U_{f}\left(r\right)$, (first parenthesis) and a vector, $A_{b}\left(r\right)$, (second parenthesis) through:

(A2) $$U_{f}\left(r\right)=\frac{1}{4\pi \varepsilon_{0}}\int_{V^{/}}\frac{\nabla^{/}\cdot D\left(r^{/}\right)}{\left|r-r^{/}\right|}dV^{/}+\frac{1}{4\pi \varepsilon_{0}}\oint_{S^{/}}\frac{{\hat{n}}^{/}\cdot \left(D_{out}\left(r^{/}\right)-D_{in}\left(r^{/}\right)\right)}{\left|r-r^{/}\right|}dS^{/}\;$$

and

(A3) $$A_{b}\left(r\right)=\frac{1}{4\pi }\int_{V^{/}}\frac{\nabla^{/}\times D\left(r^{/}\right)}{\left|r-r^{/}\right|}dV^{/}+\frac{1}{4\pi }\oint_{S^{/}}\frac{{\hat{n}}^{/}\times \left(D_{out}\left(r^{/}\right)-D_{in}\left(r^{/}\right)\right)}{\left|r-r^{/}\right|}dS^{/}\;.\;$$

Thus, in the general case, $D\left(r\right)$ can be determined through:

(A4) $$D\left(r\right)=-\varepsilon_{0}\nabla U_{f}\left(r\right)+\nabla \times A_{b}\left(r\right)\;.$$

We call $U_{f}\left(r\right)$ the *free* scalar potential and $A_{b}\left(r\right)$ the *bound* vector potential for reasons that will become clear below. It is easily seen that for the case of electrostatics discussed here, the first relation is actually the law of Coulomb, where the two terms $\nabla^{/}\cdot D\left(r^{/}\right)$ and ${\hat{n}}^{/}\cdot \left(D_{out}\left(r^{/}\right)-D_{in}\left(r^{/}\right)\right)$ act as volume and surface sources of $U_{f}\left(r\right)$ and consequently of $D\left(r\right)$. Likewise, it is evident that the second relation is practically the law of Biot-Savart, where the two terms $\nabla^{/}\times D\left(r^{/}\right)$ and ${\hat{n}}^{/}\times \left(D_{out}\left(r^{/}\right)-D_{in}\left(r^{/}\right)\right)$ act as volume and surface sources of $A_{b}\left(r\right)$ and consequently of $D\left(r\right)$ [16,17,18].

Now, let us clarify the role of each one of the above candidate sources for the case of electrostatics in the systems comprising of *free* charges and LHI dielectrics discussed here. Starting with the volume sources, obviously, at the *interior* of subspaces ‘in’ and ‘out’, the relation $\nabla^{/}\cdot D\left(r^{/}\right)=\rho_{f}\left(r^{/}\right)$ should hold wherever a non-zero $\rho_{f}\left(r^{/}\right)$ exists, else $\nabla^{/}\cdot D\left(r^{/}\right)=$ 0. In addition, at the *interior* of subspaces ‘in’ and ‘out’, $D\left(r\right)$ is obviously irrotational, $\nabla^{/}\times D\left(r^{/}\right)=0$. As we show below, this does not necessarily hold at the *interfaces* between distinct dielectrics, even when they are LHI. Continuing with the surface sources, at the *interface* $S^{/}$, the following two fundamental conditions should hold for the electric field, $E\left(r\right)$ [8,9,11,13,14,15]. Below we review these boundary conditions on $E\left(r\right)$ which we ultimately ‘translate’ in respect to $D\left(r\right)$ since this is the *primary* vector field according to the P-D, $\chi_{\varepsilon }$, formulation.

The first fundamental boundary condition refers to the *normal* to $S^{/}$ component of $E\left(r\right)$ and reads:

(A5) $${\hat{n}}^{/}\cdot \left(E_{out}\left(r^{/}\right)-E_{in}\left(r^{/}\right)\right){|}_{S^{/}}=\sigma \left(r^{/}\right){|}_{S^{/}}/\varepsilon_{0}\;.$$

Given that relations $D\left(r\right)=\varepsilon_{0}E\left(r\right)+P(r)$ and $\sigma_{f}\left(r\right){|}_{S}+\sigma_{b}\left(r\right){|}_{S}=\sigma \left(r\right){|}_{S}$ hold, relation (A5) transforms into the two independent ones:

(A6) $${\hat{n}}^{/}\cdot \left(D_{out}\left(r^{/}\right)-D_{in}\left(r^{/}\right)\right){|}_{S^{/}}=\sigma_{f}\left(r^{/}\right){|}_{S^{/}}$$

and

(A7) $$(-{\hat{n}}^{/})\cdot \left(P_{out}\left(r^{/}\right)-P_{in}\left(r^{/}\right)\right){|}_{S^{/}}={\hat{n}}^{/}\cdot \left({\tilde{P}}_{out}\left(r^{/}\right)-{\tilde{P}}_{in}\left(r^{/}\right)\right){|}_{S^{/}}=\sigma_{b}\left(r^{/}\right){|}_{S^{/}}.$$

The second fundamental boundary condition on the *tangential* to $S^{/}$ components of $E\left(r\right)$ is:

(A8) $${\hat{n}}^{/}\times \left(E_{out}\left(r^{/}\right)-E_{in}\left(r^{/}\right)\right){|}_{S^{/}}=0.$$

Again, by using $D\left(r\right)=\varepsilon_{0}E\left(r\right)+P(r)$, relation (A8) simply transforms to:

(A9) $${\hat{n}}^{/}\times \left(D_{out}\left(r^{/}\right)-D_{in}\left(r^{/}\right)\right){|}_{S^{/}}={\hat{n}}^{/}\times \left(P_{out}\left(r^{/}\right)-P_{in}\left(r^{/}\right)\right){|}_{S^{/}}\;=\left({\tilde{P}}_{out}\left(r^{/}\right)-{\tilde{P}}_{in}\left(r^{/}\right)\right)\times {\hat{n}}^{/}{|}_{S^{/}}.$$

Thus, we have three independent boundary conditions on $D\left(r\right)$ and $\tilde{P}\left(r\right)=-P\left(r\right)$ given by relations (A6), (A7), and (A9). Below, we discuss each one of them to understand its physical content in respect to $D\left(r\right)$.

We start from the latter boundary condition, relation (A9). By recalling that in the P-D, $\chi_{\varepsilon }$, formulation, $\tilde{P}\left(r\right)=-P\left(r\right)=\chi_{\varepsilon }D(r)$, relation (A9) evolves to:

(A10) $${\hat{n}}^{/}\times \left(D_{out}\left(r^{/}\right)-D_{in}\left(r^{/}\right)\right){|}_{S^{/}}=\left(\chi_{\varepsilon ,out}D_{out}\left(r^{/}\right)-\chi_{\varepsilon ,in}D_{in}\left(r^{/}\right)\right)\times {\hat{n}}^{/}{|}_{S^{/}}$$

that by simple rearrangement of terms further transforms to:

(A11) $$(1+\chi_{\varepsilon ,out}){\hat{n}}^{/}\times D_{out}\left(r^{/}\right){|}_{S^{/}}=(1+\chi_{\varepsilon ,in}){\hat{n}}^{/}\times D_{in}\left(r^{/}\right){|}_{S^{/}}.$$

By recalling that in the P-D, $\chi_{\varepsilon }$, formulation, the relative permittivity is given by $\varepsilon_{r\varepsilon }={\left(1+\chi_{\varepsilon }\right)}^{-1}$ (relation (21) of the article), the above relation (A11) takes another equivalent version:

(A12) $${\hat{n}}^{/}\times (D_{out}\left(r^{/}\right)/\varepsilon_{r\varepsilon ,out}){|}_{S^{/}}={\hat{n}}^{/}\times (D_{in}\left(r^{/}\right)/\varepsilon_{r\varepsilon ,in}){|}_{S^{/}}.$$

Relations (A11) and (A12) reveal that the *tangential* components of $D_{out}\left(r\right)$ and $D_{in}\left(r\right)$ are linearly dependent at the *interface* $S^{/}$. Based on this fact, the former boundary condition obtains the following two, quite informative, equivalent forms:

(A13) $${\hat{n}}^{/}\times \left(D_{out}\left(r^{/}\right)-D_{in}\left(r^{/}\right)\right){|}_{S^{/}}=((\chi_{\varepsilon ,in}-\chi_{\varepsilon ,out})/(1+\chi_{\varepsilon ,in})){\hat{n}}^{/}\times D_{out}\left(r^{/}\right){|}_{S^{/}}$$

(A14) $$=((\chi_{\varepsilon ,in}-\chi_{\varepsilon ,out})/(1+\chi_{\varepsilon ,out})){\hat{n}}^{/}\times D_{in}\left(r^{/}\right){|}_{S^{/}}.$$

In practical terms, relations (A11) and (A12) are the most useful of all versions since they are ready to be applied. More general, in any of its versions, (A10)–(A14), this is the most important boundary condition for the P-D, $\chi_{\varepsilon }$, formulation due to the following reasons: It is the only three boundary conditions that brings the dielectric properties (i.e., the P-D electric susceptibility, $\chi_{\varepsilon }$) at the proscenium of the algebraic calculations and inevitably imprints $\chi_{\varepsilon }$ onto all relevant physical entities of the problem (vector fields, scalar/vector potentials, etc.), obviously starting from the *tangential* components of the electric displacement, D(r). Then, since $\tilde{P}\left(r\right)=-P\left(r\right)$ and E(r) are directly calculated from D(r), they should also depend on the properties of the LHI dielectrics, that is the electric susceptibility, $\chi_{\varepsilon }$. All other relevant physical entities, i.e., *bound* surface charge density, dipole moment, etc., will depend on the dielectric properties as well.

Next, we proceed with the second boundary condition, relation (A7). Once again, by recalling the basic relation $\tilde{P}\left(r\right)=-P\left(r\right)=\chi_{\varepsilon }D(r)$ of the P-D, $\chi_{\varepsilon }$, formulation, relation (A7) gets the form:

(A15) $${\hat{n}}^{/}\cdot \left(\chi_{\varepsilon ,out}D_{out}\left(r^{/}\right)-\chi_{\varepsilon ,in}D_{in}\left(r^{/}\right)\right){|}_{S^{/}}=\sigma_{b}\left(r^{/}\right){|}_{S^{/}}.$$

A superficial interpretation of this boundary condition will possibly lead to the following misleading message: this relation also imprints the P-D electric susceptibility, $\chi_{\varepsilon }$, onto the *normal* component of the electric displacement, D(r), a situation completely analogous to the one discussed above, relations (A10)–(A14), for the *tangential* components of D(r). However, this is not the case. Relation (A15) should only be used to accomplish the self-consistent solution of the problem through the determination of the *bound* surface charge density, $\sigma_{b}\left(r^{/}\right){|}_{S^{/}}$, from the *normal* component of the electric displacement, D(r), which should already be known. To recover this information, we turn our interest to the last boundary condition that refers to the *normal* component of D(r) as well.

Indeed, the third boundary condition, relation (A6), already refers to the *normal* component of D(r), so we do not have much to do. We just reproduce it for convenience:

(A16) $${\hat{n}}^{/}\cdot \left(D_{out}\left(r^{/}\right)-D_{in}\left(r^{/}\right)\right){|}_{S^{/}}=\sigma_{f}\left(r^{/}\right){|}_{S^{/}}\;.$$

This relation clearly states that the discontinuity of the *normal* component of D(r) at the *interface* $S^{/}$ should depend solely on *free* charges. This information should be used in accordance with relation (A15) above, (that also relates to the *normal* component of D(r)) for the determination of the *bound* surface charge density, $\sigma_{b}\left(r^{/}\right){|}_{S^{/}}$.

The boundary conditions discussed until now, relations (A10)–(A16), refer to the *primary* vector field of electrostatics, D(r), that relates to the *free* scalar potential, $U_{f}\left(r\right)$, and the *bound* vector potential, $A_{b}\left(r\right)$, respectively, through relation (A4). In turn, $U_{f}\left(r\right)$ and $A_{b}\left(r\right)$ are given by relation (A2) and relation (A3), respectively. By recalling that for the LHI dielectrics discussed here $\nabla^{/}\cdot D\left(r^{/}\right)=\rho_{f}\left(r^{/}\right)$ and $\nabla^{/}\times D\left(r^{/}\right)=0$ and by using relations (A13) and (A16), the functions $U_{f}\left(r\right)$ and $A\left(r\right)$ become:

(A17) $$U_{f}\left(r\right)=\frac{1}{4\pi \varepsilon_{0}}\int_{V^{/}}\frac{\rho_{f}\left(r^{/}\right)}{\left|r-r^{/}\right|}dV^{/}+\;\frac{1}{4\pi \varepsilon_{0}}\oint_{S^{/}}\frac{\sigma_{f}\left(r^{/}\right){|}_{S^{/}}}{\left|r-r^{/}\right|}dS^{/}$$

and

(A18) $$A_{b}\left(r\right)=\frac{1}{4\pi }\oint_{S^{/}}\frac{\left(\chi_{\varepsilon ,out}D_{out}\left(r^{/}\right)-\chi_{\varepsilon ,in}D_{in}\left(r^{/}\right)\right)\times {\hat{n}}^{/}{|}_{S^{/}}}{\left|r-r^{/}\right|}dS^{/}\;=\frac{1}{4\pi }\frac{\chi_{\varepsilon ,in}-\chi_{\varepsilon ,out}}{1+\chi_{\varepsilon ,in}}\oint_{S^{/}}\frac{{\hat{n}}^{/}\times D_{out}\left(r^{/}\right){|}_{S^{/}}}{\left|r-r^{/}\right|}dS^{/}\;=\frac{1}{4\pi }\frac{\chi_{\varepsilon ,in}-\chi_{\varepsilon ,out}}{1+\chi_{\varepsilon ,out}}\oint_{S^{/}}\frac{{\hat{n}}^{/}\times D_{in}\left(r^{/}\right){|}_{S^{/}}}{\left|r-r^{/}\right|}dS^{/}\;.$$

From the integral relation (A17), we clearly see that $U_{f}\left(r\right)$ relates solely on *free* charges, $\rho_{f}\left(r^{/}\right)$ and $\sigma_{f}\left(r^{/}\right){|}_{S^{/}}$, thus we formally call it *free* scalar electric potential. Similarly, the integral relation (A18) clearly reveals that $A_{b}\left(r\right)$ depends on any discontinuity/mismatch of the *tangential* components of $\tilde{P}\left(r\right)=-P\left(r\right)$, of the LHI dielectrics at the *interface* $S^{/}$ of the two distinct subspaces ‘in’ and ‘out’, thus we assign the term *bound* vector potential (else, *polarization* vector potential). Most importantly, relations (A17) and (A18) evidence that in the general case, $D\left(r\right)$ depends on both the *free* charges through $U_{f}\left(r\right)$ and the properties of the LHI dielectrics, i.e., the P-D electric susceptibility $\chi_{\varepsilon }$, through $A_{b}\left(r\right)$. Specifically, relation (A18) documents that the *bound* vector potential, $A_{b}\left(r\right)$, is non-zero when the following two conditions are fulfilled at the same time: first, the dielectric properties, i.e., the P-D electric susceptibility, $\chi_{\varepsilon }$, of distinct dielectrics should be discontinuous at their *interface* $S^{/}$, and second, $D\left(r\right)$ and the induced $\tilde{P}\left(r\right)=-P\left(r\right)=\chi_{\varepsilon }D\left(r\right)$ should have at least one component *tangential* to the *interface* $S^{/}$. The first condition is determined by the intrinsic properties of the employed dielectrics, while the second is determined by external characteristics such as the geometry of the employed building units (i.e., *free* charges and LHI dielectrics) and their relative orientation (see Section 5.3 and especially the rule of thumb illustrated in Figure 1).

The discontinuity of $\chi_{\varepsilon }$ and the existence of at least one *tangential* component at the *interface* $S^{/}$ of distinct dielectric media have strong implications on both the physics and the mathematics of these systems; first, as already discussed above, it dictates that $D\left(r\right)$ should depend on both the *free* charges and the properties of the employed LHI dielectrics, second, it determines the *non* irrotational behavior of $D\left(r\right)$, and, third, it defines the *non* continuous character of $U_{f}\left(r\right)$. These issues have been discussed in the article, as well (see Section 5.2 and Section 5.3 of the article). While the electric displacement, $D\left(r\right)$, is irrotational and the *free* scalar potential, $U_{f}\left(r\right)$, is continuous at the *interior* of any LHI dielectric medium, they do *not* necessarily preserve this behavior at the *interface* $S^{/}$ of distinct dielectric media. The boundary conditions (A10)–(A14) imply that a discontinuity in the *tangential* components of $\tilde{P}\left(r\right)=-P(r)$ at the *interface* $S^{/}$ of different dielectric media (that clearly always exists due to the discontinuous change of $\chi_{\varepsilon }$) will result in a *non* irrotational behavior of $D\left(r\right)$ at $S^{/}$. In turn, this will motivate a *non* continuous character of $U_{f}\left(r\right)$ at the *interface* $S^{/}$, as well. We stress once again that in this case, $D\left(r\right)$ will depend on both the *free* charges and the properties of the LHI dielectrics. In this case, the desired solution of $D\left(r\right)$ can be obtained straightforwardly by finding $U_{f}\left(r\right)$ through the integral relation (A17) and $A_{b}\left(r\right)$ through the integral relation (A18) so that $D\left(r\right)$ is ultimately calculated through relation (A4). However, alternative, more convenient practices can be employed to find $D\left(r\right)$. First, we recall that the surface terms in relations (A17) and (A18) are actually the boundary conditions (A16) and equivalent forms (A10)–(A14), respectively. Second, we realize that $D\left(r\right)$ is determined only by the volume source of $U_{f}\left(r\right)$, $\nabla^{/}\cdot D\left(r^{/}\right)=\rho_{f}\left(r^{/}\right)$. Indeed, since $A_{b}\left(r\right)$ has no volume source, $\nabla^{/}\times D\left(r^{/}\right)=0$, it is actually involved only in the boundary condition of any of the equivalent relations (A10)–(A14). Third, from relation (A4) we realize that $U_{f}\left(r\right)$ can be used to exclusively satisfy all boundary conditions which $D\left(r\right)$ should obey, including the one in which $A_{b}\left(r\right)$ is involved. Once this is realized, $A_{b}\left(r\right)$ is no longer needed. However, the boundary conditions we have at hand, relations (A10)–(A14), (A15), and (A16), refer to D(r). Thus, we have to ‘translate’ them to be applicable to $U_{f}\left(r\right)$. Accordingly, once we assume that the boundary conditions that $D\left(r\right)$ should obey at the *interface* $S^{/}$ can be satisfied exclusively by $U_{f}\left(r\right)$, relations (A10)–(A14), (A15), and (A16) obtain the form discussed below.

We start with relations (A10)–(A14) on the *tangential* components of $D\left(r\right)$. In respect to $U_{f}\left(r\right)$, these relations take, one by one, the following forms:

(A19) $${\hat{n}}^{/}\times \left(\nabla U_{f,out}\left(r^{/}\right)-\nabla U_{f,in}\left(r^{/}\right)\right){|}_{S^{/}}=\left(\chi_{\varepsilon ,out}\nabla U_{f,out}\left(r^{/}\right)-\chi_{\varepsilon ,in}\nabla U_{f,in}\left(r^{/}\right)\right)\times {\hat{n}}^{/}{|}_{S^{/}}$$

else

(A20) $$(1+\chi_{\varepsilon ,out}){\hat{n}}^{/}\times \nabla U_{f,out}\left(r^{/}\right){|}_{S^{/}}=(1+\chi_{\varepsilon ,in}){\hat{n}}^{/}\times \nabla U_{f,in}\left(r^{/}\right){|}_{S^{/}}\;$$

else

(A21) $${\hat{n}}^{/}\times \nabla U_{f,out}\left(r^{/}\right)/\varepsilon_{r\varepsilon ,out}{|}_{S^{/}}={\hat{n}}^{/}\times \nabla U_{f,in}\left(r^{/}\right)/\varepsilon_{r\varepsilon ,in}{|}_{S^{/}}$$

else

(A22) $${\hat{n}}^{/}\times \left(\nabla U_{f,out}\left(r^{/}\right)-\nabla U_{f,in}\left(r^{/}\right)\right){|}_{S^{/}}=((\chi_{\varepsilon ,in}-\chi_{\varepsilon ,out})/(1+\chi_{\varepsilon ,in})){\hat{n}}^{/}\times \nabla U_{f,out}\left(r^{/}\right){|}_{S^{/}}$$

(A23) $$=((\chi_{\varepsilon ,in}-\chi_{\varepsilon ,out})/(1+\chi_{\varepsilon ,out})){\hat{n}}^{/}\times \nabla U_{f,in}\left(r^{/}\right){|}_{S^{/}}.$$

Relations (A20) and (A21) are the most useful of all versions. First, they clearly address the linear dependence that the *tangential* components of the gradient of the *free* scalar potential, $U_{f}\left(r\right)$, should obey at the *interface* $S^{/}$. Second, they are ready to be applied in any problem. In this way the P-D electric susceptibility, $\chi_{\varepsilon }$, will be introduced in $U_{f}\left(r\right)$, D(r), etc.

Next, we proceed with relation (A7) that becomes:

(A24) $${\hat{n}}^{/}\cdot \left(\chi_{\varepsilon ,out}\nabla U_{f,out}\left(r^{/}\right)-\chi_{\varepsilon ,in}\nabla U_{f,in}\left(r^{/}\right)\right){|}_{S^{/}}=-\sigma_{b}\left(r^{/}\right){|}_{S^{/}}/\varepsilon_{0}.$$

As already discussed above, this relation should only be used to determine the *bound* surface charge density, $\sigma_{b}\left(r^{/}\right){|}_{S^{/}}$, from the *normal* component of the gradient of the *free* scalar potential, $U_{f}\left(r\right)$, in a self-consistent way.

Finally, the third boundary condition, relation (A6), gets:

(A25) $${\hat{n}}^{/}\cdot \left(\nabla U_{f,out}\left(r^{/}\right)-\nabla U_{f,in}\left(r^{/}\right)\right){|}_{S^{/}}=-\sigma_{f}\left(r^{/}\right){|}_{S^{/}}/\varepsilon_{0}\;.$$

This relation should be used in close connection with the above relation (A24) to ultimately determine $\sigma_{b}\left(r^{/}\right){|}_{S^{/}}$ from all other known sources.

Now, we have at hand all information that we need on the boundary conditions for the calculation of both $D\left(r\right)$ and $U_{f}\left(r\right)$ by using the strategies presented in the article. Thus, we have completed the investigation for the case where $D\left(r\right)$ and $U_{f}\left(r\right)$ depend on both the *free* charges and the dielectric properties of the LHI dielectrics.

Finally, we discuss the case where $D\left(r\right)$ and $\tilde{P}\left(r\right)=-P(r)$ are exclusively *normal* to the *interface* $S^{/}$. Now, the numerator of the integral in relation (A18) is zero so that $A_{b}\left(r\right)=0$. Thus, $D\left(r\right)$ and $U_{f}\left(r\right)$ are formally coupled through:

(A26) $$D\left(r\right)=-\varepsilon_{0}\nabla U_{f}\left(r\right)\;.$$

In this case, $D\left(r\right)$ is irrotational and $U_{f}\left(r\right)$ is continuous in the entire space, that is, not only at the *interior* of dielectric media, but also at the *interface* $S^{/}$ (even when discontinuous change of the dielectric properties exists at $S^{/}$). In addition, the electric displacement, $D\left(r\right)$, and the *free* scalar potential, $U_{f}\left(r\right)$, depend solely on *free* charges. To calculate $D\left(r\right)$ and $U_{f}\left(r\right),$ we can employ any of the relevant strategies presented in the article. Referring to the boundary conditions, obviously, relations (A10)–(A14) on $D\left(r\right)$ and (A19)–(A23) on $U_{f}\left(r\right)$, which refer to the *tangential* components, are trivially satisfied and no longer can help to mathematically tackle the physical problem. On the contrary, relations (A15)–(A16) on $D\left(r\right)$ and (A24)–(A25) on $U_{f}\left(r\right)$, which refer to the *normal* components, still hold. Most importantly, in place of the non-active relations (A19)–(A23), we can employ the boundary condition on the continuity of $U_{f}\left(r\right)$ at the *interface* $S^{/}$. This property of $U_{f}\left(r\right)$ is now recovered due to the absence of the *tangential* components of $D\left(r\right)$. Thus, in this case, the following very useful relation holds:

(A27) $$U_{f,out}\left(r^{/}\right){|}_{S^{/}}=U_{f,in}\left(r^{/}\right){|}_{S^{/}}.$$

The above discussion is clarified in great detail through some representative problems of electrostatics presented in Section 7 of the article and in the following Appendix A.2 of the Appendix A. There, both cases are considered for $D\left(r\right)$ and $U_{f}\left(r\right)$, when they depend solely on *free* charges and when they also depend on the dielectric properties.

Finally, let us make two comments relevant to this section. First, above we applied our mathematical considerations to the newly introduced P-D, $\chi_{\varepsilon }$, formulation to clarify whether $D\left(r\right)$ depends solely on the *free* charges or it also depends on the dielectric properties. With minor algebraic modifications, the same investigation can be applied to the standard P-E, $\chi_{e}$, formulation, as well. Second, we underline that a relevant argumentation as the one mentioned above for $D\left(r\right)$, holds for the magnetic field $H\left(r\right)$ in the case of magnetostatics: under specific circumstances, $H\left(r\right)$ does not depend only on the *free* current density, it depends on the magnetic properties of the LHI materials as well. These issues will be addressed elsewhere.

### Appendix A.2. Representative Problems in LHI Dielectrics

Following a basic example presented in Section 7 of the article, in this section we survey some representative problems of electrostatics which we address with both formulations, the standard P-E, $\chi_{e}$, and the alternative P-D, $\chi_{\varepsilon }$. With this, we aim to clarify the following issues which are important for the documentation of the P-D, $\chi_{\varepsilon }$, description introduced here: (i) the somehow misleading causality/feedback between the electric polarization, $P\left(r\right)$, and electric field, $E\left(r\right)$, of the P-E, $\chi_{e}$, formulation, and the conceptual restoration by using the P-D, $\chi_{\varepsilon }$, one; (ii) the nature of the depolarizing field/self field and its connection with the *reverse* polarization in both formulations, P-E, $\chi_{e}$, and P-D, $\chi_{\varepsilon }$; (iii) the dependence of the electric displacement, $D\left(r\right)$, on both the *free* charges and the dielectric properties of the LHI materials in both formulations, P-E, $\chi_{e}$, and P-D, $\chi_{\varepsilon }$; and (iv) the overall quantitative equivalence of the two formulations, P-E, $\chi_{e}$, and P-D, $\chi_{\varepsilon }$.

**Problem (1):** Dielectric LHI sphere of P-E/P-D electric susceptibility $\chi_{e}/\chi_{\varepsilon }$ and radius a is placed with its center at the origin of the spherical coordinate system wherein a point electric charge, Q, is hosted.

#### Appendix A.2.1. Solution Based on the P-E Electric Susceptibility, $\chi_{e}$

The standard formulation based on the P-E electric susceptibility, $\chi_{e}$, focuses on the electric field, $E\left(r\right)$, and the respective scalar potential, $U\left(r\right)$, that relate through $E\left(r\right)=-\nabla U\left(r\right)$. We can use the integral form of Gauss’s law for $E\left(r\right)$, Laplace’s equation for $U\left(r\right),$ etc. The following boundary conditions should hold on $U\left(r\right)$ and $E\left(r\right)$, for the inside (r≤a) and outside (a≤r) spaces, as well as at the interface (r=a) of the two dielectrics (sphere and vacuum): (i) for r=0, both $E^{in}\left(r\right)$ and $U^{in}\left(r\right)$ should diverge as $1/r^{2}$ and 1/r, respectively; (ii) for r→∞, both $E^{out}\left(r\right)$ and $U^{out}\left(r\right)$ should become zero; (iii) $U\left(r\right)$ should be continuous at the interface of the two dielectric media, thus $U^{in}\left(r\right){|}_{r=a}=U^{out}\left(r\right){|}_{r=a}$; and (iv) the normal component of $E\left(r\right)$ should satisfy the relation $\left(E_{out}^{⊥}\left(r\right)-E_{in}^{⊥}\left(r\right)\right){|}_{r=a}=\sigma \left(r\right){|}_{r=a}/\varepsilon_{0,}$ where since $\sigma_{f}\left(r\right){|}_{r=a}=0$ it translates to $\left(E_{out}^{⊥}\left(r\right)-E_{in}^{⊥}\left(r\right)\right){|}_{r=a}=\sigma_{b}\left(r\right){|}_{r=a}/\varepsilon_{0}$. Finally, the extra boundary condition on the continuity of the tangential components of $E\left(r\right)$ (that is $E_{out}^{//}\left(r\right){|}_{r=a}=E_{in}^{//}\left(r\right){|}_{r=a}$), is trivially satisfied in this case.

The above information results in $U^{out}\left(r\right)=Q/4\pi \varepsilon_{0}r$ and $U^{in}\left(r\right)=(Q/4\pi \varepsilon r)$ for the scalar potential, $E^{out}\left(r\right)=(Q/4\pi \varepsilon_{0}r^{2})\hat{r}$ and $E^{in}\left(r\right)=(Q/4\pi \varepsilon r^{2})\hat{r}$ for the electric field, $P^{out}\left(r\right)=0$ and $P^{in}\left(r\right)=(\chi_{e}/\varepsilon_{r})(Q/4\pi r^{2})\hat{r}$ for the electric polarization, and $D^{in}\left(r\right)=D^{out}\left(r\right)=(Q/4\pi r^{2})\hat{r}\;$for the electric displacement.

*Depolarizing field/self field:* The surface density of bound charge that resides at the interface r=a is $\sigma_{b}\left(r\right){|}_{r=a}=\hat{r}\cdot P^{in}\left(r\right){|}_{r=a}=\hat{r}\cdot \left(\chi_{e}\varepsilon_{0}{Ε}^{in}\left(r\right)\right){|}_{r=a}=(\chi_{e}/\varepsilon_{r})(Q/4\pi a^{2})$, while the volume density of the charge is concentrated at r=0 with $\rho_{b}\left(r\right){|}_{r=0}=-\nabla \cdot P^{in}\left(r\right){|}_{r=0}=-(\chi_{e}/\varepsilon_{r})(Q/4\pi )\nabla \cdot (\hat{r}/r^{2}){|}_{r=0}=-(\chi_{e}/\varepsilon_{r})Q\delta (r)$. The relevant *internal* electric field (see [25] of the article) produced by $\sigma_{b}\left(r\right){|}_{r=a}$ and $\rho_{b}\left(r\right){|}_{r=0}$ at the inside space is simply $E_{int}^{in}\left(r\right)=E^{in}\left(r\right)-E_{ext}^{in}\left(r\right)=(Q/4\pi \varepsilon r^{2})\hat{r}-(Q/4\pi \varepsilon_{0}r^{2})\hat{r}=-(\chi_{e}/\varepsilon_{r})(Q/4\pi \varepsilon_{0}r^{2})\hat{r}$. Also, we can easily obtain $E_{int}^{in}\left(r\right)=-(1/\varepsilon_{0})P^{in}\left(r\right)$. We recall that $E_{int}^{in}\left(r\right)$ is the so-called depolarizing field, or self field, that relates to $P^{in}\left(r\right)$, else to the reverse ${\tilde{P}}^{in}\left(r\right)=-P^{in}\left(r\right)$ through $E_{int}^{in}\left(r\right)=(1/\varepsilon_{0}){\tilde{P}}^{in}\left(r\right)$ (see Section 3 of the article and Appendix A.2.2 below).

*Dependence of $D\left(r\right)$ on free charges and dielectric properties:* Clearly, since the electric displacement that relates to the *external* sources (point charge Q placed at r=0 in this case), $D_{ext}\left(r\right)=(Q/4\pi r^{2})\hat{r}$, is *normal* to the interface, r=a, of the two dielectric media (sphere with $\varepsilon =\left(1+\chi_{e}\right)\varepsilon_{0}$ and vacuum with $\varepsilon =\varepsilon_{0}$), we expect that the total $D\left(r\right)$ should depend solely on the *free* charges. Thus, $D\left(r\right)$ should be identical to $D_{ext}\left(r\right)$. Indeed, this is the case, since $D\left(r\right)=D_{ext}\left(r\right)=(Q/4\pi r^{2})\hat{r}$ (see above Appendix A.1 of the Appendix A and Section 5.2 and Section 5.3 of the article).

#### Appendix A.2.2. Solution Based on the P-D Electric Susceptibility, $\chi_{\varepsilon }$

The alternative formulation based on the P-D electric susceptibility, $\chi_{\varepsilon }$, should focus on the electric displacement, $D\left(r\right)$, and the respective *free* scalar potential, $U_{f}\left(r\right)$, that relate through $D\left(r\right)=-\varepsilon_{0}\nabla U_{f}\left(r\right)$. Accordingly, we employ the solution of Laplace equation for the (obviously φ-independent) *free* scalar potential, $U_{f}\left(r\right)$, obtained through the method of separation of variables. For the outside space, a≤r, the solution has the form $U_{f}^{out}\left(r\right)=\sum_{l=0}^{\infty }\left(A_{l}^{out}r^{l}+B_{l}^{out}r^{-\left(l+1\right)}\right)P_{l}\left(cos\theta \right)$, while the inside space, r≤a, reads $U_{f}^{in}\left(r\right)=\sum_{l=0}^{\infty }\left(A_{l}^{in}r^{l}+B_{l}^{in}r^{-\left(l+1\right)}\right)P_{l}\left(cos\theta \right)$. The following boundary conditions should hold on $U_{f}\left(r\right)$ and $D\left(r\right)$, for the inside (r≤a) and outside (a≤r) spaces, as well as at the interface (r=a) of the two dielectrics (sphere and vacuum): (i) for r = 0, $U_{f}^{in}\left(r\right)$ should diverge as 1/r; (ii) for r →∞, $U_{f}^{out}\left(r\right)$ should become zero; (iii) the tangential components of $D\left(r\right)$ should satisfy the relation $\left(D_{out}^{//}\left(r\right)-D_{in}^{//}\left(r\right)\right){|}_{r=a}=-\left({\tilde{P}}_{out}^{//}\left(r\right)-{\tilde{P}}_{in}^{//}\left(r\right)\right){|}_{r=a}$ that since $\tilde{P}\left(r\right)=-P\left(r\right)=\chi_{\varepsilon }D\left(r\right)$ and $\chi_{\varepsilon }^{out}=0$, gets $D_{out}^{//}\left(r\right){|}_{r=a}=(1+\chi_{\varepsilon }^{in})D_{in}^{//}\left(r\right){|}_{r=a}$; and (iv) the normal component of $D\left(r\right)$ should satisfy the condition $\left(D_{out}^{⊥}\left(r\right)-D_{in}^{⊥}\left(r\right)\right){|}_{r=a}=\sigma_{f}\left(r\right){|}_{r=a}$ where since $\sigma_{f}\left(r\right){|}_{r=a}=0$, translates to $D_{out}^{⊥}\left(r\right){|}_{r=a}=D_{in}^{⊥}\left(r\right){|}_{r=a}$. Finally, we recall that in contrast to the inherent continuity of $U\left(r\right)$, the *free* scalar potential, $U_{f}\left(r\right)$, is *not* necessarily continuous at the interface of two dielectric media, as indirectly reflected by the *tangential* components of $D\left(r\right)$ in condition (iii) above (see above Appendix A.1 of the Appendix A and Section 5.2 and Section 5.3 of the article).

The above information results in $U_{f}^{out}\left(r\right)=Q/4\pi \varepsilon_{0}r$ and $U_{f}^{in}\left(r\right)=Q/4\pi \varepsilon_{0}r$ for the *free* scalar potential, while through $D\left(r\right)=-\varepsilon_{0}\nabla U_{f}\left(r\right)$ we get $D^{out}\left(r\right)=D^{in}\left(r\right)=(Q/4\pi r^{2})\hat{r}$ for the electric displacement. In addition, through $\tilde{P}\left(r\right)=-P\left(r\right)=\chi_{\varepsilon }D(r)$ we get ${\tilde{P}}^{out}\left(r\right)=-P^{out}\left(r\right)=0$ and ${\tilde{P}}^{in}\left(r\right)=-P^{in}\left(r\right)=\chi_{\varepsilon }(Q/4\pi r^{2})\hat{r}$ for the electric polarization and finally through $E\left(r\right)=D\left(r\right)/\varepsilon_{\varepsilon }$ we obtain $E^{out}\left(r\right)=(Q/4\pi \varepsilon_{0}r^{2})\hat{r}$, $E^{in}\left(r\right)=(1+\chi_{\varepsilon })(Q/4\pi \varepsilon_{0}r^{2})\hat{r}$ for the electric field.

*Depolarizing field/self field:* The surface density of the bound charge that resides at the interface r=a is $\sigma_{b}\left(r\right){|}_{r=a}=\hat{r}\cdot P^{in}\left(r\right){|}_{r=a}=\hat{r}\cdot \left(-{\tilde{P}}^{in}\left(r\right)\right){|}_{r=a}=\hat{r}\cdot \left(-\chi_{\varepsilon }D^{in}\left(r\right)\right){|}_{r=a}=\hat{r}\cdot (-\chi_{\varepsilon }(Q/4\pi r^{2}))\hat{r}{|}_{r=a}=-\chi_{\varepsilon }(Q/4\pi a^{2})$, while the volume density of the charge concentrated at r = 0 is $\rho_{b}\left(r\right){|}_{r=0}=-\nabla \cdot P^{in}\left(r\right){|}_{r=0}=\nabla \cdot {\tilde{P}}^{in}\left(r\right){|}_{r=0}=\chi_{\varepsilon }(Q/4\pi )\nabla \cdot (\hat{r}/r^{2}){|}_{r=0}=\chi_{\varepsilon }Q\delta (r)$. The relevant *internal* electric field (see [25] of the article) produced by $\sigma_{b}\left(r\right){|}_{r=a}$ and $\rho_{b}\left(r\right){|}_{r=0}$ at the inside space is given by $E_{int}^{in}\left(r\right)=E^{in}\left(r\right)-E_{ext}^{in}\left(r\right)=(1+\chi_{\varepsilon })(Q/4\pi \varepsilon_{0}r^{2})\hat{r}-(Q/4\pi \varepsilon_{0}r^{2})\hat{r}=\chi_{\varepsilon }(Q/4\pi \varepsilon_{0}r^{2})\hat{r}$. Also, we can easily obtain $E_{int}^{in}\left(r\right)=(1/\varepsilon_{0}){\tilde{P}}^{in}\left(r\right)$. We recall that $E_{int}^{in}\left(r\right)$ is the so-called depolarizing field or self field (see Section 3 of the article).

*Dependence of $D\left(r\right)$ on free charges and dielectric properties:* As already discussed above in Appendix A.2.1 for the P-E, $\chi_{e}$, formulation, the electric displacement that relates to the *external* sources, $D_{ext}\left(r\right)=(Q/4\pi r^{2})\hat{r}$, is entirely *normal* to the surface of the dielectric sphere. Accordingly, we expect that the total $D\left(r\right)$ should depend solely on the *free* charges, thus should be identical to $D_{ext}\left(r\right)$. This is expected even for the P-D, $\chi_{\varepsilon }$, formulation discussed here. Indeed, this is observed (see above Appendix A.1 of the Appendix A and Section 5.2 and Section 5.3 of the article).

*Non-continuity of the free scalar potential, $U_{f}\left(r\right)$, at the interface r = a:* As discussed above and in the article, when $P\left(r\right)$ is entirely *normal* at an interface, $D\left(r\right)$ and $U_{f}\left(r\right)$ should preserve the irrotational and continuous character, respectively, not only in the interior of dielectrics but also at the respective interfaces (see above Appendix A.1 of the Appendix A and Section 5.2 and Section 5.3 of the article). In the present problem, we have ${\tilde{P}}^{out}\left(r\right)=-P^{out}\left(r\right)=0$ and ${\tilde{P}}^{in}\left(r\right)=-P^{in}\left(r\right)=\chi_{\varepsilon }(Q/4\pi r^{2})\hat{r}$ that is *normal* to the interface r=a. Thus, we expect that $U_{f}\left(r\right)$ should be continuous at this site. Indeed, this is the case since $U_{f}^{out}\left(r\right){|}_{r=a}=U_{f}^{in}\left(r\right){|}_{r=a}=Q/4\pi \varepsilon_{0}a$.

*Comparison between the P-E, $\chi_{e}$, and P-D, $\chi_{\varepsilon }$, formulations:* The two descriptions, P-E, $\chi_{e}$, and P-D, $\chi_{\varepsilon }$, should be equivalent on a quantitative basis. To this effect, it is expected that when we substitute $\chi_{\varepsilon }=-\chi_{e}/(1+\chi_{e})$ (relation (34) of the article) in the expressions obtained here in Appendix A.2.2, we should get the exact same relations obtained above in Appendix A.2.1. Indeed, this can be easily confirmed for all electric entities: displacement, $D\left(r\right)$, polarization, $P\left(r\right)$, field, $E\left(r\right)$, *free* scalar potential of the outside space (a ≤r), $U_{f}^{out}\left(r\right)$, and *bound* surface charge density, $\sigma_{b}\left(r\right){|}_{r=a}$. In addition, we can easily verify that the relation $U\left(r\right)=U_{f}\left(r\right)+U_{b}\left(r\right)$ holds everywhere in space, where $U_{b}\left(r\right)$ is the *bound* scalar potential that relates to the *reverse* electric polarization, $\tilde{P}\left(r\right)=-P\left(r\right)$, through $\tilde{P}\left(r\right)=-\varepsilon_{0}\nabla U_{b}\left(r\right)$. For instance, at the outside space, a≤r, the relation $U^{out}\left(r\right)=U_{f}^{out}\left(r\right)$ holds, since $U_{b}^{out}\left(r\right)=0$. For the inside space, r≤a, we can easily find $U_{b}^{in}\left(r\right)$ and verify that indeed $U^{in}\left(r\right)=U_{f}^{in}\left(r\right)+U_{b}^{in}\left(r\right)$ (see Section 6 of the article).

#### Appendix A.2.3. Solution Based on the P-E Electric Susceptibility, $\chi_{e}$, by Means of Series

Here we employ a series approach with the standard formulation based on the P-E electric susceptibility, $\chi_{e}$, and focus directly on the electric polarization, $P\left(r\right)$, and field, $E\left(r\right)$, to clarify their causality/feedback for the inside space, r≤a, of the LHI dielectric sphere.

Suppose that initially (before the dielectric sphere responds to the *external* stimuli) the electric field is simply the one applied *externally*, $(Q/4\pi \varepsilon_{0}r^{2})\hat{r}$. We call it the zeroth-order term of the electric field, $E_{0}^{in}\left(r\right)=(Q/4\pi \varepsilon_{0}r^{2})\hat{r}$. The respective zeroth-order term of the electric polarization is $P_{0}^{in}\left(r\right)=\chi_{e}\varepsilon_{0}E_{0}^{in}\left(r\right)$. As we showed in both Appendix A.2.1 and Appendix A.2.2 above, the sphere of polarization $P\left(r\right)$ produces an *internal* electric field (depolarizing field/self field) $E_{int}^{in}\left(r\right)=-(1/\varepsilon_{0})P^{in}\left(r\right)=(1/\varepsilon_{0}){\tilde{P}}^{in}\left(r\right)$ at the inside space. Thus, the zeroth-order term of the polarization $P_{0}^{in}\left(r\right)$ will produce a first-order term for the *internal* electric field $E_{int,1}^{in}\left(r\right)=-(1/\varepsilon_{0})P_{0}^{in}\left(r\right)$ (notice that the term $E_{int,0}^{in}\left(r\right)$ does not exist; the only zeroth-order electric field term is of *external* origin, $E_{0}^{in}\left(r\right)=E_{0}\hat{z}$). In turn, the first-order term, $E_{int,1}^{in}\left(r\right)$, will induce a first-order term for the polarization $P_{1}^{in}\left(r\right)=\chi_{e}\varepsilon_{0}E_{int,1}^{in}\left(r\right)$ that subsequently will produce a second-order term for the *internal* electric field $E_{int,2}^{in}\left(r\right)=-(1/\varepsilon_{0})P_{1}^{in}\left(r\right)$ and so on. Thus, in general, the (i-1)-order term of the induced polarization is $P_{i-1}^{in}\left(r\right)=\chi_{e}\varepsilon_{0}E_{int,i-1}^{in}\left(r\right)$, while the (i)-order term of the *internal* electric field is $E_{int,i}^{in}\left(r\right)=-(1/\varepsilon_{0})P_{i-1}^{in}\left(r\right)$. Combining the last relations on $P_{i-1}^{in}\left(r\right)$ and $E_{int,i}^{in}\left(r\right),$ we get $E_{int,i}^{in}\left(r\right)=\left(-\chi_{e}\right)E_{int,i-1}^{in}\left(r\right)=\left(-\chi_{e}\right)\left(-\chi_{e}\right)E_{int,i-2}^{in}\left(r\right)=(-\chi_{e})(-\chi_{e})(-\chi_{e})E_{int,i-3}^{in}\left(r\right)=\cdots ={\left(-\chi_{e}\right)}^{i}E_{0}^{in}\left(r\right)$. Accordingly, the total electric field will simply be $E^{in}\left(r\right)=E_{0}^{in}\left(r\right)+E_{int,1}^{in}\left(r\right)+E_{int,2}^{in}\left(r\right)+\cdots +E_{int,i}^{in}\left(r\right)+\cdots =E_{0}^{in}\left(r\right)+(-\chi_{e})E_{0}^{in}\left(r\right)+(-\chi_{e})(-\chi_{e})E_{0}^{in}\left(r\right)+\cdots +{\left(-\chi_{e}\right)}^{i}E_{0}^{in}\left(r\right)+\cdots$, else $E^{in}\left(r\right)=(\sum_{i=0}^{\infty }{\left(-\chi_{e}\right)}^{i})E_{0}^{in}\left(r\right)$. The geometric series results in $\sum_{i=0}^{\infty }{\left(-\chi_{e}\right)}^{i}=1/(1-(-\chi_{e}))=1/(1+\chi_{e})$ so that ultimately $E^{in}\left(r\right)=E_{0}^{in}\left(r\right)/(1+\chi_{e})=E_{0}^{in}\left(r\right)/\varepsilon_{r}$. Since $E_{0}^{in}\left(r\right)=\left(\frac{Q}{4\pi \varepsilon_{0}r^{2}}\right)\hat{r},$ this result is identical to the one obtained in Appendix A.2.1 above, as expected.

The ‘infinite regress of the P-E polarization process’ applies, also, to the *bound* surface charge density, $\sigma_{b}\left(r\right){|}_{r=a}$, that ultimately will be established at the interface, r=a, of the two dielectrics (sphere and vacuum) even for this case, as discussed analytically for a relevant problem in Section 7.3 of the article.

This series-based approach of the ‘infinite regress of the P-E polarization process’ restores, somehow, the conceptually misleading causality/feedback between $P\left(r\right)$ and $E\left(r\right)$ that is inherent in the standard P-E, $\chi_{e}$, formulation (see [8] pages 68 and 76; [13] page 186). However, the serious obstacle discussed analytically for a relevant problem in Section 7.3 of the article still exists: in strict mathematical terms, the above geometric series should converge only when $|-\chi_{e}|<1$ [33], and since by definition $0\leq \chi_{e}<\infty$, the allowed interval should be $0\leq \chi_{e}<1$. Nevertheless, we do not raise any doubts or constraints on the obtained solution of $E^{in}\left(r\right)$ and use it in the entire range, $0\leq \chi_{e}<\infty$. This is one of the inherent ill-defined points of the standard P-E, $\chi_{e}$, formulation. The alternative P-D, $\chi_{\varepsilon }$, formulation ($-1\leq \chi_{\varepsilon }\leq 0$) is free of any misleading argumentation and controversial mathematics from which the standard P-E, $\chi_{e}$ formulation suffers. This has been assessed analytically for a relevant problem in Section 7.3 of the article.

**Problem (2):** Dielectric LHI cylinder of P-E/P-D electric susceptibility $\chi_{e}/\chi_{\varepsilon }$ and radius a has infinite length, is placed along the z-axis of the cylindrical coordinate system, and is subjected to an *external*, uniform electric field along the *x*-axis, $E\left(r\right)=E_{0}\hat{x}$.

#### Appendix A.2.4. Solution Based on the P-E Electric Susceptibility, $\chi_{e}$

The standard formulation based on the P-E electric susceptibility, $\chi_{e}$, focuses on the electric field, $E\left(r\right)$, and the respective scalar potential, $U\left(r\right)$, that relate through $E\left(r\right)=-\nabla U\left(r\right)$. Our calculations will be exclusively focused on $E\left(r\right)$ and $U\left(r\right)$ as well. Accordingly, we employ the solution of Laplace equation for the scalar potential, $U\left(r\right)$, by means of separation of variables. The most general solution, for the outside space (a≤ρ) is $U^{out}\left(r\right)=\left(A_{0}^{out}+B_{0}^{out}ln(\rho )\right)\left(C_{0}^{out}+D_{0}^{out}\varphi \right)\left(E_{0}^{out}+F_{0}^{out}z\right)+\sum_{m=1}^{\infty }\left(A_{m}^{out}\rho^{m}+B_{m}^{out}\rho^{-m}\right)(C_{m}^{out}cos(m\varphi )+D_{m}^{out}sin(m\varphi ))\left(E_{m}^{out}+F_{m}^{out}z\right)$, while for the inside space (ρ≤a) the respective candidate solution is $U^{in}\left(r\right)=\left(A_{0}^{in}+B_{0}^{in}ln(\rho )\right)\left(C_{0}^{in}+D_{0}^{in}\varphi \right)\left(E_{0}^{in}+F_{0}^{in}z\right)+\sum_{m=1}^{\infty }\left(A_{m}^{in}\rho^{m}+B_{m}^{in}\rho^{-m}\right)(C_{m}^{in}cos(m\varphi )+D_{m}^{in}sin(m\varphi ))\left(E_{m}^{in}+F_{m}^{in}z\right)$. The following boundary conditions should hold on $U\left(r\right)$ and $E\left(r\right)$, for the inside (ρ≤a) and outside (a≤ρ) spaces, as well as at the interface (ρ=a) of the two dielectrics (cylinder and vacuum): (i) for ρ=0, $U^{in}\left(r\right)$ should be finite; (ii) for ρ→∞, $U^{out}\left(r\right)$ should be identical to the *external* scalar potential that produces $E\left(r\right)=E_{0}\hat{x}$, that is $U^{out}\left(r\right){|}_{\rho \to \infty }=-E_{0}\rho cos\varphi$; (iii) $U\left(r\right)$ should be continuous at the interface of the two dielectric media, thus $U^{in}\left(r\right){|}_{\rho =a}=U^{out}\left(r\right){|}_{\rho =a}$; and (iv) the normal component of $E\left(r\right)$ should satisfy the relation $\left(E_{out}^{⊥}\left(r\right)-E_{in}^{⊥}\left(r\right)\right){|}_{\rho =a}=\sigma \left(r\right){|}_{\rho =a}/\varepsilon_{0}$ where since $\sigma_{f}\left(r\right){|}_{\rho =a}=0$ it translates to $\left(E_{out}^{⊥}\left(r\right)-E_{in}^{⊥}\left(r\right)\right){|}_{\rho =a}=\sigma_{b}\left(r\right){|}_{\rho =a}/\varepsilon_{0}$. Finally, notice that the extra boundary condition on the continuity of the tangential components of $E\left(r\right)$ (that is $E_{out}^{//}\left(r\right){|}_{\rho =a}=E_{in}^{//}\left(r\right){|}_{\rho =a}$), reproduces the continuity of $U\left(r\right)$ (condition (iii) above), thus, it does not add new information. Below, we briefly proceed with the solution.

Boundary condition (i) gives $B_{0}^{in}=D_{0}^{in}=F_{0}^{in}=0$ and $B_{m}^{in}=F_{m}^{in}=0$ for 1≤m<∞. Thus, for the inside space, the solution gets $U^{in}\left(r\right)=A_{0}^{in}+\sum_{m=1}^{\infty }\rho^{m}(C_{m}^{in}cos(m\varphi )+D_{m}^{in}sin(m\varphi ))$. Here, we have, also, adopted the obvious fact that for this problem, $U^{in}\left(r\right)$ cannot exhibit linear dependence on φ or z.
Boundary condition (ii) gives $B_{0}^{out}=D_{0}^{out}=F_{0}^{out}=0$, $F_{m}^{out}=0$ for 1≤m<∞ and $A_{m}^{out}=0$ for 2≤m<∞. Thus, for the outside space, the solution gets $U^{out}\left(r\right)=A_{0}^{out}-E_{0}\rho cos\varphi +\sum_{m=1}^{\infty }\rho^{-m}(C_{m}^{out}cos(m\varphi )+D_{m}^{out}sin(m\varphi ))$. Here, we have, also, adopted the obvious fact that for this problem, $U^{out}\left(r\right)$ cannot exhibit linear dependence on φ or z.
Boundary condition (iii) gives $C_{m}^{in}a^{m}=C_{m}^{out}a^{-m}$ for 2≤m<∞, $D_{m}^{in}a^{m}=D_{m}^{out}a^{-m}$ for 1≤m<∞, $C_{1}^{in}a=-E_{0}a+C_{1}^{out}a^{-1}$ for m=1 and $A_{0}^{out}=A_{0}^{in}$ for m=0.
Boundary condition (iv) gives $\left(E_{out}^{⊥}\left(r\right)-E_{in}^{⊥}\left(r\right)\right){|}_{\rho =a}=\sigma_{b}\left(r\right){|}_{\rho =a}/\varepsilon_{0}$ that since $\sigma_{b}\left(r\right){|}_{\rho =a}=\hat{\rho }\cdot P_{in}\left(r\right){|}_{\rho =a}=\hat{\rho }\cdot \left(\chi_{e}\varepsilon_{0}E_{in}\left(r\right)\right){|}_{\rho =a}=\chi_{e}\varepsilon_{0}E_{in}^{⊥}\left(r\right){|}_{\rho =a},$ it transforms to $E_{out}^{⊥}\left(r\right){|}_{\rho =a}=\left(1+\chi_{e}\right)E_{in}^{⊥}\left(r\right){|}_{\rho =a}$, else $E_{out}^{⊥}\left(r\right){|}_{\rho =a}=\varepsilon_{r}E_{in}^{⊥}\left(r\right){|}_{\rho =a}$. The latter ultimately provides the desired relation $-(\partial U^{out}\left(r\right)/\partial \rho ){|}_{\rho =a}=\varepsilon_{r}(-(\partial U^{in}\left(r\right)/\partial \rho )){|}_{\rho =a}$ that gives $\varepsilon_{r}C_{m}^{in}a^{m-1}=-C_{m}^{out}a^{-\left(m+1\right)}$ for 2≤m<∞, $\varepsilon_{r}D_{m}^{in}a^{m-1}=-D_{m}^{out}a^{-\left(m+1\right)}$ for 2≤m<∞, $\varepsilon_{r}C_{1}^{in}=-E_{0}-C_{1}^{out}a^{-2}$ for m=1 and $\varepsilon_{r}D_{1}^{in}=-D_{1}^{out}a^{-2}$ for m=1.

The above set of relations results in $U^{out}\left(r\right)=-[1-((\varepsilon_{r}-1)/(\varepsilon_{r}+1))({a/\rho )}^{2}]E_{0}\rho cos\varphi$ (outside space) and $U^{in}\left(r\right)=-(2/(\varepsilon_{r}+1))E_{0}\rho cos\varphi =-(2/(\varepsilon_{r}+1))E_{0}x$ (inside space) for the scalar potential.

Through $E\left(r\right)=-\nabla U\left(r\right)$ we get the respective relations for the electric field $E^{out}\left(r\right)=[1+((\varepsilon_{r}-1)/(\varepsilon_{r}+1))({a/\rho )}^{2}]E_{0}cos\varphi \hat{\rho }-[1-((\varepsilon_{r}-1)/(\varepsilon_{r}+1))({a/\rho )}^{2}]E_{0}sin\varphi \hat{\varphi }$ and $E^{in}\left(r\right)=\left(2/(\varepsilon_{r}+1)\right)E_{0}\hat{x}.$

Once we have found $E\left(r\right)$, we easily calculate the respective electric polarization, $P\left(r\right)$, and electric displacement, $D\left(r\right)$, since they relate to $E\left(r\right)$ through expressions (1) and (2) of the article. Thus, we get $P^{out}\left(r\right)=0$ and $P^{in}\left(r\right)=2\varepsilon_{0}(\chi_{e}/(\varepsilon_{r}+1))E_{0}\hat{x}$ for the electric polarization, while $D^{out}\left(r\right)=\varepsilon_{0}E^{out}\left(r\right)=[1+((\varepsilon_{r}-1)/(\varepsilon_{r}+1))({a/\rho )}^{2}]D_{0}cos\varphi \hat{\rho }-[1-((\varepsilon_{r}-1)/(\varepsilon_{r}+1))({a/\rho )}^{2}]D_{0}sin\varphi \hat{\varphi }$ and $D^{in}\left(r\right)=\varepsilon E^{in}\left(r\right)=2(\varepsilon_{r}/(\varepsilon_{r}+1))D_{0}\hat{x}$, where $\varepsilon_{0}E_{0}\hat{x}=D_{0}\hat{x}$ the *external* electric displacement.

*Depolarizing field/self field:* The surface density of bound charge that resides at the cylinder/vacuum interface ρ=a is given by $\sigma_{b}\left(r\right){|}_{\rho =a}=\hat{\rho }\cdot P^{in}\left(r\right){|}_{\rho =a}=\hat{\rho }\cdot \left(\chi_{e}\varepsilon_{0}{Ε}^{in}\left(r\right)\right){|}_{\rho =a}=2\varepsilon_{0}((\varepsilon_{r}-1)/(\varepsilon_{r}+1))E_{0}cos\varphi$. The relevant *internal* electric field (see [25] of the article) produced by $\sigma_{b}\left(r\right){|}_{\rho =a}$ at the inside space is simply $E_{int}^{in}\left(r\right)=E^{in}\left(r\right)-E_{ext}^{in}\left(r\right)=(2/(\varepsilon_{r}+1))E_{0}\hat{x}-E_{0}\hat{x}=-((\varepsilon_{r}-1)/(\varepsilon_{r}+1))E_{0}\hat{x}$. Also, we can easily obtain $E_{int}^{in}\left(r\right)=-(1/2\varepsilon_{0})P^{in}\left(r\right)$. We recall that $E_{int}^{in}\left(r\right)$ is the so-called depolarizing field, or self field, that relates to $P^{in}\left(r\right)$, else to the reverse ${\tilde{P}}^{in}\left(r\right)=-P^{in}\left(r\right)$ through $E_{int}^{in}\left(r\right)=(1/2\varepsilon_{0}){\tilde{P}}^{in}\left(r\right)$ (see Section 3 of the article, and below Appendix A.2.5 of the Appendix A).

Dependence of $D\left(r\right)$ on free charges and dielectric properties: Here, the electric displacement that relates to the external sources, $D_{ext}\left(r\right)=D_{0}\hat{x}=\varepsilon_{0}E_{0}\hat{x}=\varepsilon_{0}E_{0}cos\varphi \hat{\rho }-\varepsilon_{0}E_{0}sin\varphi \hat{\varphi }$, has component that is tangential to the surface of the dielectric cylinder. Thus, we expect that the total $D\left(r\right)$, except for the free charges, should depend on the properties of the LHI dielectric cylinder (see above Appendix A.1 of the Appendix A and Section 5.2 and Section 5.3 of the article). Indeed, this is what we observe here.

#### Appendix A.2.5. Solution Based on the P-D Electric Susceptibility, $\chi_{\varepsilon }$

The alternative formulation based on the P-D electric susceptibility, $\chi_{\varepsilon }$, should focus on the electric displacement, $D\left(r\right)$, and the respective *free* scalar potential, $U_{f}\left(r\right)$, that relate through $D\left(r\right)=-\varepsilon_{0}\nabla U_{f}\left(r\right)$. Accordingly, we employ the solution of Laplace equation for the *free* scalar potential, $U_{f}\left(r\right)$, obtained by means of separation of variables. For the outside space (a≤ρ) the most general solution has the form $U_{f}^{out}\left(r\right)=\left(A_{0}^{out}+B_{0}^{out}ln(\rho )\right)\left(C_{0}^{out}+D_{0}^{out}\varphi \right)\left(E_{0}^{out}+F_{0}^{out}z\right)+\sum_{m=1}^{\infty }\left(A_{m}^{out}\rho^{m}+B_{m}^{out}\rho^{-m}\right)(C_{m}^{out}cos(m\varphi )+D_{m}^{out}sin(m\varphi ))\left(E_{m}^{out}+F_{m}^{out}z\right)$, while for the inside space (ρ≤a) it is $U_{f}^{in}\left(r\right)=\left(A_{0}^{in}+B_{0}^{in}ln(\rho )\right)\left(C_{0}^{in}+D_{0}^{in}\varphi \right)\left(E_{0}^{in}+F_{0}^{in}z\right)+\sum_{m=1}^{\infty }\left(A_{m}^{in}\rho^{m}+B_{m}^{in}\rho^{-m}\right)(C_{m}^{in}cos(m\varphi )+D_{m}^{in}sin(m\varphi ))\left(E_{m}^{in}+F_{m}^{in}z\right)$. The following boundary conditions should hold on $U_{f}\left(r\right)$ and $D\left(r\right)$, for the inside (ρ≤a) and outside (a≤ρ) spaces, as well as at the interface (ρ=a) of the two dielectrics (cylinder and vacuum): (i) for ρ=0, $U_{f}^{in}\left(r\right)$ should be finite; (ii) for ρ→∞, $U_{f}^{out}\left(r\right)$ should be identical to the *external* scalar potential that produces $D\left(r\right)=D_{0}\hat{z}$ ($D\left(r\right)=\varepsilon_{0}E_{0}\hat{z}$), that is $U_{f}^{out}\left(r\right){|}_{\rho \to \infty }=-E_{0}\rho cos\varphi$; (iii) the tangential components of $D\left(r\right)$ should satisfy the relation $\left(D_{out}^{//}\left(r\right)-D_{in}^{//}\left(r\right)\right){|}_{\rho =a}=-\left({\tilde{P}}_{out}^{//}\left(r\right)-{\tilde{P}}_{in}^{//}\left(r\right)\right){|}_{\rho =a}$ that since $\tilde{P}\left(r\right)=-P\left(r\right)=\chi_{\varepsilon }D\left(r\right)$ and $\chi_{\varepsilon }^{out}=0$, gets $D_{out}^{//}\left(r\right){|}_{\rho =a}=(1+\chi_{\varepsilon }^{in})D_{in}^{//}\left(r\right){|}_{\rho =a};$ and (iv) the normal component of $D\left(r\right)$ should satisfy the condition $\left(D_{out}^{⊥}\left(r\right)-D_{in}^{⊥}\left(r\right)\right){|}_{\rho =a}=\sigma_{f}\left(r\right){|}_{\rho =a}$ where since $\sigma_{f}\left(r\right){|}_{\rho =a}=0$, translates to $D_{out}^{⊥}\left(r\right){|}_{\rho =a}=D_{in}^{⊥}\left(r\right){|}_{\rho =a}$. Finally, notice that in contrast to the inherent continuity of $U\left(r\right)$, the *free* scalar potential, $U_{f}\left(r\right)$, is *not* necessarily continuous at the interface of two dielectric media (see above Appendix A.1 of the Appendix A and Section 5.2 and Section 5.3 of the article). Thus, it is meaningless to ask for a boundary condition on the continuity of $U_{f}\left(r\right)$ at the interface ρ=a. As we will see below, indeed, the $U_{f}\left(r\right)$ is *non* continuous at the interface ρ=a. Next, we briefly proceed with the solution.

Boundary condition (i) gives $B_{0}^{in}=D_{0}^{in}=F_{0}^{in}=0$ and $B_{m}^{in}=F_{m}^{in}=0$ for 1≤m<∞. Thus, for the inside space the solution gets $U_{f}^{in}\left(r\right)=A_{0}^{in}+\sum_{m=1}^{\infty }\rho^{m}(C_{m}^{in}cos(m\varphi )+D_{m}^{in}sin(m\varphi ))$. Here, we have also adopted the obvious fact that for this problem, $U_{f}^{in}\left(r\right)$ cannot exhibit linear dependence on φ or z.
Boundary condition (ii) gives $B_{0}^{out}=D_{0}^{out}=F_{0}^{out}=0$, $F_{m}^{out}=0$ for 1≤m<∞ and $A_{m}^{out}=0$ for 2≤m<∞. Thus, for the outside space, the solution gets $U_{f}^{out}\left(r\right)=A_{0}^{out}-E_{0}\rho cos\varphi +\sum_{m=1}^{\infty }\rho^{-m}(C_{m}^{out}cos(m\varphi )+D_{m}^{out}sin(m\varphi ))$. Here, we have also adopted the obvious fact that for this problem, $U_{f}^{out}\left(r\right)$ cannot exhibit linear dependence on φ or z.

To proceed with boundary conditions (iii) and (iv), we have to calculate the currently available version of $D\left(r\right)$ through $D\left(r\right)=-\varepsilon_{0}\nabla U_{f}\left(r\right)$. We easily get $D^{in}\left(r\right)=-\varepsilon_{0}\sum_{m=1}^{\infty }m\rho^{m-1}(C_{m}^{in}cos(m\varphi )+D_{m}^{in}sin(m\varphi ))\hat{\rho }-\varepsilon_{0}\sum_{m=1}^{\infty }m\rho^{m-1}(-C_{m}^{in}sin(m\varphi )+D_{m}^{in}cos(m\varphi ))\hat{\varphi }$ and $D^{out}\left(r\right)=\varepsilon_{0}{Ε}_{0}cos\varphi \hat{\rho }+\varepsilon_{0}\sum_{m=1}^{\infty }m\rho^{-(m+1)}(C_{m}^{out}cos(m\varphi )+D_{m}^{out}sin(m\varphi ))\hat{\rho }-\varepsilon_{0}{Ε}_{0}sin\varphi \hat{\varphi }-\varepsilon_{0}\sum_{m=1}^{\infty }m\rho^{-(m+1)}(-C_{m}^{out}sin(m\varphi )+D_{m}^{out}cos(m\varphi ))\hat{\varphi }$.

Boundary condition (iii) gives $(1+\chi_{\varepsilon })C_{m}^{in}a^{m-1}=C_{m}^{out}a^{-(m+1)}$ for 2≤m<∞, $(1+\chi_{\varepsilon })D_{m}^{in}a^{m-1}=D_{m}^{out}a^{-(m+1)}$ for 1≤m<∞ and $(1+\chi_{\varepsilon })C_{1}^{in}=-E_{0}+C_{1}^{out}a^{-2}$ for m=1.
Boundary condition (iv) gives $-C_{m}^{in}a^{m-1}=C_{m}^{out}a^{-(m+1)}$ for 2≤m<∞, $-D_{m}^{in}a^{m-1}=D_{m}^{out}a^{-(m+1)}$ for 1≤m<∞ and $-C_{1}^{in}=E_{0}+C_{1}^{out}a^{-2}$ for m=1.

The above set of relations results in $U_{f}^{out}\left(r\right)=-[1+(\chi_{\varepsilon }/(\chi_{\varepsilon }+2))({a/\rho )}^{2}]E_{0}\rho cos\varphi$ (outside space) and $U_{f}^{in}\left(r\right)=-(2/(\chi_{\varepsilon }+2))E_{0}\rho cos\varphi =-(2/(\chi_{\varepsilon }+2))E_{0}x$ (inside space) for the *free* scalar potential.

Through $D\left(r\right)=-\varepsilon_{0}\nabla U_{f}\left(r\right)$ we get the respective relations for the electric displacement $D^{out}\left(r\right)=[1-(\chi_{\varepsilon }/(\chi_{\varepsilon }+2))({a/\rho )}^{2}]D_{0}cos\varphi \hat{\rho }-[1+(\chi_{\varepsilon }/(\chi_{\varepsilon }+2))({a/\rho )}^{2}]D_{0}sin\varphi \hat{\varphi }$ and $D^{in}\left(r\right)=(2/(\chi_{\varepsilon }+2))D_{0}\hat{x}$, where $D_{0}=\varepsilon_{0}E_{0}$.

Once we have found $D\left(r\right)$, we easily calculate the respective *reverse* electric polarization, $\tilde{P}\left(r\right)$, (electric polarization, $P\left(r\right)$) and electric field, $E\left(r\right)$, since they relate to $D\left(r\right)$ through expressions (17) and (18) of the article. Thus, we get ${\tilde{P}}^{out}\left(r\right)=-P^{out}\left(r\right)=0$ and ${\tilde{P}}^{in}\left(r\right)=-P^{in}\left(r\right)=3(\chi_{\varepsilon }/(\chi_{\varepsilon }+2))D_{0}\hat{x}$ for the *reverse* electric polarization, while $E^{out}\left(r\right)=D^{out}\left(r\right)/\varepsilon_{0}=[1-(\chi_{\varepsilon }/(\chi_{\varepsilon }+2))({a/\rho )}^{2}]E_{0}cos\varphi \hat{\rho }-[1+(\chi_{\varepsilon }/(\chi_{\varepsilon }+2))({a/\rho )}^{2}]E_{0}sin\varphi \hat{\varphi }$ and $E^{in}\left(r\right)=D^{in}\left(r\right)/\varepsilon_{\varepsilon }=D^{in}\left(r\right)/(\varepsilon_{0}\varepsilon_{r\varepsilon })=D^{in}\left(r\right)/(\varepsilon_{0}{\left(1+\chi_{\varepsilon }\right)}^{-1})=(1+\chi_{\varepsilon })D^{in}\left(r\right)/\varepsilon_{0}=2((\chi_{\varepsilon }+1)/(\chi_{\varepsilon }+2))E_{0}\hat{x}$ for the electric field.

*Depolarizing field/self field:* The surface density of *bound* charges at ρ = a is $\sigma_{b}\left(r\right){|}_{\rho =a}=\hat{\rho }\cdot P^{in}\left(r\right){|}_{\rho =a}=\hat{\rho }\cdot \left(-{\tilde{P}}^{in}\left(r\right)\right){|}_{\rho =a}=\hat{\rho }\cdot \left(-\chi_{\varepsilon }D^{in}\left(r\right)\right){|}_{\rho =a}=\hat{\rho }\cdot (-(2\chi_{\varepsilon }/(\chi_{\varepsilon }+2))D_{0}\hat{x}){|}_{\rho =a}=-(2\chi_{\varepsilon }/(\chi_{\varepsilon }+2))D_{0}cos\varphi$, where $D_{0}=\varepsilon_{0}E_{0}$. The relevant *internal* electric field (see [25] of the article) produced by $\sigma_{b}\left(r\right){|}_{\rho =a}$ at the inside space is simply $E_{int}^{in}\left(r\right)=E^{in}\left(r\right)-E_{ext}^{in}\left(r\right)=2((\chi_{\varepsilon }+1)/(\chi_{\varepsilon }+2))E_{0}\hat{x}-E_{0}\hat{x}=(\chi_{\varepsilon }/(\chi_{\varepsilon }+2))E_{0}\hat{x}$. Also, we can easily obtain that $E_{int}^{in}\left(r\right)=(1/2\varepsilon_{0}){\tilde{P}}^{in}\left(r\right)=-(1/2\varepsilon_{0})P^{in}\left(r\right)$. We recall that $E_{int}^{in}\left(r\right)$ is the so-called depolarizing field or self field (see Section 3 of the article).

*Dependence of $D\left(r\right)$ on free charges and dielectric properties:* As already discussed above in Appendix A.2.4 for the P-E, $\chi_{e}$, formulation, the electric displacement that relates to the *external* sources, $D_{ext}\left(r\right)=D_{0}\hat{x}=\varepsilon_{0}E_{0}\hat{x}=\varepsilon_{0}E_{0}cos\varphi \hat{\rho }-\varepsilon_{0}E_{0}sin\varphi \hat{\varphi }$, has component that is *tangential* to the surface of the dielectric cylinder. Thus, we expect that the total $D\left(r\right)$, except for the *free* charges, should depend on the properties of the LHI dielectric cylinder (see above Appendix A.1 of the Appendix A and Section 5.2 and Section 5.3 of the article). This is expected even for the P-D, $\chi_{\varepsilon }$, formulation discussed here. Indeed, this is observed here.

*Non-continuity of the free scalar potential, $U_{f}\left(r\right)$, at the interface ρ = a:* The existence of a *tangential* component of $D\left(r\right)$ and $\tilde{P}\left(r\right)=-P\left(r\right)$ at the interface of different dielectric media will result in a *non* irrotational behavior of $D\left(r\right)$, and a *non* continuous character of $U_{f}\left(r\right)$, locally at the interface (see above Appendix A.1 of the Appendix A and Section 5.2 and Section 5.3 of the article). Indeed, here, $D\left(r\right)$ and $\tilde{P}\left(r\right)=-P\left(r\right)$ have a tangential component at the interface ρ=a. By using the expressions found above for $U_{f}^{out}\left(r\right)$ and $U_{f}^{in}\left(r\right),$ we see that $U_{f}^{out}\left(r\right){|}_{\rho =a}=-2((\chi_{\varepsilon }+1)/(\chi_{\varepsilon }+2))E_{0}acos\varphi$, while $U_{f}^{in}\left(r\right){|}_{\rho =a}=-(2/(\chi_{\varepsilon }+2))E_{0}acos\varphi$. The respective discontinuity is $U_{f}^{out}\left(r\right){|}_{\rho =a}-U_{f}^{in}\left(r\right){|}_{\rho =a}=-(2\chi_{\varepsilon }/(\chi_{\varepsilon }+2))E_{0}acos\varphi$.

*Comparison between the P-E, $\chi_{e}$, and P-D, $\chi_{\varepsilon }$, formulations:* The two descriptions, the standard P-E, $\chi_{e}$, employed today and the alternative P-D, $\chi_{\varepsilon }$, introduced here, should be quantitatively equivalent. Thus, all physical entities of electrostatics (scalar potentials, vector fields, dipole moments, bound charge densities, etc.) should be the same irrespectively of which description we use. To this effect, it is expected that when we substitute $\chi_{\varepsilon }=-\chi_{e}/(1+\chi_{e})$ (relation (34) of the article) in the expressions obtained here in Appendix A.2.5, we should get the exact same relations obtained above in Appendix A.2.4. Indeed, this can be easily confirmed for all electric entities, displacement, $D\left(r\right)$, polarization, $P\left(r\right)$, field, $E\left(r\right)$, *free* scalar potential of the outside space (a≤ρ), $U_{f}^{out}\left(r\right)$, and *bound* surface charge density, $\sigma_{b}\left(r\right){|}_{\rho =a}$. In addition, we can easily verify that the relation $U\left(r\right)=U_{f}\left(r\right)+U_{b}\left(r\right)$ holds everywhere in space, where $U_{b}\left(r\right)$ is the *bound* scalar potential that relates to the *reverse* electric polarization, $\tilde{P}\left(r\right)=-P\left(r\right)$, through $\tilde{P}\left(r\right)=-\varepsilon_{0}\nabla U_{b}\left(r\right)$. For instance, at the outside space, a≤ρ, the relation $U^{out}\left(r\right)=U_{f}^{out}\left(r\right)$ holds, since $U_{b}^{out}\left(r\right)=0$. For the inside space, ρ≤a, we can easily find $U_{b}^{in}\left(r\right)$ and verify that, indeed, $U^{in}\left(r\right)=U_{f}^{in}\left(r\right)+U_{b}^{in}\left(r\right)$ (see Section 6 of the article).

#### Appendix A.2.6. Solution Based on the P-E Electric Susceptibility, $\chi_{e}$, by Means of Series

Here we employ a series approach with the standard formulation based on the P-E electric susceptibility, $\chi_{e}$, and focus directly on the electric polarization, $P\left(r\right)$, and field, $E\left(r\right)$, to clarify their causality/feedback for the inside space, ρ≤a, of the LHI dielectric cylinder.

Suppose that initially (before the dielectric cylinder responds to the *external* stimuli), the electric field is simply the one applied *externally*, $E_{0}\hat{z}$. We call it the zeroth-order term of the electric field, $E_{0}^{in}\left(r\right)=E_{0}\hat{z}$. The respective zeroth-order term of the electric polarization, $P_{0}^{in}\left(r\right)$, induced by $E_{0}^{in}\left(r\right)$ is $P_{0}^{in}\left(r\right)=\chi_{e}\varepsilon_{0}E_{0}^{in}\left(r\right)$. As we showed in both Appendix A.2.4 and Appendix A.2.5 above, a uniformly polarized cylinder of polarization $P\left(r\right)$ produces an *internal* electric field (depolarizing field/self field) $E_{int}^{in}\left(r\right)=-(1/2\varepsilon_{0})P^{in}\left(r\right)=(1/2\varepsilon_{0}){\tilde{P}}^{in}\left(r\right)$ at the inside space. Thus, the zeroth-order term of the polarization $P_{0}^{in}\left(r\right)$ will produce a first-order term for the *internal* electric field $E_{int,1}^{in}\left(r\right)=-(1/2)P_{0}^{in}\left(r\right)$ (notice that the term $E_{int,0}^{in}\left(r\right)$ does not exist; the only zeroth-order electric field term is of *external* origin, $E_{0}^{in}\left(r\right)=E_{0}\hat{z}$). In turn, the first-order term, $E_{int,1}^{in}\left(r\right)$, will induce a first-order term for the polarization $P_{1}^{in}\left(r\right)=\chi_{e}\varepsilon_{0}E_{int,1}^{in}\left(r\right)$ that subsequently will produce a second-order term for the *internal* electric field $E_{int,2}^{in}\left(r\right)=-\left(1/2\varepsilon_{0}\right)P_{1}^{in}\left(r\right),$ and so on. Thus, in general, the (i-1)-order term of the induced polarization is $P_{i-1}^{in}\left(r\right)=\chi_{e}\varepsilon_{0}E_{int,i-1}^{in}\left(r\right)$, while the (i)-order term of the *internal* electric field is $E_{int,i}^{in}\left(r\right)=-(1/2\varepsilon_{0})P_{i-1}^{in}\left(r\right)$. Combining the last relations on $P_{i-1}^{in}\left(r\right)$ and $E_{int,i}^{in}\left(r\right)$, we get $E_{int,i}^{in}\left(r\right)=\left(-\left(\chi_{e}/2\right)\right)E_{int,i-1}^{in}\left(r\right)=\left(-\left(\chi_{e}/2\right)\right)\left(-\left(\chi_{e}/2\right)\right)E_{int,i-2}^{in}\left(r\right)=(-(\chi_{e}/2))(-(\chi_{e}/2))(-(\chi_{e}/2))E_{int,i-3}^{in}\left(r\right)=\cdots ={\left(-(\chi_{e}/2)\right)}^{i}E_{0}^{in}\left(r\right)$. Accordingly, the total electric field will simply be $E^{in}\left(r\right)=E_{0}^{in}\left(r\right)+E_{int,1}^{in}\left(r\right)+E_{int,2}^{in}\left(r\right)+\cdots +E_{int,i}^{in}\left(r\right)+\cdots =E_{0}^{in}\left(r\right)+(-(\chi_{e}/2))E_{0}^{in}\left(r\right)+(-(\chi_{e}/2))(-(\chi_{e}/2))E_{0}^{in}\left(r\right)+\cdots +{\left(-(\chi_{e}/2)\right)}^{i}E_{0}^{in}\left(r\right)+\cdots$, else $E^{in}\left(r\right)=(\sum_{i=0}^{\infty }{\left(-(\chi_{e}/2)\right)}^{i})E_{0}^{in}\left(r\right)$. The geometric series results in $\sum_{i=0}^{\infty }{\left(-(\chi_{e}/2)\right)}^{i}=1/(1-(-(\chi_{e}/2)))=1/(1+\chi_{e}/2)$ so that ultimately $E^{in}\left(r\right)=E_{0}^{in}\left(r\right)/(1+\chi_{e}/2)$, else $E^{in}\left(r\right)=(2/(2+\chi_{e}))E_{0}^{in}\left(r\right)$. Since $E_{0}^{in}\left(r\right)=E_{0}\hat{x,}$ this result is identical to the one obtained in Appendix A.2.4 above as expected.

The ‘infinite regress of the P-E polarization process’ applies, also, to the *bound* surface charge density, $\sigma_{b}\left(r\right){|}_{\rho =a}$, that ultimately will be established at the interface, ρ=a, of the two dielectrics (cylinder and vacuum) even for this case, as discussed analytically for a relevant problem in Section 7.3 of the article.

This series-based approach of the ‘infinite regress of the P-E polarization process’ restores, somehow, the conceptually misleading causality/feedback between $P\left(r\right)$ and $E\left(r\right)$ that is inherent in the standard P-E, $\chi_{e}$, formulation (see [8] pages 68 and 76; [13] page 186, and Section 4 of the article). However, the serious obstacle discussed above in Appendix A.2.3 of the Appendix A and in Section 7.3 of the article still exists: in strict mathematical terms, the above geometric series should converge only when $|-\chi_{e}/2|<1$ [33], and since by definition $0\leq \chi_{e}<\infty$, the allowed interval should be $0\leq \chi_{e}<2$. Nevertheless, we do not raise any doubts or constraints on the obtained solution of $E^{in}\left(r\right)$ and use it in the entire range, $0\leq \chi_{e}<\infty$. This is one of the inherent ill-defined points of the standard P-E, $\chi_{e}$, formulation. The alternative P-D, $\chi_{\varepsilon }$, formulation ($-1\leq \chi_{\varepsilon }\leq 0$) is free of any misleading argumentation and controversial mathematics from which the standard P-E, $\chi_{e}$ formulation suffers. This has been assessed analytically for a relevant problem in Section 7.3 of the article.

**Problem (3):** Dielectric LHI cylinder of P-E/P-D electric susceptibility $\chi_{e}/\chi_{\varepsilon }$ and radius a, has infinite length, is placed along the z axis of the cylindrical coordinate system hosting a coaxial, homogeneous, linear charge density, $\lambda_{0}$.

#### Appendix A.2.7. Solution Based on the P-E Electric Susceptibility, $\chi_{e}$

The standard formulation based on the P-E electric susceptibility, $\chi_{e}$, focuses on the electric field, $E\left(r\right)$, and the respective scalar potential, $U\left(r\right)$, that relate through $E\left(r\right)=-\nabla U\left(r\right)$. We can use the integral form of Gauss’s law for $E\left(r\right)$, Laplace’s equation for $U\left(r\right),$ etc. The following boundary conditions should hold on $U\left(r\right)$ and $E\left(r\right)$, for the inside (ρ≤a) and outside (a≤ρ) spaces, as well as at the interface (ρ=a) of the two dielectrics (cylinder and vacuum): (i) for ρ = 0, both $E^{in}\left(r\right)$ and $U^{in}\left(r\right)$ should diverge as 1/ρ and ln(ρ), respectively; (ii) for ρ→∞, both $E^{out}\left(r\right)$ and $U^{out}\left(r\right)$ should become zero; (iii) $U\left(r\right)$ should be continuous at the interface of the two dielectric media, thus $U^{in}\left(r\right){|}_{\rho =a}=U^{out}\left(r\right){|}_{\rho =a}$; and (iv) the normal component of $E\left(r\right)$ should satisfy the relation $\left(E_{out}^{⊥}\left(r\right)-E_{in}^{⊥}\left(r\right)\right){|}_{\rho =a}=\sigma \left(r\right){|}_{\rho =a}/\varepsilon_{0}$ where since $\sigma_{f}\left(r\right){|}_{\rho =a}=0$ it translates to $\left(E_{out}^{⊥}\left(r\right)-E_{in}^{⊥}\left(r\right)\right){|}_{\rho =a}=\sigma_{b}\left(r\right){|}_{\rho =a}/\varepsilon_{0}$. Finally, the extra boundary condition on the continuity of the tangential components of $E\left(r\right)$ (that is $E_{out}^{//}\left(r\right){|}_{\rho =a}=E_{in}^{//}\left(r\right){|}_{\rho =a}$), is trivially satisfied in this case.

The above information results in $U^{out}\left(r\right)=(\lambda_{0}/2\pi \varepsilon_{0})ln(\infty /\rho )$ and $U^{in}\left(r\right)=(\lambda_{0}/2\pi \varepsilon_{0})ln(\infty /a)+(\lambda_{0}/2\pi \varepsilon )ln(a/\rho )$ for the scalar potential, $E^{out}\left(r\right)=(\lambda_{0}/2\pi \varepsilon_{0}\rho )\hat{\rho }$ and $E^{in}\left(r\right)=(\lambda_{0}/2\pi \varepsilon \rho )\hat{\rho }$ for the electric field, $P^{out}\left(r\right)=0$ and $P^{in}\left(r\right)=(\chi_{e}/\varepsilon_{r})(\lambda_{0}/2\pi \rho )\hat{\rho }$ for the electric polarization, and $D^{in}\left(r\right)=D^{out}\left(r\right)=(\lambda_{0}/2\pi \rho )\hat{\rho }\;$for the electric displacement.

*Depolarizing field/self field:* The surface density of the bound charge that resides at the interface ρ=a is $\sigma_{b}\left(r\right){|}_{\rho =a}=\hat{\rho }\cdot P^{in}\left(r\right){|}_{\rho =a}=\hat{\rho }\cdot \left(\chi_{e}\varepsilon_{0}{Ε}^{in}\left(r\right)\right){|}_{\rho =a}=(\chi_{e}/\varepsilon_{r})(\lambda_{0}/2\pi a)$, while the volume density of the bound charge concentrated at ρ=0 is $\rho_{b}\left(r\right){|}_{\rho =0}=-\nabla \cdot P^{in}\left(r\right){|}_{\rho =0}=-(\chi_{e}/\varepsilon_{r})(\lambda_{0}/2\pi )\nabla \cdot (\hat{\rho }/\rho ){|}_{\rho =0}=-(\chi_{e}/\varepsilon_{r})(\lambda_{0}/2\pi )2\pi \delta (\rho )=-(\chi_{e}/\varepsilon_{r})(\lambda_{0}/2\pi )(\delta (\rho )/\rho )$. The relevant *internal* electric field (see [25] of the article) produced by $\sigma_{b}\left(r\right){|}_{\rho =a}$ and $\rho_{b}\left(r\right){|}_{\rho =0}$ at the inside space is simply $E_{int}^{in}\left(r\right)=E^{in}\left(r\right)-E_{ext}^{in}\left(r\right)=(\lambda_{0}/2\pi \varepsilon \rho )\hat{\rho }-(\lambda_{0}/2\pi \varepsilon_{0}\rho )\hat{\rho }=(\lambda_{0}/2\pi \varepsilon_{0}\rho )(\left(1/\varepsilon_{r}\right)-1)\hat{\rho }=(\lambda_{0}/2\pi \varepsilon_{0}\rho )(-\chi_{e}/(\chi_{e}+1))\hat{\rho }$. Also, we can easily obtain $E_{int}^{in}\left(r\right)=-(1/\varepsilon_{0})P^{in}\left(r\right)$. We recall that $E_{int}^{in}\left(r\right)$ is the so-called depolarizing field, or self field, that relates to $P^{in}\left(r\right)$, else to the reverse ${\tilde{P}}^{in}\left(r\right)=-P^{in}\left(r\right)$ through $E_{int}^{in}\left(r\right)=(1/\varepsilon_{0}){\tilde{P}}^{in}\left(r\right)$ (see Section 3 of the article, and below Appendix A.2.8 of the Appendix A).

*Dependence of $D\left(r\right)$ on free charges and dielectric properties:* Clearly, since the electric displacement that relates to the *external* sources (linear charge density $\lambda_{0}$ placed at ρ=0 in this case), $D_{ext}\left(r\right)=(\lambda_{0}/2\pi \rho )\hat{\rho }$, is *normal* to the interface, ρ=a, of the two dielectric media (cylinder with $\varepsilon =\varepsilon_{r}\varepsilon_{0}=\left(1+\chi_{e}\right)\varepsilon_{0}$ and vacuum with $\varepsilon =\varepsilon_{0}$), we expect that the total $D\left(r\right)$ should depend solely on the *free* charges. Thus, $D\left(r\right)$ should be identical to $D_{ext}\left(r\right)$. Indeed, this is the case, since $D\left(r\right)=D_{ext}\left(r\right)=(\lambda_{0}/2\pi \rho )\hat{\rho }$ (see above Appendix A.1 of the Appendix A and Section 5.2 and Section 5.3 of the article).

#### Appendix A.2.8. Solution Based on the P-D Electric Susceptibility, $\chi_{\varepsilon }$

The alternative formulation based on the P-D electric susceptibility, $\chi_{\varepsilon }$, should focus on the electric displacement, $D\left(r\right)$, and the respective *free* scalar potential, $U_{f}\left(r\right)$, that relate through $D\left(r\right)=-\varepsilon_{0}\nabla U_{f}\left(r\right)$. We can use the integral form of Gauss’s law for $E\left(r\right)$, Laplace’s equation for $U\left(r\right)$, etc. The following boundary conditions should hold on $U_{f}\left(r\right)$ and $D\left(r\right)$, for the inside (ρ≤a) and outside (a≤ρ) spaces, as well as at the interface (ρ=a) of the two dielectrics (cylinder and vacuum): (i) for ρ=0, $U_{f}^{in}\left(r\right)$ should diverge as $\ln\left(\rho \right);$ (ii) for ρ→∞, $U_{f}^{out}\left(r\right)$ should become zero; (iii) the tangential components of $D\left(r\right)$ should satisfy the relation $\left(D_{out}^{//}\left(r\right)-D_{in}^{//}\left(r\right)\right){|}_{\rho =a}=-\left({\tilde{P}}_{out}^{//}\left(r\right)-{\tilde{P}}_{in}^{//}\left(r\right)\right){|}_{\rho =a}$ that since $\tilde{P}\left(r\right)=-P\left(r\right)=\chi_{\varepsilon }D\left(r\right)$ and $\chi_{\varepsilon }^{out}=0$, gets $D_{out}^{//}\left(r\right){|}_{\rho =a}=(1+\chi_{\varepsilon }^{in})D_{in}^{//}\left(r\right){|}_{\rho =a}$; and (iv) the normal component of $D\left(r\right)$ should satisfy the condition $\left(D_{out}^{⊥}\left(r\right)-D_{in}^{⊥}\left(r\right)\right){|}_{\rho =a}=\sigma_{f}\left(r\right){|}_{\rho =a}$ where since $\sigma_{f}\left(r\right){|}_{\rho =a}=0$, translates to $D_{out}^{⊥}\left(r\right){|}_{\rho =a}=D_{in}^{⊥}\left(r\right){|}_{\rho =a}$. Finally, we recall that in contrast to the inherent continuity of $U\left(r\right)$, the *free* scalar potential, $U_{f}\left(r\right)$, is *not* necessarily continuous at the interface of two dielectric media, as indirectly reflected by the *tangential* components of $D\left(r\right)$ in condition (iii) above (see above Appendix A.1 of the Appendix A and Section 5.2 and Section 5.3 of the article). However, as we will see below, in this case the *external* electric displacement, $D_{ext}\left(r\right)$, is absolutely normal to the interface ρ=a of the two dielectrics so that, ultimately, $D\left(r\right)=D_{ext}\left(r\right)$.

The above information results in $U_{f}^{out}\left(r\right)=U_{f}^{in}\left(r\right)=(\lambda_{0}/2\pi \varepsilon_{0})ln(\infty /\rho )$ for the *free* scalar potential, while through $D\left(r\right)=-\varepsilon_{0}\nabla U_{f}\left(r\right)$ we get $D^{out}\left(r\right)=D^{in}\left(r\right)=(\lambda_{0}/2\pi \rho )\hat{\rho }$ for the electric displacement. In addition, through $\tilde{P}\left(r\right)=-P\left(r\right)=\chi_{\varepsilon }D(r)$ we get ${\tilde{P}}^{out}\left(r\right)=-P^{out}\left(r\right)=0$ and ${\tilde{P}}^{in}\left(r\right)=-P^{in}\left(r\right)=\chi_{\varepsilon }(\lambda_{0}/2\pi \rho )\hat{\rho }$ for the electric polarization, and finally through $E\left(r\right)=D\left(r\right)/\varepsilon_{\varepsilon }$ we obtain $E^{out}\left(r\right)=(\lambda_{0}/2\pi \varepsilon_{0}\rho )\hat{\rho }$, $E^{in}\left(r\right)=(1+\chi_{\varepsilon })(\lambda_{0}/2\pi \varepsilon_{0}\rho )\hat{\rho }$ for the electric field.

*Depolarizing field/self field:* The surface density of the bound charge that resides at the interface ρ = a is given by $\sigma_{b}\left(r\right){|}_{\rho =a}=\hat{\rho }\cdot P^{in}\left(r\right){|}_{\rho =a}=\hat{\rho }\cdot \left(-{\tilde{P}}^{in}\left(r\right)\right){|}_{\rho =a}=\hat{\rho }\cdot \left(-\chi_{\varepsilon }D^{in}\left(r\right)\right){|}_{\rho =a}=\hat{\rho }\cdot (-\chi_{\varepsilon }(\lambda_{0}/2\pi \rho ))\hat{\rho }{|}_{\rho =a}=-\chi_{\varepsilon }(\lambda_{0}/2\pi a)$, while the volume density of the bound charge concentrated at ρ = 0 is $\rho_{b}\left(r\right){|}_{\rho =0}=-\nabla \cdot P^{in}\left(r\right){|}_{\rho =0}=\nabla \cdot {\tilde{P}}^{in}\left(r\right){|}_{\rho =0}=\chi_{\varepsilon }(\lambda_{0}/2\pi ))\nabla \cdot (\hat{\rho }/\rho ){|}_{\rho =0}=\chi_{\varepsilon }{(\lambda }_{0}/2\pi )2\pi \delta (\rho )=\chi_{\varepsilon }(\lambda_{0}/2\pi )(\delta (\rho )/\rho )$. The relevant *internal* electric field (see [25] of the article) produced by $\sigma_{b}\left(r\right){|}_{\rho =a}$ and $\rho_{b}\left(r\right){|}_{\rho =0}$ at the inside space is given by $E_{int}^{in}\left(r\right)=E^{in}\left(r\right)-E_{ext}^{in}\left(r\right)=(1+\chi_{\varepsilon })(\lambda_{0}/2\pi \varepsilon_{0}\rho )\hat{\rho }-(\lambda_{0}/2\pi \varepsilon_{0}\rho )\hat{\rho }=\chi_{\varepsilon }(\lambda_{0}/2\pi \varepsilon_{0}\rho )\hat{\rho }$. Also, we can easily obtain $E_{int}^{in}\left(r\right)=(1/\varepsilon_{0}){\tilde{P}}^{in}\left(r\right)$. We recall that $E_{int}^{in}\left(r\right)$ is the so-called depolarizing field or self field (see Section 3 of the article).

*Dependence of $D\left(r\right)$ on free charges and dielectric properties:* As already discussed above in Appendix A.2.7 for the P-E, $\chi_{e}$, formulation, the electric displacement that relates to the *external* sources, $D_{ext}\left(r\right)=(\lambda_{0}/2\pi \rho )\hat{\rho }$, is entirely *normal* to the surface of the dielectric cylinder. Accordingly, we expect that the total $D\left(r\right)$ should depend solely on the *free* charges, thus should be identical to $D_{ext}\left(r\right)$. This is expected even for the P-D, $\chi_{\varepsilon }$, formulation discussed here. Indeed, this is observed (see above Appendix A.1 of the Appendix A and Section 5.2 and Section 5.3 of the article).

*Non-continuity of the free scalar potential, $U_{f}\left(r\right)$, at the interface ρ = a:* As discussed above and in the article, when $P\left(r\right)$ is entirely *normal* at an interface, $D\left(r\right)$ and $U_{f}\left(r\right)$ should preserve the irrotational and continuous character, respectively, not only in the interior of dielectrics but also at the respective interfaces (see above Appendix A.1 of the Appendi A and Section 5.2 and Section 5.3 of the article). In the present problem, we have ${\tilde{P}}^{out}\left(r\right)=-P^{out}\left(r\right)=0$ and ${\tilde{P}}^{in}\left(r\right)=-P^{in}\left(r\right)=\chi_{\varepsilon }(\lambda_{0}/2\pi \rho )\hat{\rho }$ that is *normal* to the interface ρ = a. Thus, we expect that $U_{f}\left(r\right)$ should be continuous at this site. Indeed, this is the case since $U_{f}^{out}\left(r\right){|}_{\rho =a}=U_{f}^{in}\left(r\right){|}_{\rho =a}=(\lambda_{0}/2\pi \varepsilon_{0})ln(\infty /a)$.

*Comparison between the P-E, $\chi_{e}$, and P-D, $\chi_{\varepsilon }$, formulations:* The two descriptions, P-E, $\chi_{e}$, and P-D, $\chi_{\varepsilon }$, should be quantitatively equivalent. To this effect, it is expected that when we substitute $\chi_{\varepsilon }=-\chi_{e}/(1+\chi_{e})$ (relation (34) of the article) in the expressions obtained here in Appendix A.2.8, we should get the exact same relations obtained above in Appendix A.2.7. Indeed, this can be easily confirmed for all electric entities: displacement, $D\left(r\right)$, polarization, $P\left(r\right)$, field, $E\left(r\right)$, *free* scalar potential of the outside space (a ≤ρ), $U_{f}^{out}\left(r\right)$, and *bound* surface charge densities, surface $\sigma_{b}\left(r\right){|}_{\rho =a}$ and volume $\rho_{b}\left(r\right){|}_{\rho =0}$. In addition, we can easily verify that the relation $U\left(r\right)=U_{f}\left(r\right)+U_{b}\left(r\right)$ holds everywhere in space, where $U_{b}\left(r\right)$ is the *bound* scalar potential that relates to the *reverse* electric polarization, $\tilde{P}\left(r\right)=-P\left(r\right)$, through $\tilde{P}\left(r\right)=-\varepsilon_{0}\nabla U_{b}\left(r\right)$. For instance, at the outside space, a≤ρ, the relation $U^{out}\left(r\right)=U_{f}^{out}\left(r\right)$ holds, since $U_{b}^{out}\left(r\right)=0$. For the inside space, ρ≤a, we can easily find $U_{b}^{in}\left(r\right)$ and verify that, indeed $U^{in}\left(r\right)=U_{f}^{in}\left(r\right)+U_{b}^{in}\left(r\right)$ (see Section 6 of the article).

#### Appendix A.2.9. Solution Based on the P-E Electric Susceptibility, $\chi_{e}$, by Means of Series

Here we employ a series approach with the standard formulation based on the P-E electric susceptibility, $\chi_{e}$, and focus directly on the electric polarization, $P\left(r\right)$, and field, $E\left(r\right)$, to clarify their causality/feedback for the inside space, ρ≤a, of the LHI dielectric cylinder.

Suppose that initially (before the dielectric cylinder responds to the *external* stimuli), the electric field is simply the one applied *externally*, $(Q/4\pi \varepsilon_{0}r^{2})\hat{r}$. We call it the zeroth-order term of the electric field, $E_{0}^{in}\left(r\right)=(\lambda_{0}/2\pi \varepsilon_{0}\rho )\hat{\rho }$. The respective zeroth-order term of the electric polarization is $P_{0}^{in}\left(r\right)=\chi_{e}\varepsilon_{0}E_{0}^{in}\left(r\right)$. As we showed in both Appendix A.2.7 and Appendix A.2.8 above, a uniformly polarized cylinder of polarization $P\left(r\right)$ produces an *internal* electric field (depolarizing field/self field) $E_{int}^{in}\left(r\right)=-(1/\varepsilon_{0})P^{in}\left(r\right)=(1/\varepsilon_{0}){\tilde{P}}^{in}\left(r\right)$ at the inside space. Thus, the zeroth-order term of the polarization $P_{0}^{in}\left(r\right)$ will produce a first-order term for the *internal* electric field $E_{int,1}^{in}\left(r\right)=-(1/\varepsilon_{0})P_{0}^{in}\left(r\right)$ (notice that the term $E_{int,0}^{in}\left(r\right)$ does not exist; the only zeroth-order electric field term is of *external* origin, $E_{0}^{in}\left(r\right)=E_{0}\hat{z}$). In turn, the first-order term, $E_{int,1}^{in}\left(r\right)$, will induce a first-order term for the polarization $P_{1}^{in}\left(r\right)=\chi_{e}\varepsilon_{0}E_{int,1}^{in}\left(r\right)$ that subsequently will produce a second-order term for the *internal* electric field $E_{int,2}^{in}\left(r\right)=-\left(1/\varepsilon_{0}\right)P_{1}^{in}\left(r\right),$ and so on. Thus, in general, the (i-1)-order term of the induced polarization is $P_{i-1}^{in}\left(r\right)=\chi_{e}\varepsilon_{0}E_{int,i-1}^{in}\left(r\right)$, while the (i)-order term of the *internal* electric field is $E_{int,i}^{in}\left(r\right)=-(1/\varepsilon_{0})P_{i-1}^{in}\left(r\right)$. Combining the last relations on $P_{i-1}^{in}\left(r\right)$ and $E_{int,i}^{in}\left(r\right),$ we get $E_{int,i}^{in}\left(r\right)=\left(-\chi_{e}\right)E_{int,i-1}^{in}\left(r\right)=\left(-\chi_{e}\right)\left(-\chi_{e}\right)E_{int,i-2}^{in}\left(r\right)=(-\chi_{e})(-\chi_{e})(-\chi_{e})E_{int,i-3}^{in}\left(r\right)=\cdots ={\left(-\chi_{e}\right)}^{i}E_{0}^{in}\left(r\right)$. Accordingly, the total electric field will simply be $E^{in}\left(r\right)=E_{0}^{in}\left(r\right)+E_{int,1}^{in}\left(r\right)+E_{int,2}^{in}\left(r\right)+\cdots +E_{int,i}^{in}\left(r\right)+\cdots =E_{0}^{in}\left(r\right)+(-\chi_{e})E_{0}^{in}\left(r\right)+(-\chi_{e})(-\chi_{e})E_{0}^{in}\left(r\right)+\cdots +{\left(-\chi_{e}\right)}^{i}E_{0}^{in}\left(r\right)+\cdots$, else $E^{in}\left(r\right)=(\sum_{i=0}^{\infty }{\left(-\chi_{e}\right)}^{i})E_{0}^{in}\left(r\right)$. The geometric series results in $\sum_{i=0}^{\infty }{\left(-\chi_{e}\right)}^{i}=1/(1-(-\chi_{e}))=1/(1+\chi_{e})$ so that ultimately $E^{in}\left(r\right)=E_{0}^{in}\left(r\right)/(1+\chi_{e})=E_{0}^{in}\left(r\right)/\varepsilon_{r}$. Since $E_{0}^{in}\left(r\right)=\left(\lambda_{0}/2\pi \varepsilon_{0}\rho \right)\hat{\rho },$ this result is identical to the one obtained above in Appendix A.2.7, as expected.

The ‘infinite regress of the P-E polarization process’ applies also to the *bound* surface charge density, $\sigma_{b}\left(r\right){|}_{\rho =a}$, that ultimately will be established at the interface, ρ=a, of the two dielectrics (cylinder and vacuum) even for this case, as discussed analytically for a relevant problem in Section 7.3 of the article.

This series-based approach of the ‘infinite regress of the P-E polarization process’ restores, somehow, the conceptually misleading causality/feedback between $P\left(r\right)$ and $E\left(r\right)$ that is inherent in the standard P-E, $\chi_{e}$, formulation (see [8] pages 68 and 76; [13] page 186). However, the serious obstacle discussed above in Appendix A.2.3 and Appendix A.2.6 of the Appendix A and in Section 7.3 of the article still exists: in strict mathematical terms, the above geometric series should converge only when $|-\chi_{e}|<1$ [33], and since by definition $0\leq \chi_{e}<\infty$, the allowed interval should be $0\leq \chi_{e}<1$. Nevertheless, we do not raise any doubts or constraints on the obtained solution of $E^{in}\left(r\right)$ and use it in the entire range, $0\leq \chi_{e}<\infty$. This is one of the inherent ill-defined points of the standard P-E, $\chi_{e}$, formulation. The alternative P-D, $\chi_{\varepsilon }$, formulation ($-1\leq \chi_{\varepsilon }\leq 0$) is free of any misleading argumentation and controversial mathematics from which the standard P-E, $\chi_{e}$ formulation suffers. This has been assessed analytically for a relevant problem in Section 7.3 of the article.

**Problem (4):** Dielectric LHI slab (infinite on the xy-plane) of P-E/P-D electric susceptibility $\chi_{e}/\chi_{\varepsilon }$ and thickness a, is placed normal to the z-axis with its midplane at z = 0 of the cartesian coordinate system and is subjected to an *external*, uniform electric field, $E\left(r\right)=E_{0}\hat{z}$.

#### Appendix A.2.10. Solution Based on the P-E Electric Susceptibility, $\chi_{e}$

The standard formulation based on the P-E electric susceptibility, $\chi_{e}$, focuses on the electric field, $E\left(r\right)$, and the respective scalar potential, $U\left(r\right)$. For the electric field, we easily get $E^{out}\left(r\right)=E_{0}\hat{z}$ for z≤−a/2 and a/2≤z, $E^{in}\left(r\right)=(E_{0}/\varepsilon_{r})\hat{z}$ for −a/2≤z≤a/2. For the scalar potential, with a bit of effort, we get $U^{out}\left(r\right)=-E_{0}z$ for z≤−a/2 and a/2≤z and $U^{in}\left(r\right)=-(E_{0}/\varepsilon_{r})(z+sign(z)(a/2)\chi_{e})$ for −a/2≤z≤a/2, where $sign\left(z\right)=\left\{-1,0,+1\right\}$ when z:{<0,=0,>0}. Also, $P^{out}\left(r\right)=0$ for z≤−a/2 and a/2≤z, $P^{in}\left(r\right)=\chi_{e}\varepsilon_{0}(E_{0}/\varepsilon_{r})\hat{z}$ for −a/2≤z≤a/2 for the electric polarization and $D^{out}\left(r\right)=D^{in}\left(r\right)=D_{0}\hat{z}=\varepsilon_{0}E_{0}\hat{z}$ for −∞<z<∞ for the electric displacement.

*Depolarizing field/self field:* The surface density of bound charges that reside at z=±a/2 is $\sigma_{b}\left(r\right){|}_{z=\pm a/2}=\pm \hat{z}\cdot P^{in}\left(r\right){|}_{z=\pm a/2}=\pm \chi_{e}\varepsilon_{0}(E_{0}/\varepsilon_{r})$. The relevant *internal* electric field (see [25] of the article) produced by $\sigma_{b}\left(r\right){|}_{z=\pm a/2}$ at the inside space is simply $E_{int}^{in}\left(r\right)=E^{in}\left(r\right)-E_{ext}^{in}\left(r\right)=(E_{0}/\varepsilon_{r})\hat{z}-E_{0}\hat{z}=-((\varepsilon_{r}-1)/\varepsilon_{r})E_{0}\hat{z}$. Also, we can easily obtain $E_{int}^{in}\left(r\right)=-(1/\varepsilon_{0})P^{in}\left(r\right)$. We recall that $E_{int}^{in}\left(r\right)$ is the so-called depolarizing field, or self field, that relates to $P^{in}\left(r\right)$, else to the *reverse* ${\tilde{P}}^{in}\left(r\right)=-P^{in}\left(r\right)$ through $E_{int}^{in}\left(r\right)=(1/\varepsilon_{0}){\tilde{P}}^{in}\left(r\right)$ (see Section 3 of the article).

*Dependence of $D\left(r\right)$ on free charges and dielectric properties:* Here, the electric displacement that relates to the *external* sources, $D_{ext}\left(r\right)=D_{0}\hat{z}=\varepsilon_{0}E_{0}\hat{z}$, is entirely *normal* to the interfaces, z=±a/2, of the two dielectric media (slab with $\varepsilon =\left(1+\chi_{e}\right)\varepsilon_{0}$ and vacuum with $\varepsilon =\varepsilon_{0}$). Accordingly, the total $D\left(r\right)$ should depend solely on the *free* charges, thus should be identical to the *external* one, $D_{ext}\left(r\right)$. Indeed, this is what we observe here, $D\left(r\right)=D_{ext}\left(r\right)=D_{0}\hat{z}=\varepsilon_{0}E_{0}\hat{z}$ (see above Appendix A.1 of the Appendix A and Section 5.2 and Section 5.3 of the article).

#### Appendix A.2.11. Solution Based on the P-D Electric Susceptibility, $\chi_{\varepsilon }$

The alternative formulation based on the P-D electric susceptibility, $\chi_{\varepsilon }$, focuses on the electric displacement, $D\left(r\right)$, and the respective *free* scalar potential, $U_{f}\left(r\right)$. We easily get $D^{out}\left(r\right)=D^{in}\left(r\right)=D_{0}\hat{z}=\varepsilon_{0}E_{0}\hat{z}$ for −∞<z<∞ for the electric displacement and $U_{f}^{out}\left(r\right)=U_{f}^{in}\left(r\right)=-E_{0}z=-(D_{0}/\varepsilon_{o})z$ for −∞<z<∞ for the *free* scalar potential. Then, we get $P^{out}\left(r\right)=0$ for z≤−a/2 and a/2≤z, ${\tilde{P}}^{in}\left(r\right)=-P^{in}\left(r\right)=\chi_{\varepsilon }D_{0}\hat{z}$ for −a/2≤z≤a/2 for the electric polarization and $E^{out}\left(r\right)={(D}_{0}/\varepsilon_{0})\hat{z}=E_{0}\hat{z}$ for z≤−a/2 and a/2≤z, $E^{in}\left(r\right)=D^{in}\left(r\right)/\varepsilon_{\varepsilon }=(D_{0}/\varepsilon_{\varepsilon })\hat{z}=(1+\chi_{\varepsilon })(D_{0}/\varepsilon_{o})\hat{z}=(1+\chi_{\varepsilon })E_{0}\hat{z}$ for −a/2≤z≤a/2 for the electric field.

*Depolarizing field/self field:* The surface density of bound charges that reside at z=±a/2 is $\sigma_{b}\left(r\right){|}_{z=\pm a/2}=\pm \hat{z}\cdot P^{in}\left(r\right){|}_{z=\pm a/2}=\pm \hat{z}\cdot (-{\tilde{P}}^{in}\left(r\right)){|}_{z=\pm a/2}=\pm (-\chi_{\varepsilon }D_{0})=\pm (-\chi_{\varepsilon }\varepsilon_{0}E_{0})$. The relevant *internal* electric field (see [25] of the article) produced by $\sigma_{b}\left(r\right){|}_{z=\pm a/2}$ at the inside space is simply $E_{int}^{in}\left(r\right)=E^{in}\left(r\right)-E_{ext}^{in}\left(r\right)=(1+\chi_{\varepsilon })E_{0}\hat{z}-E_{0}\hat{z}=\chi_{\varepsilon }E_{0}\hat{z}$. Also, we can easily obtain $E_{int}^{in}\left(r\right)=(1/\varepsilon_{0}){\tilde{P}}^{in}\left(r\right)=-(1/\varepsilon_{0})P^{in}\left(r\right)$. We recall that $E_{int}^{in}\left(r\right)$ is the so-called depolarizing field, or self field (see Section 3 of the article).

*Dependence of $D\left(r\right)$ on free charges and dielectric properties:* As already discussed above in Appendix A.2.10 for the P-E, $\chi_{e}$, formulation, the electric displacement that relates to the *external* sources, $D_{ext}\left(r\right)=D_{0}\hat{z}=\varepsilon_{0}E_{0}\hat{z}$, is entirely *normal* to the interfaces, z=±a/2, of the two dielectric media (slab with $\varepsilon =\left(1+\chi_{e}\right)\varepsilon_{0}$ and vacuum with $\varepsilon =\varepsilon_{0}$). Accordingly, the total $D\left(r\right)$ should depend solely on the *free* charges, thus should be identical to the *external* one, $D_{ext}\left(r\right)$. Indeed, this is observed in the P-D, $\chi_{\varepsilon }$, description discussed here, $D\left(r\right)=D_{ext}\left(r\right)=D_{0}\hat{z}=\varepsilon_{0}E_{0}\hat{z}$ (see above Appendix A.1 of the Appendix A and Section 5.2 and Section 5.3 of the article).

*Non-continuity of the free scalar potential, $U_{f}\left(r\right)$, at the interfaces z = ±a/2:* As discussed above and in the article, when $P\left(r\right)$ is entirely *normal* at an interface, $D\left(r\right)$ and $U_{f}\left(r\right)$ should preserve the irrotational and continuous character, respectively, not only in the interior of dielectrics but also at the respective interfaces (see above Appendix A.1 of the Appendix A and Section 5.2 and Section 5.3 of the article). In the present problem, we have ${\tilde{P}}^{out}\left(r\right)=-P^{out}\left(r\right)=0$ and ${\tilde{P}}^{in}\left(r\right)=-P^{in}\left(r\right)=\chi_{\varepsilon }D_{0}\hat{z}$ that is *normal* to both interfaces z=±a/2. Thus, we expect that $U_{f}\left(r\right)$ should be continuous at these sites. Indeed, this is the case since $U_{f}^{out}\left(r\right){|}_{z=\pm a/2}=U_{f}^{in}\left(r\right){|}_{z=\pm a/2}=-E_{0}(\pm a/2)$.

*Comparison between the P-E, $\chi_{e}$, and P-D, *$\chi_{\varepsilon }$*, formulations:* The two descriptions, P-E, $\chi_{e}$, and P-D, $\chi_{\varepsilon }$, should be equivalent. To this effect, it is expected that when we substitute $\chi_{\varepsilon }=-\chi_{e}/(1+\chi_{e})$ (relation (34) of the article) in the expressions obtained here in Appendix A.2.11, we should get the exact same relations obtained above in Appendix A.2.10. Indeed, this can be easily confirmed for all electric entities: displacement, $D\left(r\right)$, polarization, $P\left(r\right)$, field, $E\left(r\right)$, *free* scalar potential of the outside space (z≤−a/2 and a/2≤z), $U_{f}^{out}\left(r\right)$, and *bound* surface charge density, $\sigma_{b}\left(r\right){|}_{z=\pm a/2}$. In addition, we can easily verify that everywhere in space $U\left(r\right)=U_{f}\left(r\right)+U_{b}\left(r\right)$, where $U_{b}\left(r\right)$ is the *bound* scalar potential that relates to the *reverse* electric polarization, $\tilde{P}\left(r\right)=-P\left(r\right)$, through $\tilde{P}\left(r\right)=-\varepsilon_{0}\nabla U_{b}\left(r\right)$. For instance, at the outside space, z≤−a/2 and a/2≤z, the relation $U^{out}\left(r\right)=U_{f}^{out}\left(r\right)$ holds, since $U_{b}^{out}\left(r\right)=0$. For the inside space, −a/2≤z≤a/2, we can easily find $U_{b}^{in}\left(r\right)$ and verify that, indeed, $U^{in}\left(r\right)=U_{f}^{in}\left(r\right)+U_{b}^{in}\left(r\right)$ (see Section 6 of the article).

#### Appendix A.2.12. Solution Based on the P-E Electric Susceptibility, $\chi_{e}$, by Means of Series

Here we employ a series approach with the standard formulation based on the P-E electric susceptibility, $\chi_{e}$, and focus directly on the electric polarization, $P\left(r\right)$, and field, $E\left(r\right)$, to clarify their causality/feedback for the inside space, −a/2≤z≤a/2, of the LHI dielectric slab.

Suppose that initially (before the dielectric slab responds to the *external* stimuli) the electric field is simply the one applied *externally*, $E_{0}\hat{z}$. We call it the zeroth-order term of the electric field, $E_{0}^{in}\left(r\right)=E_{0}\hat{z}$. The respective zeroth-order term of the electric polarization is $P_{0}^{in}\left(r\right)=\chi_{e}\varepsilon_{0}E_{0}^{in}\left(r\right)$. As we showed in Appendix A.2.10 above, a uniformly polarized slab of polarization $P\left(r\right)$ produces an *internal* electric field (depolarizing field/self field) $E_{int}^{in}\left(r\right)=-(1/\varepsilon_{0})P^{in}\left(r\right)$ at the inside space. Thus, the zeroth-order term of the polarization $P_{0}^{in}\left(r\right)$ will produce a first-order term for the *internal* electric field $E_{int,1}^{in}\left(r\right)=-(1/\varepsilon_{0})P_{0}^{in}\left(r\right)$ (notice that the term $E_{int,0}^{in}\left(r\right)$ does not exist; the only zeroth-order electric field term is of *external* origin, $E_{0}^{in}\left(r\right)=E_{0}\hat{z}$). In turn, the first-order term, $E_{int,1}^{in}\left(r\right)$, will induce a first-order term for the polarization $P_{1}^{in}\left(r\right)=\chi_{e}\varepsilon_{0}E_{int,1}^{in}\left(r\right)$ that subsequently will produce a second-order term for the *internal* electric field $E_{int,2}^{in}\left(r\right)=-\left(1/\varepsilon_{0}\right)P_{1}^{in}\left(r\right),$ and so on. Thus, in general, the (i-1)-order term of the induced polarization is $P_{i-1}^{in}\left(r\right)=\chi_{e}\varepsilon_{0}E_{int,i-1}^{in}\left(r\right)$, while the (i)-order term of the *internal* electric field is $E_{int,i}^{in}\left(r\right)=-(1/\varepsilon_{0})P_{i-1}^{in}\left(r\right)$. Combining the last relations on $P_{i-1}^{in}\left(r\right)$ and $E_{int,i}^{in}\left(r\right),$ we get $E_{int,i}^{in}\left(r\right)=\left(-\chi_{e}\right)E_{int,i-1}^{in}\left(r\right)=\left(-\chi_{e}\right)\left(-\chi_{e}\right)E_{int,i-2}^{in}\left(r\right)=(-\chi_{e})(-\chi_{e})(-\chi_{e})E_{int,i-3}^{in}\left(r\right)=\cdots ={\left(-\chi_{e}\right)}^{i}E_{0}^{in}\left(r\right)$. Accordingly, the total electric field will simply be $E^{in}\left(r\right)=E_{0}^{in}\left(r\right)+E_{int,1}^{in}\left(r\right)+E_{int,2}^{in}\left(r\right)+\cdots +E_{int,i}^{in}\left(r\right)+\cdots =E_{0}^{in}\left(r\right)+(-\chi_{e})E_{0}^{in}\left(r\right)+(-\chi_{e})(-\chi_{e})E_{0}^{in}\left(r\right)+\cdots +{\left(-\chi_{e}\right)}^{i}E_{0}^{in}\left(r\right)+\cdots$, else $E^{in}\left(r\right)=(\sum_{i=0}^{\infty }{\left(-\chi_{e}\right)}^{i})E_{0}^{in}\left(r\right)$. The geometric series results in $\sum_{i=0}^{\infty }{\left(-\chi_{e}\right)}^{i}=1/(1-(-\chi_{e}))=1/(1+\chi_{e})$ so that ultimately $E^{in}\left(r\right)=(1/(1+\chi_{e}))E_{0}^{in}\left(r\right)=E_{0}^{in}\left(r\right)/\varepsilon_{r}$. Since $E_{0}^{in}\left(r\right)=E_{0}\hat{z,}$ we finally get $E^{in}\left(r\right)=(E_{0}/\varepsilon_{r})\hat{z}$. This result is identical to the one obtained above in Appendix A.2.10.

The ‘infinite regress of the P-E polarization process’ applies, also, to the *bound* surface charge density, $\sigma_{b}\left(r\right){|}_{z=\pm a/2}$, that ultimately will be established at the interfaces, z=±a/2, of the two dielectrics (slab and vacuum) even for this case, as discussed analytically for a relevant problem in Section 7.3 of the article.

This series-based approach of the ‘infinite regress of the P-E polarization process’ restores, somehow, the conceptually misleading causality/feedback between $P\left(r\right)$ and $E\left(r\right)$ that is inherent in the standard P-E, $\chi_{e}$, formulation (see [8] pages 68 and 76; [13] page 186). However, the serious obstacle discussed above in Appendix A.2.3, Appendix A.2.6 and Appendix A.2.9 of the Appendix A and in Section 7.3 of the article, exists in the present case as well: in strict mathematical terms, the above geometric series should converge only when $|-\chi_{e}|<1$ [33], and since by definition $0\leq \chi_{e}<\infty$, the allowed interval should be $0\leq \chi_{e}<1$. Nevertheless, we do not raise any doubts or constraints on the obtained solution of $E^{in}\left(r\right)$ and use it in the entire range, $0\leq \chi_{e}<\infty$. This is one of the inherent ill-defined points of the standard P-E, $\chi_{e}$, formulation. The alternative P-D, $\chi_{\varepsilon }$, formulation ($-1\leq \chi_{\varepsilon }\leq 0$) is free of any misleading argumentation and controversial mathematics from which the standard P-E, $\chi_{e}$ formulation suffers. This has been assessed analytically for a relevant problem in Section 7.3 of the article.

### Appendix A.3. Representative Example on a Physical Parameter in LHI Dielectrics: The Clausius-Mossotti Equation

The Clausius-Mossotti equation relates two basic properties of a LHI dielectric: a microscopic, polarizability α of a representative ‘test molecule’ of those the material comprises of, with a macroscopic, relative permittivity, $\varepsilon_{r}$, of the material. Reference books present many different, and in some cases rather complicate, derivations of the Clausius-Mossotti equation [8,9,11,13,14,15,38]. Most of these derivations treat the problem from an entirely macroscopic point of view; the one-by-one interaction of the neighboring electric dipoles/molecules with the ‘test molecule’ under investigation, one way or another is finally neglected, at least in most cases. Thus, in this relatively simple case, the standard Clausius-Mossotti equation is [8,9,11,13,14,15,38]:

$$\alpha =\frac{3\varepsilon_{0}}{N}\frac{\varepsilon_{r}-1}{\varepsilon_{r}+2}$$

where N is the position-independent density of electric dipoles/molecules (number of entities per unit volume of the homogeneous dielectric).

Here, we present an alternative derivation of the Clausius-Mossotti equation. Instead of investigating what happens inside the ‘spherical cavity’ [8,9,11,13,14,15,38], we are focusing on the processes that take place at the dielectric sphere that has been removed from the specimen.

Specifically, we consider a specimen of the LHI dielectric material of interest in the form of a sphere of radius R, subjected to a homogeneous, *external* electric field $E_{ext}\left(r\right)=E_{0}\hat{z}$. Below, we treat the problem with both formulations, the P-E, $\chi_{e}$, and the P-D, $\chi_{\varepsilon }$.

Standard P-E, $\chi_{e}$, formulation: From the macroscopic point of view, we have to express the electric polarization, $P\left(r\right)$, through the relative permittivity, $\varepsilon_{r}$, of the material. In Section 7.1 of the article, we have treated the exact same case and calculated all necessary physical entities. First, we recall the standard definition for the polarization at the inside space (r≤a) of the LHI dielectric sphere:

(A28) $$P^{in\;}\left(r\right)=\chi_{e}\varepsilon_{0}E^{in\;}\left(r\right)$$

where $E^{in}\left(r\right)$ is the *total* electric field inside the specimen and $\chi_{e}$ is the electric susceptibility. For reasons that will become clear below, we formally call $\chi_{e}$ the *intrinsic* electric susceptibility; $\chi_{e}$ reflects the inherent properties of the material *per se* and not of a specimen of particular shape and size characteristics used in a specific experiment. In addition, since $E^{in}\left(r\right)$ is the *total* field, it takes into account two components: (i) the *external* electric field $E_{ext}\left(r\right)=E_{0}\hat{z}$ that is the stimulus applied to the dielectric sphere; and (ii) the *internal* electric field $E_{int}\left(r\right)$, else depolarizing field or self field, that is, the response of the dielectric sphere to $E_{ext}\left(r\right)$ (for details, see Section 3 and Section 7.1, and [25] of the article). For the inside space (r≤a) of the dielectric sphere, we have:

(A29) $$E^{in}\left(r\right)=E_{ext}^{in}\left(r\right)+E_{int}^{in}\left(r\right)\;.$$

By using relation (A29), relation (A28) transforms to

(A30) $$P^{in}\left(r\right)=\chi_{e}\varepsilon_{0}\left(E_{ext}^{in}\left(r\right)+E_{int}^{in}\left(r\right)\right)=\chi_{e}\varepsilon_{0}E_{ext}^{in}\left(r\right)+\chi_{e}\varepsilon_{0}E_{int}^{in}\left(r\right)\;.$$

In these relations, $E_{ext}^{in}\left(r\right)=E_{0}\hat{z}$ is a component controlled/known during the experiment, while $E_{int}^{in}\left(r\right)$ is a component *not* controlled/known, and in addition *cannot* be measured straightforwardly. This a serious obstacle; to practically obtain $P^{in}\left(r\right)$ through relation (A30), we have to somehow get experimental access to $E_{int}^{in}\left(r\right)$. Else, from the experimental point of view, relations (A28) and (A30) are meaningless.

On the other hand, as mentioned above, $E_{ext}^{in}\left(r\right)$ is a physical entity that is completely controlled/known during the experiment; it is the *external* stimulus applied to the specimen by the user. Thus, if we express $E_{int}^{in}\left(r\right)$ through $E_{ext}^{in}\left(r\right),$ we can ultimately obtain an equation that relates $P^{in\;}\left(r\right)$ and $E_{ext}^{in}\left(r\right)$ exclusively. To this effect, an appropriate physical model should be adopted for $E_{int}^{in}\left(r\right)$ that will enable us to mathematically treat the electrostatic problem in a reliable way. Indeed, in Section 7.1 of the article we obtained:

(A31) $$E_{int}^{in}\left(r\right)=-((\varepsilon_{r}-1)/(\varepsilon_{r}+2))E_{0}\hat{z}=-((\varepsilon_{r}-1)/(\varepsilon_{r}+2))E_{ext}^{in\;}\left(r\right)).$$

Thus, through relation (A31), relation (A30) becomes:

(A32) $$P^{in\;}\left(r\right)=3\varepsilon_{0}\left(\frac{\chi_{e}}{\chi_{e}+3}\right)E_{ext}^{in\;}\left(r\right)$$

else

(A33) $$P^{in\;}\left(r\right)=3\varepsilon_{0}\left(\frac{\varepsilon_{r}-1}{\varepsilon_{r}+2}\right)E_{ext}^{in\;}\left(r\right)\;$$

else

(A34) $$P^{in\;}\left(r\right)=\chi_{e,ext}\varepsilon_{0}E_{ext}^{in\;}\left(r\right)$$

where $E_{ext}^{in}\left(r\right)=E_{0}\hat{z}$ and

(A35) $$\chi_{e,ext}=3\left(\frac{\chi_{e}}{\chi_{e}+3}\right)=\frac{\chi_{e}}{1+N_{z}\chi_{e}}=3\left(\frac{\varepsilon_{r}-1}{\varepsilon_{r}+2}\right)\;$$

is the so-called *extrinsic* susceptibility and $N_{z}=1/3$ is the so-called depolarization (else, depolarizing) factor for the case under discussion where the specimen is a sphere. Here, let us make a comment trying to make the connection between the ideal theoretical expectation (relation (A28)) and the experimental realization (relation (A34)). First, we recall that since the *total* electric field $E^{in}\left(r\right)$ inside the material is not experimentally accessible by any means, relation (A28) is useless in recording the *intrinsic* susceptibility, $\chi_{e}$. On the contrary, relation (A34) is useful in obtaining information on the *extrinsic* susceptibility, $\chi_{e,ext}$, since we are able to experimentally record both $E_{ext}^{in}\left(r\right)=E_{0}\hat{z}$ (it is controlled by the user) and $P^{in\;}\left(r\right)$ (probed through a closely relating voltage/current/capacitance signal). Thus, through the experimentally accessible *extrinsic* susceptibility, $\chi_{e,ext}$, we can eventually obtain the *intrinsic* susceptibility, $\chi_{e}$, through relation (A35) as:

(A36) $$\chi_{e}=\varepsilon_{r}-1=3\left(\frac{\chi_{e,ext}}{3-\chi_{e,ext}}\right)=\left(\frac{\chi_{e,ext}}{1-N_{z}\chi_{e,ext}}\right)\;$$

where $N_{z}=1/3$ is the so-called depolarization factor for the case of a sphere as already defined above.

Here, let us clarify the above situation since (to non-experts) it can possibly appear as a misleading paradox: consider an experiment wherein we apply an *external* electric field $E_{0}\hat{z}$ to a specimen, trying to get information on its dielectric properties, susceptibility, $\chi_{e}$, relative permittivity, $\varepsilon_{r}$, polarizability, α, etc. One could probably expect that the *external* electric field $E_{0}\hat{z}$, applied by the user, would penetrate the specimen (thus, in the inside space $E^{in}\left(r\right)=E_{ext}^{in}\left(r\right)=E_{0}\hat{z}$), and polarize it in an exclusive way in the sense that the following relation should hold $P^{in}\left(r\right)=\chi_{e}\varepsilon_{0}E_{ext}^{in}\left(r\right)=\chi_{e}\varepsilon_{0}E_{0}\hat{z}$, i.e., the polarization of the specimen, $P^{in}\left(r\right)$, should depend *solely* on the *external* electric field, $E_{ext}^{in}\left(r\right)=E_{0}\hat{z}$. However, this is *not* the case. Here, we briefly clarify this issue. After subjection to the *external* electric field, $E_{ext}^{in}\left(r\right)=E_{0}\hat{z}$, the specimen will be polarized. The discontinuity of its polarization, $P^{in}\left(r\right)$, at the sphere-vacuum interface, r=a, will produce a *bound* surface charge density, $\sigma_{b}\left(r\right){|}_{r=a}=\hat{r}\cdot P^{in}\left(r\right){|}_{r=a}=3\varepsilon_{0}((\varepsilon_{r}-1)/(\varepsilon_{r}+2))E_{0}cos\theta$. In turn, $\sigma_{b}\left(r\right){|}_{r=a}$ acts as a *secondary* source that produces the so-called *internal* field (else, depolarizing field or self field), $E_{int}^{in}\left(r\right)$, given by relation (A31) above (the *internal* electric field relates to the polarization through $E_{int}^{in}\left(r\right)=-(1/3\varepsilon_{0})P^{in}\left(r\right)$). Ultimately, the *internal* electric field, $E_{int}^{in}\left(r\right)$, (relation (A31)) adds to the *external* one, $E_{ext}^{in}\left(r\right)$, ($E_{0}\hat{z}$) so that the *total* field inside the specimen (relation (A29)) is given by:

(A37) $$E^{in}\left(r\right)=(3/(\varepsilon_{r}+2))E_{0}\hat{z}=(3/(\chi_{e}+3))E_{0}\hat{z}\;.$$

A detailed description of these issues has been presented in Section 7.1 of the article.

Still, we have to obtain $P^{in}\left(r\right)$ from the microscopic point of view, that is, to express $P^{in}\left(r\right)$ through the polarizability, α, of the electric dipoles/molecules. To this effect, we can define the electric polarization of the material in microscopic terms, through the relation:

(A38) $$P^{in}\left(r\right)=Np$$

where we recall that N is the position-independent density of electric dipoles/molecules, while p is the moment of each electric dipole/molecule. Here, we assume that the material comprises of one kind of electric dipoles/molecules so that p is position-independent, as well. Accordingly, based on relation (A38), $P^{in}\left(r\right)$ should be position-independent, thus homogeneous. Indeed, relations (A32)–(A34) give a consistent result; on the right-hand side all $\chi_{e}$, $\varepsilon_{r},$ and $\chi_{e,ext}$ are position-independent (the dielectric is homogeneous), while $E_{ext}^{in}\left(r\right)$ is position-independent ($E_{0}\hat{z}$), as well.

Now, we have to define p through α. At first glance, we should employ the following relation:

(A39) $$p=\alpha E^{in}\left(r\right)$$

where $E^{in}\left(r\right)$ is the *total* local electric field inside the sphere. Notably, relation (A29) reveals that $E^{in}\left(r\right)$ comprises of two components, the *external*, $E_{\;ext}^{in}\left(r\right)$ and the *internal*, else depolarizing field/self field, $E_{int}^{in}\left(r\right)$. By definition, the polarizability, α, cannot depend on *internal* electric fields, irrespectively of their origin [8,9,11,13,14,15,38]. Thus, in our case, the *internal/depolarizing/self* field $E_{int}^{in}\left(r\right)$ should be excluded so that relation (A39) gets:

(A40) $$p=\alpha E_{ext}^{in}\left(r\right)$$

where, now, $E_{ext}^{in}\left(r\right)$ is the *externally* applied electric field. Accordingly, relations (A38) and (A40) can be combined to provide an expression of $P^{in}\left(r\right)$ through the microscopic parameter of polarizability, α, of the material’s electric dipoles/molecules:

(A41) $$P^{in}\left(r\right)=\alpha NE_{ext}^{in}\left(r\right)\;.$$

Combining relations (A33) and (A41) we finally get

(A42) $$\alpha =\frac{3\varepsilon_{0}}{N}\frac{\varepsilon_{r}-1}{\varepsilon_{r}+2}\;.$$

This is the Clausius-Mossotti equation.

The Alternative P-D, $\chi_{\varepsilon }$, formulation: From the macroscopic point of view, we have to express the electric polarization, $P\left(r\right)$, through the P-D relative permittivity, $\varepsilon_{r\varepsilon }$, of the material. In Section 7.2 of the article, we employed the alternative P-D, $\chi_{\varepsilon }$, formulation and calculated all necessary physical entities. Briefly, first, we recall the standard definition for the *reverse* electric polarization at the inside space (r≤a) of the LHI dielectric sphere:

(A43) $${\tilde{P}}^{in}\left(r\right)=-P^{in\;}\left(r\right)=\chi_{\varepsilon }D^{in}\left(r\right)\;$$

where the *total* electric displacement inside the specimen is:

(A44) $$D^{in}\left(r\right)=(3/(2\chi_{\varepsilon }+3))D_{ext}^{in}\left(r\right)$$

with $D_{ext}^{in}\left(r\right)=D_{0}\hat{z}=\varepsilon_{0}E_{0}\hat{z}=\varepsilon_{0}E_{ext}^{in}\left(r\right)$ the *external* electric displacement (originating from *free* charges) applied by the user.

Combining relations (A43) and (A44), we get:

(A45) $${\tilde{P}}^{in}\left(r\right)=-P^{in\;}\left(r\right)=3(\chi_{\varepsilon }/(2\chi_{\varepsilon }+3))D_{ext}^{in}\left(r\right)\;$$

else

(A46) $$P^{in}\left(r\right)=-{\tilde{P}}^{in}\left(r\right)=-3(\chi_{\varepsilon }/(2\chi_{\varepsilon }+3))D_{ext}^{in}\left(r\right).\;$$

Relation (A41) still defines the electric polarization, $P^{in}\left(r\right)$, in microscopic terms. By using the following version:

(A47) $$P^{in}\left(r\right)=\alpha NE_{ext}^{in}\left(r\right)=\alpha N\left(\frac{D_{ext}^{in}\left(r\right)}{\varepsilon_{0}}\right)=\left(\frac{\alpha }{\varepsilon_{0}}\right)ND_{ext}^{in}\left(r\right)\;$$

we define the D-related polarizability, $\alpha_{D}$, (from the E-related one, α) through:

(A48) $$\alpha_{D}=\frac{\alpha }{\varepsilon_{0}}.$$

Thus, we get the expression of polarization that relate to $D_{ext}^{in}\left(r\right)$ (instead of $E_{ext}^{in}\left(r\right)$):

(A49) $$P^{in}\left(r\right)=\alpha_{D}ND_{ext}^{in}\left(r\right)\;.$$

By combining relations (A46) and (A49) we finally get:

(A50) $$\alpha_{D}=-\frac{3}{N}\frac{\chi_{\varepsilon }}{2\chi_{\varepsilon }+3}.$$

Notice that since $-1\leq \chi_{\varepsilon }\leq 0$, the D-related polarizability, $\alpha_{D}$, is always positive, as it should. Also, starting from (A50), by using the above relation (A48) and $\chi_{\varepsilon }=-\chi_{e}/(1+\chi_{e})$ (relation (34) of the article), we immediately obtain relation (A42), that is the Clausius-Mossotti, as expected. Thus, the P-D, $\chi_{\varepsilon }$, formulation is equivalent to the P-E, $\chi_{e}$, one.
